# Siphon Calculus and Lyapunov Functions for Generalized Lotka–Volterra Systems: A Reaction Networks Perspective

**DOI:** 10.3390/e28090980

**Published:** 2026-09-02

**Authors:** Florin Avram

**Affiliations:** Laboratoire de Mathématiques Appliquées, Université de Pau et des Pays de l’Adour, 64013 Pau, France; avramf3@gmail.com

**Keywords:** block generalized Lotka–Volterra systems, siphons, Volterra–Lyapunov functions, Perron–Frobenius theory, complex balance

## Abstract

Generalized Lotka–Volterra (GLV) systems, with roots in ecology, constitute one of the most studied classes of positive ODEs. Recently, a reaction-network perspective for a generalization useful in mathematical epidemiology, called block GLV systems, was offered by Adenane, Avram and Halanay. These authors developed a “siphon calculus” in which the boundary stability of block GLV equations is studied through (i) invariant faces associated with minimal siphons, (ii) transversal Jacobians, (iii) invasibility expressed via *R*-invasion functions, (iv) relay graphs, (v) exclusion partitions and (vi) Lyapunov functions, without leaving the original state space. Another reaction-network perspective for GLV systems was offered by Rojas La Luz, Yu and Craciun, who developed a global stability theory for positive equilibria by introducing associated polyexponential systems obtained through the logarithmic change of variables $x_{i}=e^{\xi_{i}}$. In these logarithmic coordinates, compatibility classes become affine subspaces and simple quadratic Lyapunov functions establish global convergence of complex-balanced systems. The purpose of the present paper is to combine and compare these two perspectives. Our block GLV results here start with a general Perron–Volterra relay theorem (Theorem 7) and its explicit verification for the rank-one multi-strain class. The theorem constructs a face-adapted Lyapunov function in which resident blocks enter through Perron-weighted entropy terms and missing blocks through positive left Perron functionals, and reduces global convergence to resident and transversal closing conditions together with compactness. For the rank-one block model, the canonical Perron normalization gives the closing terms explicitly on an arbitrary resident support *I*: for every missing block k∉I, the corresponding coefficient has the sign of $R_{k}\left(E_{I}\right)-1$. Hence, the relay-sink conditions $R_{k}\left(E_{I}\right)<1,\;k\notin I,$ verify the transversal closing hypothesis and yield convergence to the resident invariant set selected by the resident closing condition (Corollary 20); when that set consists of a single equilibrium $E_{I}$, the convergence is global to $E_{I}$. For nonlinear scalar GLV systems, Theorem 6 gives an exact characterization of global Volterra admissibility through the Jacobian averaged along the segment joining the positive equilibrium $x^{*}$ to each point *x*: $a\in W\left(x^{*}\right)\Leftrightarrow {\left(x-x^{*}\right)}^{T}\left[A\bar{J}\left(x;x^{*}\right)+\bar{J}{\left(x;x^{*}\right)}^{T}A\right]\left(x-x^{*}\right)\leq 0\;\mathrm{for}\;\mathrm{every}\;x\in R_{>0}^{n}.$ It also identifies the averaged-Jacobian matrix inequality as a sufficient global certificate, shows that uniform diagonal stability of the pointwise Jacobian family is a stronger sufficient condition, and shows, via a nonlinear counterexample, that even strict diagonal stability of $Df(x^{*})$ at the equilibrium does not imply global Volterra admissibility. Then, we give a complex-balanced GLV example for which no positive Volterra weight yields a Lyapunov function on the whole positive orthant (Theorem 11). Thus, complex balance does not imply global Volterra admissibility. Interestingly, Volterra decrease is recovered on the compatibility manifold in this example, which leads to the open question whether such compatibility-restricted Volterra functions exist more generally for complex-balanced GLV systems (Problem 2). Finally, we show that all four logical combinations of complex balance and global Volterra admissibility occur among GLV systems, three of them already among affine systems (Theorem 12).

## 1. Introduction

Generalized Lotka–Volterra (GLV) systems

$$x_{i}^{\prime }=x_{i}f_{i}\left(x\right),\;x_{i}\left(t\right)\geq 0,\;i=1,\ldots ,n,$$

form one of the most widely studied classes of nonlinear, positive differential equations. They arise naturally in population dynamics, ecological communities, evolutionary game theory, epidemic modeling, biochemical interaction networks and many other areas.

Adenane, Avram and Halanay [1] introduced a reaction-network perspective, called siphon calculus, for a generalization of Lotka–Volterra systems useful in mathematical epidemiology, called *block GLV systems*. This supplies boundary-geometric elements including invariant faces, minimal siphons, transversal Jacobians, relay graphs and exclusion partitions, which provide a basis for a computerized approach to block GLV systems.

**Remark** **1** (Block GLV systems and compartmental models)**.** *In compartmental epidemic models, the block formulation is natural. For an invariant siphon face $F_{\sigma }$, the variables which vanish on the face form the transversal subsystem, whose Jacobian is denoted by*

$$J_{\sigma }^{⊥}.$$

*Positivity implies that $J_{\sigma }^{⊥}$ is Metzler, so transversal stability is determined by its spectral bound*

$$s(J_{\sigma }^{⊥}).$$

*Thus*

$$s(J_{\sigma }^{⊥})<0$$

*means that the missing compartments cannot invade the resident equilibrium, whereas $s(J_{\sigma }^{⊥})>0$ means that the face is transversally unstable.*

*When the transversal Jacobian admits the usual regular next-generation splitting [2,3],*

$$J_{\sigma }^{⊥}=F_{\sigma }-V_{\sigma },\;F_{\sigma }\geq 0,$$

*with $V_{\sigma }$ a nonsingular M-matrix, the same threshold can be written as*

$$R_{\sigma }=\rho \left(F_{\sigma }V_{\sigma }^{-1}\right),\;s\left(J_{\sigma }^{⊥}\right)<0\Leftrightarrow R_{\sigma }<1.$$

*Hence, the scalar invasion exponent of a missing species in an ordinary GLV system and the Perron/next-generation threshold of a missing block in a compartmental model play the same mathematical role: both measure the transversal stability of a siphon face. The block formulation places this compartmental threshold within the boundary geometry supplied by siphons, resident faces and relay relations.*

Rojas La Luz, Yu and Craciun [4] introduced a different reaction-network perspective, specific to classic (scalar) generalized Lotka–Volterra systems, transforming them into polyexponential systems by the change of variables

$$x_{i}=e^{\xi_{i}},$$

under which the compatibility geometry straightens into affine subspaces and the Lyapunov function reduces to the Euclidean distance from a steady state,

$$L\left(\xi \right)=\sum_{i}{\left(\xi_{i}-\xi_{i}^{*}\right)}^{2},\;\mathrm{whose}\;\mathrm{pull}-\mathrm{back}\;\mathrm{is}\;V\left(x\right)=\sum_{i}{\left(\log x_{i}-\log x_{i}^{*}\right)}^{2},$$

giving a global stability theorem for complex-balanced generalized Lotka–Volterra systems.

The present paper has a double goal. First, in the framework of classic GLV systems, it compares the Horn–Jackson route with classical *Goh–Volterra Lyapunov (GVL) functions*

$$V\left(x\right)=\sum_{k}a_{k}x_{k}^{*}\left(x_{k}/x_{k}^{*}-1-\log\left(x_{k}/x_{k}^{*}\right)\right),$$

which work directly in the original positive variables and were already studied by Goh [5] and by Rojas La Luz, Yu and Craciun [4]. We study—systematically—the set of weights for which these functions decrease, including its exact affine characterization, its Perron construction in the Metzler case, and the geometry of the resulting admissible-weight cone.

**Remark** **2** (Research gaps and contributions)**.** *The two reaction-network perspectives address complementary questions. Siphon calculus describes invariant boundary faces and their transversal invasion properties, while the E-graph realization describes compatibility manifolds and complex balance. The Volterra problem asks, directly from the GLV vector field, which positive weights give a global Lyapunov function.*

*The paper develops this connection in several directions. For affine GLV systems it gives an exact characterization of Volterra admissibility and a canonical Perron construction for irreducible Metzler interaction matrices. For nonlinear GLV systems it separates the local Jacobian condition from the exact global averaged-Jacobian criterion and gives computable sufficient conditions. For block GLV systems it gives a general Perron–Volterra relay theorem and an explicit verification for the rank-one multi-strain class. Finally, comparison with E-graph realizations separates global Volterra admissibility from complex balance and identifies the corresponding compatibility geometry.*

Some further results, beyond those emphasized in the abstract, are the following.

1. For scalar GLV systems, coordinate-face invariance (Proposition 1) yields a particularly simple siphon geometry: the minimal siphons are the singleton coordinates (Corollary 2), resident subsystems are obtained by restriction to faces, and the transversal Jacobian at a boundary equilibrium is diagonal (Proposition 2), with entries given by the scalar invasion exponents
  $$f_{i}\left(E_{\Sigma }\right).$$
2. The affine Volterra theory is constructive. For
  $$f\left(x\right)=M\left(x-x^{*}\right),$$
  Volterra admissibility is exactly the semidefinite condition
  $$a\in W\left(x^{*}\right)\Leftrightarrow \mathrm{diag}\left(a\right)M+M^{T}\mathrm{diag}\left(a\right)⪯0$$
  (Theorem 3). In the irreducible Metzler case, the Perron vectors give the canonical weight
  $$a_{i}=\frac{\pi_{i}}{w_{i}}$$
  (Theorem 4), while Goh’s diagonal-dominance construction gives another explicit class of admissible weights (Corollary 5).
3. The geometry and functorial properties of the affine admissible-weight cone are developed further. In dimension two, the cone is described exactly (Theorem 5); positive diagonal rescalings act on it explicitly (Proposition 3); and admissible weights restrict to resident subsystems (Proposition 7). These results complement the nonlinear averaged-Jacobian characterization of Theorem 6, emphasized in the abstract.
4. The intrinsic theory is illustrated on two low-dimensional families. For affine two-species competition, the Volterra construction gives the complete exclusion–coexistence partition (Theorem 8), whereas higher-order interactions may create multistationarity absent from the affine case (Theorem 9).
5. After an E-graph realization is fixed, its stoichiometric subspace determines the compatibility manifolds
  $$C\left(x^{0}\right)=\exp\left(\log x^{0}+S\right)$$
  (Proposition 8). The complex-balanced stability theorem is then expressed directly in the original variables (Theorem 10), with deficiency-zero and diagonal-rescaling consequences. This realization-dependent theory provides the setting for the comparison between complex balance and Volterra admissibility developed later in Part II.

These results organize three complementary structures carried by the models considered here.

(1) *Boundary geometry*: invariant coordinate faces, singleton or block siphons, resident subsystems, transversal Jacobians, relay graphs, and competitive exclusion partitions.
(2) *Realization geometry*: first integrals and compatibility manifolds associated with a chosen E-graph realization. Logarithmic coordinates straighten these manifolds into affine subspaces.
(3) *Volterra–Lyapunov geometry*: Volterra functions in the original variables and log-quadratic functions associated with complex balance. The admissible-weight cone records the Volterra certificates, while the stoichiometric subspace records the compatibility classes associated with the chosen realization.

**Remark** **3** (Three complementary structures)**.** *The boundary geometry and Volterra admissibility are determined directly from the GLV vector field. The realization geometry uses the additional choice of an E-graph realization and its stoichiometric subspace. The paper compares the two resulting Lyapunov mechanisms: Volterra admissibility gives a certificate in the original positive variables, while complex balance gives a realization-dependent certificate on compatibility manifolds. This separation between intrinsic and realization-dependent structures is the organizing principle of Parts I and II.*

The paper is guided by three questions:

(Q1) Which boundary and stability structures are determined directly by the GLV vector field, and which require a chosen E-graph realization?
(Q2) For which GLV systems does a Volterra Lyapunov function give global convergence?
(Q3) How can the Volterra weights be computed, characterized, or selected canonically from the structure of the model?

### Guide to the Paper

The paper separates three structures associated with generalized Lotka–Volterra (GLV) systems

$${\dot{x}}_{i}=x_{i}f_{i}\left(x\right):$$

*boundary geometry*, determined by the positive vector field and its invariant coordinate faces; *Volterra–Lyapunov geometry*, determined directly by the vector field through its admissible Lyapunov weights; and *realization geometry*, obtained after a Euclidean-graph realization is chosen and expressed through its stoichiometric subspace, first integrals and compatibility manifolds. Accordingly, the paper is divided into two parts. Part I studies structures intrinsic to the GLV vector field. Part II fixes an additional Euclidean-graph realization

$$f\left(x\right)=YA_{\kappa }x^{Y},$$

and studies the resulting Kirchhoff matrix, stoichiometric subspace, compatibility foliation and complex-balance structure. Different realizations of the same GLV vector field may carry different stoichiometric data while representing the same underlying ODE.

Section 3 develops the boundary geometry of GLV systems. Coordinate-face invariance characterizes the multiplicative GLV form (Proposition 1), the minimal siphons are the singleton coordinates under the stated boundary regularity (Corollary 2), and the transversal Jacobian at a boundary equilibrium is diagonal (Proposition 2), with scalar invasion exponents $f_{i}\left(E_{\Sigma }\right)$. Thus, the scalar relay graph is determined by invasion signs; its Lyapunov realization is obtained later from Theorem 7.

Section 4 develops the Volterra Lyapunov theory. For affine systems,

$$a\in W\left(x^{*}\right)\Leftrightarrow \mathrm{diag}\left(a\right)M+M^{⊤}\mathrm{diag}\left(a\right)⪯0$$

(Theorem 3), reducing admissibility to a semidefinite feasibility problem. For irreducible Metzler matrices, Theorem 4 gives the canonical Perron weight

$$a_{i}=\frac{\pi_{i}}{w_{i}},$$

together with an explicit graph-Laplacian dissipation identity.

Section 5 studies the admissible-weight cone and the passage from local to global Volterra conditions. The cone is characterized exactly in dimension two (Theorem 5), while Proposition 6 separates Hurwitz stability from diagonal stability. Theorem 6 gives the exact nonlinear criterion in terms of the Jacobian averaged along the segment from $x^{*}$ to *x*, together with stronger pointwise sufficient conditions and a counterexample showing that diagonal stability of $Df(x^{*})$ alone is insufficient.

The same section then passes from scalar GLV systems to block models. Theorem 7 gives the general Perron–Volterra relay theorem on a siphon face. Corollary 20 verifies its closing mechanism explicitly for the rank-one multi-strain class, while Corollary 21 gives its affine scalar specialization. Proposition 7 describes how admissible affine weights restrict to resident subsystems.

Section 6 illustrates the intrinsic theory on two two-species GLV families. Theorem 8 gives the complete exclusion–coexistence partition for the affine weak-competition family, while Theorem 9 shows how higher-order interactions may generate multistationarity.

Part II begins with Section 7, where a Euclidean-graph realization

$$f\left(x\right)=YA_{\kappa }x^{Y}$$

is fixed. The realization supplies a Kirchhoff matrix and a stoichiometric subspace and hence an associated family of invariant compatibility manifolds. Since realizations are generally nonunique (Remark 32), these objects belong to the chosen realization. Every such realization represents the same GLV vector field, so the trajectories of the ODE are unchanged; the nonuniqueness concerns the additional stoichiometric and compatibility structure used to organize those trajectories.

Section 8 develops this realization geometry. Proposition 8 constructs the first integrals and compatibility manifolds generated by the stoichiometric subspace. Theorem 10 gives the Rojas La Luz–Yu–Craciun complex-balanced stability result directly in the original variables, with its deficiency-zero and diagonal-rescaling consequences.

The comparison between the two Lyapunov mechanisms is completed in Section 8: Theorem 11 gives a complex-balanced GLV system with

$$W(x^{*})=⌀,$$

showing that complex balance does not imply global Volterra admissibility and motivating the compatibility-restricted question of Problem 2.

Finally, Section 9 revisits representative examples of Rojas La Luz, Yu and Craciun and compares their Volterra and complex-balance structures. The section culminates in Theorem 12, which realizes all four logical combinations of complex balance and global Volterra admissibility.

The conclusions summarize the principal intrinsic, block and realization-dependent results and the open problems suggested by their comparison.

## 2. Some CRN, ME and Ecology Terminology, Concepts and Results

Population dynamics, ecology, mathematical epidemiology (ME), virology, social networks and chemical reaction network theory (CRNT) are a few of the biological interaction networks (BIN) subfields, which study all positive dynamical systems and have similar preoccupations: the existence and multiplicity of equilibria, their local and global stability, the occurrence of bifurcations, persistence, permanence, extinction, etc.

**Definition** **1** (Positive/nonnegative ODE [Pos])**.** *An ODE is called positive [6] or nonnegative [7] if the nonnegative orthant*

$$R_{\geq 0}^{n}:=\left\{x\in R^{n}:x_{i}\geq 0,\;i=1,\ldots ,n\right\}$$

*is forward invariant under the flow.*

The first characteristic of most models in mathematical epidemiology, ecology, and immuno-virology is that they are positive ODEs (or stochastic models whose mean field is a positive ODE). This means that important phenomena occur when trajectories reach the boundary of the positive orthant, and that the notion of invariant boundary faces is crucial. Invariant boundary faces depend on the dynamics under study, but the sets of coordinates which are zero on a boundary face enjoy a “structural independence” of the dynamics, as long as the rates are modified so that the natural properties (like increasingness) in Definition 2 below hold. This suggests that siphons (a Petri network concept) are even more fundamental for positive ODEs than invariant boundary faces.

While (invariant) boundary faces have been studied in classical ODE theory, the systematic exploitation of simplifications arising for positive ODEs was initiated by CRN works; see notably [8,9], who showed that all positive polynomial models admit (non-unique) mass-action representations. Later, ref. [10] discovered the connection between invariant boundary faces and siphons.

Let us note now a recent proof in [1,11], reproduced here as Theorem 1 of the folklore result that Jacobians sometimes have a triangular block, which sometimes was replaced by siphon faces. Also, ref. [12] proved that the transversal block of a Jacobian on a siphon face has the Metzler/cooperativity property, Theorem 2 below, which further ensures the existence of left and right Perron eigenvectors. These results explain the numerous appearances of these vectors in ME papers (see for example [13,14], where they provide Lyapunov functions and escape directions from siphon faces, respectively) and suggest that siphons are useful organizing tools for positive ODEs; we call this organizing principle informally “siphon calculus”.

Note that both classical Lotka–Volterra (LV) theory and its generalization studied below, “block/vector” Lotka–Volterra theory, which replaces scalar factored equations by vectorial factored equations, are particular cases of siphon calculus where the minimal siphons are disjoint. Siphon calculus supplies a common organization of scalar and block systems according to their minimal-siphon structure, classifying positive ODEs into easy and research territory according to whether the minimal siphons are disjoint or not.

Let us note that due to the different examples they focused on, the positive ODE subfields diverge somewhat in their terminology and methods. Concerning examples, note that models without boundary attractors may be acceptable in CRNT, but not in ME and ecology, and reversibility, very frequent in CRNT, is absent in ME and ecology.

Our CRN-inspired framework is encapsulated by the following definitions.

**Definition** **2** (Stoichiometric and chemical representation [crn])**.** *A stoichiometric representation of f is a pair (Γ,r) such that*

f(x)=Γr(x),r(x)≥0,

*where $\Gamma \in R^{n\times n_{R}}$ is constant and $r:R_{\geq 0}^{n}\to R_{\geq 0}^{n_{R}}$ is locally Lipschitz.*

*An ODE is called chemical if*

$$\Gamma_{i\rho }<0\Rightarrow r_{\rho }\left(x\right)=x_{i}{\tilde{r}}_{\rho }\left(x\right),\;{\tilde{r}}_{\rho }\left(x\right)\geq 0,$$

*and, moreover, $r_{\rho }$ depends only on variables in $\mathrm{supp}\left(r_{\rho }\right)\subseteq \Gamma_{i}^{-}$ whenever $\Gamma_{i\rho }<0$. Furthermore, we assume that each rate $r_{\rho }\left(x\right)$ is monotone non-decreasing with respect to each variable $x_{k}$ for $k\in \mathrm{supp}(r_{\rho })$.*

Siphons.

Angeli, de Leenheer and Sontag [10], Anderson [15] and Shiu and Sturmfels [16] (Prop. 2.1) proved that a boundary face $F_{\sigma }$ associated to a nonempty set σ of variables being 0 is forward-invariant for a chemical ODE iff σ is a siphon in the sense of the combinatorial Definition 3:

**Definition** **3** (Siphon, minimal siphon, total siphon/DFE, critical siphon [Sip])**.**

- *A siphon set σ⊆S is a nonempty subset of species such that whenever a species in σ appears in a product complex in the RHS of a reaction, at least one species in σ must also appear in the LHS of the reaction (the reactant complex).*
- *A siphon is minimal when it contains no other siphon.*
- *The union of all minimal siphons will be called the total siphon/DFE and denoted $\sigma_{0}$.*
- *A siphon σ in a CRN with stoichiometric matrix* Γ *is critical if it contains no support of a positive conservation relation, i.e., if there exists no nonzero vector c≥0 with cΓ=0 and suppc⊂σ.*

**Remark** **4** (CRNT suggests definitions for important ME concepts)**.** *The total siphon/DFE set $\sigma_{0}$ is a fundamental object of study in mathematical epidemiology (ME), but a rigorous definition for it is available only via CRNT. Similarly, CRNT suggests definitions for multi-strain and ME models—see below.*

We now have the building bricks to define ME and multi-strain systems.

**Definition** **4** (ME models, *k*–siphon models, *k*–strain simple models [StrMod])**.** *Consider a chemical ODE with at least one critical minimal siphon and a unique DFE, and let $J_{0}^{⊥}$ denote the diagonal Jacobian block in the triangularization of the Jacobian corresponding to the DFE siphon, which exists and is Metzler by [1,11,12], i.e., by Theorems 1 and 2.*

*An epidemic model is a pair formed from a chemical ODE and a regular splitting (RS) $J_{0}^{⊥}=F_{0}-V_{0}$ of the transversal Jacobian $J_{0}^{⊥}$ in the sense of [2,17] (with $F_{0}\geq 0$ and $V_{0}$ a nonsingular M-matrix, hence $V_{0}^{-1}\geq 0$). Since $J_{0}^{⊥}$ is Metzler [11], such a splitting always exists: write $J_{0}^{⊥}=F+D$ with $F:=J_{0}^{⊥}-\mathrm{diag}\left(J_{0}^{⊥}\right)\geq 0$ and $D:=\mathrm{diag}(J_{0}^{⊥})$; then, for every $c>\max\{0,\max_{i}J_{0,ii}^{⊥}\}$,*

$$J_{0}^{⊥}=F_{c}-V_{c},\;F_{c}:=F+cI\geq 0,\;V_{c}:=cI-D,$$

*is a regular splitting, since $V_{c}$ is diagonal with strictly positive entries $c-J_{0,ii}^{⊥}$, hence a nonsingular M-matrix. In particular, c=0 already works whenever every diagonal entry of $J_{0}^{⊥}$ is strictly negative, recovering $F_{0}=F$, $V_{0}=-D$.*

*Such a splitting is never unique: once $J_{0}^{⊥}=F-V$ is a regular splitting, then for every c>0,*

$$J_{0}^{⊥}=\left(F+cI\right)-\left(V+cI\right)$$

*is another regular splitting, since F+cI≥0 remains nonnegative and V+cI remains a nonsingular M-matrix (raising the diagonal of an M-matrix preserves the M-matrix property). In epidemic models, a sparser, epidemiologically meaningful splitting into new-infection and removal rates is typically available directly, rather than obtained by shifting a given one.*

*A k–siphon model is an ME model with precisely k minimal siphons.*

*A k–strain simple model is a chemical ODE with precisely k disjoint minimal siphons $\sigma_{1},\ldots ,\sigma_{k}$.*

**Definition** **5** (*R*-invasion functions and rank-one *k*–strain models [RepFun])**.** *For a simple k-strain model, for each strain j and regular splitting, define next generation matrices $K_{j}\left(y_{j}\right)=\left(F_{j}V_{j}^{-1}\right)\left(y_{j}\right)$ and invasion functions*

$$R_{j}\left(y_{j}\right):=\rho \left(K_{j}\left(y_{j}\right)\right),$$

*$R_{j}\left(y_{j}\right)$ will be called an R-invasion function (associated with the block j).*

*A simple k-strain model is rank-one if every Jacobian transversal block $J_{\sigma }^{⊥}$ admits a regular splitting $J_{\sigma }^{⊥}=F_{\sigma }-V_{\sigma }$, such that the “new infections” matrix $F_{\sigma }$ has rank one. In this case, the R-invasion function and the Perron data admit explicit rank-one formulas.*

**Definition** **6** (reproduction numbers and invasibility numbers [Rnum])**.**

*1.*  *The evaluations*
  (1) $$R_{i}=R_{i}\left(E_{0}\right),i=1,\ldots ,k,$$
  *where $E_{0}$ is the DFE, and $R_{i}\left(y_{i}\right)$ are the invasion functions, will be called basic reproduction numbers (of strain i).*
*2.*  *In the case when each minimal siphon has a unique resident fixed point $E_{j},j=1,\ldots ,k,$*
  (2) $$R_{i}^{\tilde{j}}:=R_{i}\left(E_{j}\right)$$
  *will be called the invasibility number of invading block i on resident block j.*

**Definition** **7** (Simple and multiple boundary transcritical relays [BTR])**.** *A boundary transcritical relay $E\to \tilde{E}$ is a pair of equilibria such that the existence threshold of $\tilde{E}$ is one of the transversal instability thresholds of E. Multiplicity is counted by critical missing minimal-siphon blocks, never by unstable eigenvalues, since a vector block may contribute several eigenvalues while representing a single biological invasion direction: the relay out of E is called simple when exactly one missing minimal-siphon block is critical at E, and *multiple* when two or more are.*

**Definition** **8** (LAS exclusion partition [LASEP])**.** *A finite family of parameter regions is called a LAS exclusion partition if, away from its threshold hypersurfaces, each region contains exactly one locally asymptotically stable equilibrium support. This terminology does not require the stable support to contain only one competing block; coexistence supports are allowed.*

**Definition** **9** (Left and right invasion eigenvectors [inveig])**.** *Let E be a boundary equilibrium on the face of a missing minimal siphon Σ, let*

$$M_{\Sigma }\left(E\right):=J_{\Sigma }^{⊥}\left(E\right)$$

*be the corresponding transversal Jacobian block, which is Metzler, and assume it is irreducible. Its right and left invasion eigenvectors are the Perron vectors*

$$M_{\Sigma }\left(E\right)\;w_{\Sigma }=s\left(M_{\Sigma }\left(E\right)\right)\;w_{\Sigma },\;\pi_{\Sigma }^{⊤}M_{\Sigma }\left(E\right)=s\left(M_{\Sigma }\left(E\right)\right)\;\pi_{\Sigma }^{⊤},\;w_{\Sigma },\;\pi_{\Sigma }\gg 0,$$

*normalized by $\pi_{\Sigma }^{⊤}w_{\Sigma }=1$; the* invasion equation *is the threshold $s(M_{\Sigma }\left(E\right))=0$, equivalently $R_{\Sigma }\left(E\right)=1$ for the R-invasion function of Definition 5. The two vectors have complementary roles: $w_{\Sigma }$ is the escape direction along which the successor branch of the relay of Definition 7 leaves the face, and $\pi_{\Sigma }$ is the weight of the Perron functional $\pi_{\Sigma }^{⊤}z_{\Sigma }$ certifying noninvasion.*

**Remark** **5.** *A further motivation for unifying the CRN, ME and ecology viewpoints comes from the generalized Lotka–Volterra theory in ecology, which may be viewed as focusing on the particular case in which each species is a siphon, and whose equilibria satisfy a polynomial complementarity problem [18].*

*Solving Lotka–Volterra problems has a recursive structure: for each variable, either it is 0, and one is left with a system of dimension smaller by one, or it may be simplified. The same recursion holds for systems with several siphons, and it is the one implemented in EpidCRN.*

**Remark** **6** (The choice of Frobenius decomposition and splittings fixes the Perron data)**.** *Throughout, epidemic models (Definition 4) are considered with a fixed regular splitting*

$$J_{\sigma_{0}}^{⊥}\left(E_{0}\right)=F_{0}-V_{0},$$

*and the Frobenius decomposition and splittings are fixed once and for all, fixing the Perron data.*

For the reader’s convenience, we recall here, without new proof, two fundamental results of siphon calculus established elsewhere by the same authors: Theorem 1, from [1,11], and Theorem 2, from [12]. The first establishes the triangular block structure of the Jacobian on an invariant/siphon face (with the two blocks modeling, respectively, the tangential and transversal dynamics). This is precisely the nontrivial part of the NGM method [2,3,19,20], but the fact that the only and most natural hypothesis is forward invariance of the DFE face seems to be absent from the literature preceding [11,12].

**Theorem** **1** (triangular block form of the Jacobian on forward-invariant faces [tri])**.** *Let $x=\left(z,y\right)\in R_{\geq 0}^{m}\times R_{\geq 0}^{n}$ and let*

$$\dot{z}=f_{z}\left(z,y\right),\;\dot{y}=f_{y}\left(z,y\right)$$

*be a chemical vector field. Assume that the coordinate face*

$$F_{z}=\left\{z=0\right\}$$

*is forward invariant, equivalently, that the set of variables z is a siphon. Then:*

*1.*  *The mixed Jacobian block*
  $$J_{zy}\left(y\right)=D_{y}f_{z}\left(0,y\right)$$
  vanishes identically on $F_{z}$. Hence, at every point $\left(0,y^{*}\right)\in F_{z}$,
  $$J\left(0,y^{*}\right)=\left(\begin{array}{cc} J_{z} & 0\\ J_{yz} & J_{y} \end{array}\right),\;J_{z}=D_{z}f_{z}\left(0,y^{*}\right),\;J_{y}=D_{y}f_{y}\left(0,y^{*}\right).$$
*2.*  *The spectrum of the full Jacobian is*
  $$\sigma \;\left(J(0,y^{*})\right)=\sigma \left(J_{z}\right)\cup \sigma \left(J_{y}\right).$$
  Consequently, the fixed point $\left(0,y^{*}\right)$ is linearly asymptotically stable if and only if both $J_{z}$ and $J_{y}$ are Hurwitz. If either block has an eigenvalue with positive real part, then $\left(0,y^{*}\right)$ is unstable.

As a consequence, every stability problem on a siphon face factorizes into a tangential problem and a transversal problem. The tangential block depends only on the reduced dynamics on the face itself, whereas the transversal block measures invasion away from it. Theorem 1 reduces stability of $\left(0,y^{*}\right)$ to the Hurwitz property of the single transversal block $J_{z}$, provided the tangential block $J_{y}$ is already Hurwitz.

The purpose of the next theorem is to explain that Perron–Frobenius theory applies to the transversal Jacobian on every siphon face, due to its Metzler property. Cooperativity (a Metzler Jacobian) is usually a classical *hypothesis* in the theory of monotone dynamical systems; the novelty here is that it is not assumed but *derived*, from the chemical stoichiometric structure of Definition 2 alone.

**Theorem** **2** (Metzler property of the transversal Jacobian block on a siphon face [Met])**.** *Let*

$$\left(z,y\right)\in R_{\geq 0}^{m}\times R_{\geq 0}^{n}$$

*be the variables of a chemical ODE*

$$\dot{x}=\Gamma r\left(x\right),\;x=\left(z,y\right),$$

*in the sense of Definition 2. Assume that the set of z–variables is a siphon, so that the coordinate face*

$$F_{z}=\left\{z=0\right\}$$

*is forward invariant.*

*Then, at every point $\left(0,y\right)\in F_{z}$, the transversal Jacobian*

$$J_{z}^{⊥}\left(y\right)=D_{z}f_{z}\left(0,y\right)$$

*is Metzler:*

$$\frac{\partial f_{z_{i}}}{\partial z_{j}}\left(0,y\right)\geq 0,\;i\neq j.$$

*Equivalently, the linearized dynamics transversal to the siphon face,*

$$\dot{\xi }=J_{z}^{⊥}\left(y\right)\xi ,$$

*is cooperative.*

Once a splitting is fixed (Definition 4), Theorem 1’s Hurwitz condition on $J_{z}$ becomes the familiar NGM spectral-radius criterion:

**Corollary** **1.** *(Regular splitting and the NGM threshold)**.** In the setting of Theorem 1, suppose that*

$$J_{z}=F-V,$$

*where (F,V) is a regular splitting, so that*

$$F\geq 0,\;V^{-1}\geq 0,$$

*with V a nonsingular M-matrix. Then*

$$s\left(J_{z}\right)<0\;⟺\;\rho \left(FV^{-1}\right)<1,$$

$$s\left(J_{z}\right)=0\;⟺\;\rho \left(FV^{-1}\right)=1,$$

*and*

$$s\left(J_{z}\right)>0\;⟺\;\rho \left(FV^{-1}\right)>1.$$

Thus, if $J_{y}$ is Hurwitz, the fixed point $\left(0,y^{*}\right)$ of Theorem 1 is linearly asymptotically stable when $\rho (FV^{-1})<1$, and it is unstable when $\rho (FV^{-1})>1$.

**Definition** **10** (Siphon lattice and invariant faces [sip-lattice])**.**

- *L denotes the lattice generated by the minimal siphons.*
- *For Σ∈L,*
  $$F_{\Sigma }=\left\{x_{i}=0:\;i\in \Sigma \right\}$$
  *is the associated forward-invariant face.*
- *$E_{\Sigma }\in \mathrm{relint}\left(F_{\Sigma }\right)$ denotes the equilibrium on $F_{\Sigma }$ under consideration.*
- *At $E_{\Sigma }$, one has*
  $$x_{i}^{*}=0\;\mathrm{for}\;i\in \Sigma ,\;x_{i}^{*}>0\;\mathrm{for}\;i\notin \Sigma .$$

**Remark** **7** (Rank-one invasion numbers, for later use)**.** *For rank-one blocks with next-generation ratios $R_{k}=\pi_{k}^{⊤}V_{k}^{-1}w_{k}$ (Definition 5), the invasibility number of block k on a resident support I [21] is the quotient*

$$R_{k}\left(E_{I}\right)=\frac{R_{k}}{R_{I}},$$

*and the corresponding closing coefficient is the difference*

$$\frac{1}{R_{I}}-\frac{1}{R_{k}}=\frac{R_{k}\left(E_{I}\right)-1}{R_{k}}.$$

## 3. Generalized Lotka–Volterra Systems as Positive Reaction Systems

Throughout the paper, we consider generalized Lotka–Volterra systems of the form

(3) $$x_{i}^{\prime }=x_{i}f_{i}\left(x\right),\;i=1,\ldots ,n,$$

where each $f_{i}$ is a generalized polynomial

$$f_{i}\left(x\right)=\sum_{k=1}^{m_{i}}a_{ik}x^{\alpha_{ik}},$$

the exponents $\alpha_{ik}\in R^{n}$ being arbitrary real vectors. The state space is the positive orthant

$$R_{>0}^{n}=\left\{x\in R^{n}:x_{i}>0,\;i=1,\ldots ,n\right\}.$$

The multiplicative structure of (3) immediately places these systems inside the class of positive dynamical systems.

### 3.1. Coordinate Faces

For every subset

Σ⊆{1,…,n},

define the coordinate face

$$F_{\Sigma }=\left\{x\geq 0:x_{i}=0\Leftrightarrow i\in \Sigma \right\}.$$

Since every right-hand side contains the factor $x_{i}$, one has

$$x_{i}\left(0\right)=0\;⟹\;x_{i}\left(t\right)\equiv 0.$$

Consequently, every coordinate face is forward invariant. This elementary observation is the starting point of the entire siphon calculus.

**Proposition** **1** (Coordinate-face invariance is equivalent to the multiplicative GLV form)**.** *Every coordinate face $F_{\Sigma }$ is positively invariant under (3). Conversely, let $g:R_{>0}^{n}\to R^{n}$ be $C^{1}$ and extend $C^{1}$ to a neighborhood of $R_{\geq 0}^{n}$. If every coordinate hyperplane $\left\{x_{i}=0\right\}$ is positively invariant under $x^{\prime }=g\left(x\right)$, then g(x)=diag(x)f(x) for a (necessarily unique on $R_{>0}^{n}$) continuous f.*

*Coordinate-hyperplane invariance therefore characterizes the multiplicative* form* $x_{i}^{\prime }=x_{i}f_{i}\left(x\right)$, and nothing more: that the $f_{i}$ are generalized polynomials, as (3) requires, is an independent structural hypothesis which the converse does not supply.*

**Proof.**  (⇒) If $x_{i}=0$, then

$$x_{i}^{\prime }=x_{i}f_{i}\left(x\right)=0.$$

Hence, trajectories cannot leave the hyperplane $x_{i}=0$. Repeating the argument for every index belonging to Σ proves invariance of $F_{\Sigma }$.

(⇐) Fix *i*. Invariance of $\left\{x_{i}=0\right\}$ forces $g_{i}\left(x\right)=0$ whenever $x_{i}=0$: if $g_{i}\left(x^{0}\right)\neq 0$ for some $x^{0}$ with $x_{i}^{0}=0$, the vector field is not tangent to $\left\{x_{i}=0\right\}$ at $x^{0}$, so the trajectory through $x^{0}$ immediately leaves the hyperplane, contradicting invariance. Given $g_{i}\left(x\right)=0$ on $\left\{x_{i}=0\right\}$ and $g_{i}\in C^{1}$, define

$$f_{i}\left(x\right):=\int_{0}^{1}\partial_{i}g_{i}\left(x_{1},\ldots ,x_{i-1},sx_{i},x_{i+1},\ldots ,x_{n}\right)\;ds.$$

By the fundamental theorem of calculus applied along the segment from $\left(x_{1},\ldots ,0,\ldots ,x_{n}\right)$ to *x*,

$$g_{i}\left(x\right)=g_{i}\left(x\right)-g_{i}\left(x_{1},\ldots ,0,\ldots ,x_{n}\right)=x_{i}\int_{0}^{1}\partial_{i}g_{i}\left(x_{1},\ldots ,sx_{i},\ldots ,x_{n}\right)\;ds=x_{i}f_{i}\left(x\right),$$

using $g_{i}\left(x_{1},\ldots ,0,\ldots ,x_{n}\right)=0$. This is Hadamard’s lemma. Since $g_{i}\in C^{1}$, $f_{i}$ is continuous. Uniqueness on $R_{>0}^{n}$ is immediate: $f_{i}\left(x\right)=g_{i}\left(x\right)/x_{i}$ is forced by $x_{i}\neq 0$.    □

**Remark** **8** (Scope of boundary regularity)**.** *Proposition 1 applies to boundary-regular GLV systems, i.e., systems for which each $g_{i}\left(x\right)=x_{i}f_{i}\left(x\right)$ extends continuously to the relevant coordinate hyperplanes—as happens whenever every generalized-polynomial exponent is nonnegative, so that $g_{i}$ extends continuously to $R_{\geq 0}^{n}$.*

For GLV systems, the forward direction follows directly from the differential equations, since positivity is already built into their multiplicative structure.

### 3.2. Minimal Siphons

For arbitrary reaction systems, siphons are defined through the reaction graph (Definition 3): a set Σ of species is a *siphon* if every reaction producing a species of Σ also consumes one, so that $\left\{x_{i}=0,\;i\in \Sigma \right\}$ is forward invariant, and it is *minimal* if it contains no proper siphon. This is the notion used by Angeli, De Leenheer and Sontag [10] in the study of persistence, and the *semilocking sets* of Feinberg [22]; we use the two interchangeably and refer to those sources for the general theory, since only the invariance property is needed below: throughout this intrinsic scalar GLV theory, “siphon” therefore means any coordinate set whose vanishing defines a forward-invariant face of the GLV vector field, independently of which particular chemical realization is chosen—for GLV systems the combinatorial and invariance-based notions coincide by the cited equivalence, so no realization needs to be fixed to identify one. For generalized Lotka–Volterra systems the answer is considerably simpler: the minimal siphons are singletons:

**Corollary** **2** (The minimal siphons of a boundary-regular GLV system are its coordinates)**.** *Assume each $g_{i}\left(x\right)=x_{i}f_{i}\left(x\right)$ extends continuously to $R_{\geq 0}^{n}$, so that the coordinate hyperplanes lie in the domain. Then the minimal siphons are precisely the singleton coordinate sets*

{1},…,{n},

*
and every siphon is a union of them. The scope of the boundary-regularity hypothesis is discussed in Remark 8.*

**Proof.**  By Proposition 1,

$$x_{i}^{\prime }=x_{i}f_{i}\left(x\right),$$

so the hyperplane $x_{i}=0$ is invariant. Hence, every singleton is a siphon. Minimality is immediate because no nonempty proper subset of {i} exists.    □

Consequently, the relay graph developed below will contain only elementary coordinate invasions.

### 3.3. Boundary and Transversal Dynamics

Let

Σ⊆{1,…,n}.

On the invariant face

$$F_{\Sigma }$$

the surviving coordinates satisfy another generalized Lotka–Volterra system obtained simply by deleting the vanished variables.

Thus, every face carries a naturally induced subsystem. This recursive property is identical to the recursive construction used in general siphon calculus.

**Definition** **11** (resident siphon subsystems [res])**.** *The restriction of (3) to an invariant face $F_{\Sigma }$ is called the resident subsystem associated with* Σ*.*

The equilibrium structure may therefore be studied recursively over the face lattice. Interior equilibria of resident systems become boundary equilibria of the full system.

A siphon is inhabited precisely when its resident subsystem possesses a positive equilibrium in the relative interior of its face, as made precise below.

**Definition** **12** (Inhabited siphon)**.** *A siphon Σ⊊{1,…,n} is inhabited if its resident subsystem admits an equilibrium*

$$E_{\Sigma }\in F_{\Sigma },\;{\left(E_{\Sigma }\right)}_{i}>0\;\mathrm{for}\;\mathrm{every}\;i\notin \Sigma ,$$

*i.e., a fixed point of the resident subsystem with every surviving species strictly positive. A siphon for which no such $E_{\Sigma }$ exists is* uninhabited*: its face is invariant, but supports no genuine resident state, only the further boundary (ultimately the origin).*

For a minimal (singleton) siphon Σ={i}, inhabited means exactly that setting species *i* to zero leaves behind an (n−1)-species resident equilibrium with every remaining species positive; this is the weakest possible reading of “the boundary is dynamically nontrivial,” and is checked example by example in Section 9 below.

Suppose now

$$E_{\Sigma }\in F_{\Sigma }$$

is an equilibrium of the resident subsystem.

The transversal Jacobian associated with the missing coordinate i∈Σ is simply

$$\lambda_{i}\left(E_{\Sigma }\right)=f_{i}\left(E_{\Sigma }\right).$$

Indeed,

$$x_{i}^{\prime }=x_{i}f_{i}\left(x\right),$$

gives

$$\frac{\partial x_{i}^{\prime }}{\partial x_{i}}=f_{i}\left(x\right)+x_{i}\frac{\partial f_{i}}{\partial x_{i}},$$

and on the face $x_{i}=0$ the second term disappears. This is the scalar case of the block-triangular reduction on a forward-invariant face, Theorem 1: here every minimal siphon is a singleton, so each transversal block is 1 × 1 and its Metzler property (Theorem 2) is vacuous. The blocks become genuinely matrix-valued, and Theorem 2 load-bearing, already in this paper (Theorem 7, Corollary 20).

**Proposition** **2** (The transversal Jacobian on a GLV face is diagonal)**.** *Let $E_{\Sigma }$ be an equilibrium on $F_{\Sigma }$. Ordering the missing coordinates first, the Jacobian at $E_{\Sigma }$ is block triangular and its transversal block is*

$$J_{\Sigma }^{⊥}\left(E_{\Sigma }\right)=\mathrm{diag}\left(f_{i}\left(E_{\Sigma }\right):i\in \Sigma \right).$$

*In particular, the missing coordinates are transversally uncoupled: for scalar GLV systems, the Frobenius blocks of $J_{\Sigma }^{⊥}$ are one-dimensional. The transversal eigenvalue corresponding to the missing coordinate i equals*

$$\lambda_{i}\left(E_{\Sigma }\right)=f_{i}\left(E_{\Sigma }\right).$$

**Proof.**  Write $g_{i}\left(x\right)=x_{i}f_{i}\left(x\right)$. For every i,j,

$$\frac{\partial g_{i}}{\partial x_{j}}\left(x\right)=\delta_{ij}f_{i}\left(x\right)+x_{i}\frac{\partial f_{i}}{\partial x_{j}}\left(x\right).$$

At $E_{\Sigma }$ one has $x_{i}=0$ for every i∈Σ, so the second term vanishes on each such row. Row i∈Σ therefore has the single entry $f_{i}\left(E_{\Sigma }\right)$ on the diagonal and zeros elsewhere, which is simultaneously the block triangularity and the diagonality of $J_{\Sigma }^{⊥}\left(E_{\Sigma }\right)$. Consequently, the invasion exponent of a missing species is $\lambda_{i}\left(E_{\Sigma }\right)=f_{i}\left(E_{\Sigma }\right)$, and *i* invades exactly when $f_{i}\left(E_{\Sigma }\right)>0$.    □

This observation is elementary but fundamental. For scalar GLV systems, the invasion calculation reduces to the scalar quantities $f_{i}\left(E_{\Sigma }\right)$, and the previous proposition identifies the relay graph: its vertices are the resident equilibria, and there is an oriented edge

$$E_{\Sigma }⟶E_{\Sigma \setminus \{i\}}$$

whenever

$$f_{i}\left(E_{\Sigma }\right)>0.$$

Each edge corresponds to the successful invasion of one previously extinct species.

## 4. Volterra Lyapunov Functions for GLV Systems

This section develops the Lyapunov theory of a GLV vector field. Like Section 3, it depends only on the GLV vector field *f*, its positive equilibrium $x^{*}$, and, in the affine case, its interaction matrix *M*. Every criterion below is therefore a criterion on the ODE itself.

The object of study is the set of weights for which the Goh–Volterra function

$$L_{a}\left(x\right)=\sum_{i}a_{i}\;x_{i}^{*}\;G\left(x_{i}/x_{i}^{*}\right),\;G\left(u\right)=u-1-\log u,$$

decreases along every positive trajectory. Three questions organize the section: when does such a weight exist, can it be computed, and is there a canonical choice? For affine *f*, all three are answered exactly—by a matrix inequality (Theorem 3), by a semidefinite program (Theorem 3), and, in the irreducible Metzler case, by a closed-form Perron ratio (Theorem 4).

### 4.1. The Admissible-Weight Cone

We now make the object underlying every example below precise, so that the examples can be stated as instances of theorems rather than as isolated verifications.

**Definition** **13** (Admissible Volterra weights [cone])**.** Let $x^{\prime }=\mathrm{diag}\left(x\right)f\left(x\right)$ be a GLV system with equilibrium $x^{*}\in R_{>0}^{n}$, and for $a\in R_{>0}^{n}$ let

$${\dot{L}}_{a}\left(x\right):=\sum_{k=1}^{n}a_{k}\left(x_{k}-x_{k}^{*}\right)f_{k}\left(x\right)$$

be the derivative of $L_{a}\left(x\right)=\sum_{k}a_{k}x_{k}^{*}G\left(x_{k}/x_{k}^{*}\right)$ along the GLV system above. The equilibrium $x^{*}$ admits Volterra weights if

$$W\left(x^{*}\right):=\left\{a\in R_{>0}^{n}:{\dot{L}}_{a}\left(x\right)\leq 0\;\mathrm{for}\;\mathrm{all}\;x\in R_{>0}^{n}\right\}$$

is nonempty. $W(x^{*})$ is the admissible-weight cone at $x^{*}$.

**Lemma** **1** (Properness of the Volterra function, and its consequence for global stability)**.** For every $a\in R_{>0}^{n}$, $L_{a}$ is proper on $R_{>0}^{n}$: for every c≥0, the sublevel set $\left\{x\in R_{>0}^{n}:L_{a}\left(x\right)\leq c\right\}$ is compact. Consequently, whenever ${\dot{L}}_{a}\leq 0$ pointwise on $R_{>0}^{n}$ (in particular whenever $a\in W(x^{*})$, or under any hypothesis implying this), every positive trajectory is automatically precompact and exists for all t≥0: “positive trajectories are precompact” is not an extra hypothesis to be separately assumed in that case, but a consequence of ${\dot{L}}_{a}\leq 0$ together with properness.

**Proof.**  Since G(u)=u−1−logu≥0 is convex with G(u)→+∞ as $u\to 0^{+}$ or u→+∞, and each $a_{k}x_{k}^{*}>0$, every term $a_{k}x_{k}^{*}G\left(x_{k}/x_{k}^{*}\right)$ of $L_{a}$ is nonnegative and →+∞ as $x_{k}\to 0^{+}$ or $x_{k}\to +\infty$; since the other terms remain ≥0, $L_{a}\left(x\right)\to +\infty$ whenever any coordinate approaches the boundary of $R_{>0}^{n}$ or diverges. Hence, $\left\{x\in R_{>0}^{n}:L_{a}\left(x\right)\leq c\right\}$ is closed in $R_{>0}^{n}$, bounded away from $\partial R_{>0}^{n}$ and from infinity, and closed in $R^{n}$ (any boundary or infinite limit point would force $L_{a}\to \infty$, contradicting $L_{a}\leq c$ there), hence compact.

If ${\dot{L}}_{a}\leq 0$ pointwise, then along any solution x(t) with $x\left(0\right)=x_{0}$, $L_{a}\left(x\left(t\right)\right)\leq L_{a}\left(x_{0}\right)$ for as long as the solution exists, so x(t) remains in the compact set $K:=\{x\in R_{>0}^{n}:L_{a}\left(x\right)\leq L_{a}\left(x_{0}\right)\}$. By the standard continuation/escape theorem for ODEs on an open set (a maximal solution whose trajectory stays in a compact subset of the domain cannot have a finite maximal existence time, since it would otherwise have to leave every compact subset as *t* approaches that time), the solution exists for all t≥0, and its closure lies in *K*, hence is precompact.    □

**Lemma** **2** (The admissible-weight set is a convex cone)**.** $W(x^{*})$ is convex, and $a\in W(x^{*})$, $\lambda >0\Rightarrow \lambda a\in W(x^{*})$.

**Proof.**  For each fixed $x\in R_{>0}^{n}$, $a\mapsto {\dot{L}}_{a}\left(x\right)=\sum_{k}a_{k}\cdot c_{k}\left(x\right)$, $c_{k}\left(x\right):=\left(x_{k}-x_{k}^{*}\right)f_{k}\left(x\right)$, is linear in *a*; hence $H_{x}:=\left\{a\in R^{n}:{\dot{L}}_{a}\left(x\right)\leq 0\right\}$ is a closed half-space through the origin. Thus, $\left\{a:{\dot{L}}_{a}\left(x\right)\leq 0\;\forall x\right\}={⋂}_{x\in R_{>0}^{n}}H_{x}$ is a closed convex cone, and $W\left(x^{*}\right)=\left({⋂}_{x}H_{x}\right)\cap R_{>0}^{n}$ is the intersection of two convex sets, hence convex; it is closed under positive scaling because each $H_{x}$ is.    □

**Remark** **9.** Since $W(x^{*})$ is a cone, $W(x^{*})\neq ⌀$ iff $W\left(x^{*}\right)\cap \left\{a:\sum_{i}a_{i}=1\right\}\neq ⌀$: the existence question can always be normalized to the probability simplex without loss of generality, and any $a\in W(x^{*})$ may be rescaled to $a/\sum_{i}a_{i}$.

**Proposition** **3** (Transformation laws under diagonal rescaling)**.** Let $D=\mathrm{diag}(d_{1},\ldots ,d_{n})$, $d_{i}>0$, and write $D^{-1}W\left(x^{*}\right):=\left\{D^{-1}a:a\in W\left(x^{*}\right)\right\}$.

*(i)*  (Rescaling the vector field.) The system $x^{\prime }=D\mathrm{diag}\left(x\right)f\left(x\right)$ has the same equilibrium $x^{*}$, and its admissible-weight cone $W_{D}\left(x^{*}\right)$ satisfies $W_{D}\left(x^{*}\right)=D^{-1}W\left(x^{*}\right)$.
*(ii)*  (Rescaling the state.) With y=Dx one has $y^{\prime }=\mathrm{diag}\left(y\right)\tilde{f}\left(y\right)$, where $\tilde{f}\left(y\right):=f\left(D^{-1}y\right)$, a GLV system with equilibrium $y^{*}=Dx^{*}$, and $W\left(y^{*}\right)=D^{-1}W\left(x^{*}\right)$.

*The two rescalings are conceptually independent, yet induce the* same *action $a\mapsto D^{-1}a$ on admissible-weight cones.*

**Proof.**  (i) Writing ${\dot{V}}_{b}{|}_{D}$ for the derivative of $V_{b}$ along the *D*-rescaled flow, ${\dot{V}}_{b}{|}_{D}\left(x\right)=\sum_{k}b_{k}\left(x_{k}-x_{k}^{*}\right)\cdot d_{k}x_{k}f_{k}\left(x\right)/x_{k}=\sum_{k}\left(d_{k}b_{k}\right)\left(x_{k}-x_{k}^{*}\right)f_{k}\left(x\right)={\dot{V}}_{Db}\left(x\right)$ (derivative along the original flow). Hence, $b\in W_{D}\left(x^{*}\right)\Leftrightarrow Db\in W\left(x^{*}\right)\Leftrightarrow b\in D^{-1}W\left(x^{*}\right)$.

(ii) $y^{\prime }=Dx^{\prime }=D\mathrm{diag}\left(x\right)f\left(x\right)=\left(D\mathrm{diag}(D^{-1}y)\right)f\left(D^{-1}y\right)=\mathrm{diag}\left(y\right)f\left(D^{-1}y\right)$, using that $D\mathrm{diag}\left(D^{-1}y\right)=\mathrm{diag}\left(y\right)$ already (diagonal matrices multiply entrywise: $d_{k}\cdot {\left(D^{-1}y\right)}_{k}=d_{k}\cdot y_{k}/d_{k}=y_{k}$), so no further factor of *D* multiplies *f*. For $b\in R_{>0}^{n}$, writing $x=D^{-1}y$,

$${\dot{V}}_{b}\left(y\right)=\sum_{k}b_{k}\left(y_{k}-y_{k}^{*}\right){\tilde{f}}_{k}\left(y\right)=\sum_{k}b_{k}\cdot d_{k}\left(x_{k}-x_{k}^{*}\right)\cdot f_{k}\left(x\right)={\dot{V}}_{Db}\left(x\right),$$

using $y_{k}-y_{k}^{*}=d_{k}\left(x_{k}-x_{k}^{*}\right)$ and ${\tilde{f}}_{k}\left(y\right)=f_{k}\left(x\right)$. Since $y\in R_{>0}^{n}\Leftrightarrow x=D^{-1}y\in R_{>0}^{n}$, $b\in W\left(y^{*}\right)\Leftrightarrow Db\in W\left(x^{*}\right)\Leftrightarrow b\in D^{-1}W\left(x^{*}\right)$.

In (i) the field $f_{k}$ is itself scaled by $d_{k}$; in (ii) ${\tilde{f}}_{k}\left(y\right)=f_{k}\left(x\right)$ is unscaled but the shift $y_{k}-y_{k}^{*}=d_{k}\left(x_{k}-x_{k}^{*}\right)$ is scaled instead. Either way, exactly one factor $d_{k}$ enters the *k*th term of ${\dot{V}}_{b}$, which is why the two induced actions coincide.    □

**Remark** **10.** Proposition 3 shows that the group $\left(R_{>0}^{n},\times \right)$ of positive diagonal rescalings acts on GLV systems in two conceptually independent ways, with the same degree–(−1) action $a\mapsto D^{-1}a$ on admissible-weight cones. Rescaling time, t↦ct (c>0), is the special case D=cI of either action; since $W(x^{*})$ is already closed under positive scalar multiplication (Lemma 2), time rescaling leaves $W(x^{*})$ unchanged as a set. This answers, in the negative for time and affirmatively with an explicit exponent for vector-field and state rescaling, whether these transformations preserve admissibility: they do not preserve individual weight vectors, but they map W bijectively onto the corresponding cone of the transformed system, with the same exponent in both cases.

**Proposition** **4** (Diagonal stability is necessary for admissibility)**.** Let $x^{*}$ be an equilibrium and $J:=Df(x^{*})$ the Jacobian of f at $x^{*}$. If $a\in W(x^{*})$ then

$$\mathrm{diag}\left(a\right)J+J^{T}\mathrm{diag}\left(a\right)⪯0,$$

*i.e., a is a diagonal-stability certificate for J in the sense of classical Volterra–Lyapunov matrix stability theory.*

**Proof.**  Since $f(x^{*})=0$, $\partial_{i}{\dot{L}}_{a}\left(x^{*}\right)=a_{i}f_{i}\left(x^{*}\right)+\sum_{k}a_{k}\left(x_{k}-x_{k}^{*}\right)\partial_{i}f_{k}\left(x^{*}\right)=0$, so $x^{*}$ is a critical point of ${\dot{L}}_{a}$ for every a>0. Differentiating again and evaluating at $x^{*}$ (where every factor $x_{k}-x_{k}^{*}$ vanishes),

$$\partial_{j}\partial_{i}{\dot{L}}_{a}\left(x^{*}\right)=a_{i}\partial_{j}f_{i}\left(x^{*}\right)+a_{j}\partial_{i}f_{j}\left(x^{*}\right)=a_{i}J_{ij}+a_{j}J_{ji},$$

i.e., $\mathrm{Hess}\left({\dot{L}}_{a}\right)\left(x^{*}\right)=\mathrm{diag}\left(a\right)J+J^{T}\mathrm{diag}\left(a\right)$. If $a\in W(x^{*})$ then ${\dot{L}}_{a}\leq 0$ on a full neighborhood of $x^{*}$ in $R_{>0}^{n}$, so $x^{*}$ is a local maximum of ${\dot{L}}_{a}$, forcing its Hessian to be negative semidefinite.    □

**Remark** **11.** Proposition 4 is not merely sufficient-side bookkeeping: it explains Section 9.1’s negative finding. There, f is linear with J=M (independent of $x^{*}$), and the reported indefinite Hessians for a=(1,1,1) and $a=x^{*}$ (eigenvalues of one positive sign each) are exactly failures of this necessary condition—not artifacts of a bounded numerical search.

**Theorem** **3** (Affine weights: exact characterization and algorithm [class-F])**.** Suppose $f\left(x\right)=M\left(x-x^{*}\right)$ for a matrix $M\in R^{n\times n}$ (so $J=Df(x^{*})=M$ everywhere).

*(i)*  *(Characterization.)*
  $$W\left(x^{*}\right)=\left\{a\in R_{>0}^{n}:\mathrm{diag}\left(a\right)M+M^{T}\mathrm{diag}\left(a\right)⪯0\right\},$$
  *with equality, not merely the inclusion ⊆ of Proposition 4.*
*(ii)*  *(Algorithm.) Consider the semidefinite program*
  (4) $$\varepsilon^{*}=\max_{a,\varepsilon }\;\varepsilon \;\mathrm{subject}\;\mathrm{to}\;\sum_{i=1}^{n}a_{i}=1,\;a_{i}\geq \varepsilon \;\left(i=1,\ldots ,n\right),\;\mathrm{diag}\left(a\right)M+M^{T}\mathrm{diag}\left(a\right)⪯0.$$
  *Then, with the convention sup⌀=−∞ in the infeasible case,*
  $$W\left(x^{*}\right)\neq ⌀\Leftrightarrow \varepsilon^{*}>0.$$
  A solver reports infeasibility as a status distinct from a nonpositive optimal value, so both must be checked explicitly: the program can genuinely be infeasible rather than merely attain $\varepsilon^{*}\leq 0$ (e.g., M=I, where $\mathrm{diag}\left(a\right)M+M^{T}\mathrm{diag}\left(a\right)=2\mathrm{diag}\left(a\right)⪯0$ forces every $a_{i}\leq 0$, incompatible with $\sum_{i}a_{i}=1$). More precisely:
  *(a)*  if $\varepsilon^{*}>0$ and $a^{*}$ is an optimizer, then $a^{*}\in W\left(x^{*}\right)$ and $L_{a^{*}}\left(x\right)=\sum_{i}a_{i}^{*}x_{i}^{*}G\left(x_{i}/x_{i}^{*}\right)$ is a Volterra Lyapunov function;
  *(b)*  if the program is infeasible, or feasible with $\varepsilon^{*}\leq 0$, then $W(x^{*})=⌀$;
  *(c)*  every $a\in W(x^{*})$ is a positive multiple of a feasible point of (4) with ε>0.

*The existence and construction of affine Volterra weights is therefore an exact semidefinite feasibility problem.*

**Proof.**  (i) Here ${\dot{L}}_{a}\left(x\right)={\left(x-x^{*}\right)}^{T}\mathrm{diag}\left(a\right)M\left(x-x^{*}\right)$ exactly, a homogeneous quadratic form $Q_{a}\left(y\right)=y^{T}\mathrm{diag}\left(a\right)My$ in $y=x-x^{*}$, with no higher-order remainder. The domain $x\in R_{>0}^{n}$ corresponds to $y\in \{y>-x^{*}\}$, an open neighborhood of y=0 since $x^{*}>0$. If $Q_{a}\leq 0$ on this neighborhood, then for every $y_{0}\in R^{n}$ there is c>0 small enough that $cy_{0}\in \left\{y>-x^{*}\right\}$, whence $0\geq Q_{a}\left(cy_{0}\right)=c^{2}Q_{a}\left(y_{0}\right)$, so $Q_{a}\left(y_{0}\right)\leq 0$: negative semidefiniteness on a neighborhood of 0 implies negative semidefiniteness everywhere, by homogeneity. Hence ${\dot{L}}_{a}\leq 0$ on $R_{>0}^{n}$ iff $\mathrm{diag}\left(a\right)M+M^{T}\mathrm{diag}\left(a\right)⪯0$.

(ii) If $\hat{a}\in W\left(x^{*}\right)$, its normalization $a=\hat{a}/\sum_{j}{\hat{a}}_{j}$ satisfies $\sum_{i}a_{i}=1$, $a_{i}>0$, and (by homogeneity of the matrix inequality in *a*) $\mathrm{diag}\left(a\right)M+M^{T}\mathrm{diag}\left(a\right)⪯0$; with $\hat{\varepsilon }=\min_{i}a_{i}>0$, $\left(a,\hat{\varepsilon }\right)$ is feasible for (4), so in particular the program is feasible and $\varepsilon^{*}\geq \hat{\varepsilon }>0$. By contraposition, if (4) is infeasible, no such $\hat{a}$ can exist, so $W(x^{*})=⌀$ directly—this covers case (b)’s infeasible alternative. Conversely, at any optimizer $\left(a^{*},\varepsilon^{*}\right)$ with $\varepsilon^{*}>0$, the constraints $a_{i}^{*}\geq \varepsilon^{*}>0$ and $\mathrm{diag}\left(a^{*}\right)M+M^{T}\mathrm{diag}\left(a^{*}\right)⪯0$ say exactly that $a^{*}\in W\left(x^{*}\right)$; by contraposition, if the program is feasible with $\varepsilon^{*}\leq 0$, then $W(x^{*})=⌀$ too—case (b)’s other alternative. For (c): every $a\in W(x^{*})$ normalizes to a feasible point with $\varepsilon =\min_{i}a_{i}>0$ as above, and, since $W(x^{*})$ is a cone (Lemma 2), every positive multiple of a feasible *a* with ε>0 lies back in $W(x^{*})$.    □

**Corollary** **3** (Strict Volterra weights)**.** *There exists a≫0 with $\mathrm{diag}\left(a\right)M+M^{T}\mathrm{diag}\left(a\right)≺0$ if and only if*

$$\delta^{*}=\max_{a,\varepsilon ,\delta }\;\delta \;\mathrm{subject}\;\mathrm{to}\;\sum_{i}a_{i}=1,\;a_{i}\geq \varepsilon \geq 0,\;\mathrm{diag}\left(a\right)M+M^{T}\mathrm{diag}\left(a\right)⪯-2\delta I$$

admits a feasible point with ε>0,δ>0. For such an a, ${\dot{L}}_{a}\left(x\right)\leq -\delta {∥x-x^{*}∥}^{2}$ for every $x\in R_{>0}^{n}$.

**Proof.**  ${\dot{L}}_{a}\left(x\right)=y^{T}\mathrm{diag}\left(a\right)My=\frac{1}{2}y^{T}\left(\mathrm{diag}\left(a\right)M+M^{T}\mathrm{diag}\left(a\right)\right)y$ for $y=x-x^{*}$ (the affine identity underlying Theorem 3); the stated equivalence and decay bound follow directly from the added constraint ⪯−2δI.    □

**Corollary** **4** (The normalized admissible section is compact)**.** ${\bar{W}}_{1}:=\left\{a\in R_{\geq 0}^{n}:\sum_{i}a_{i}=1,\;\mathrm{diag}\left(a\right)M+M^{T}\mathrm{diag}\left(a\right)⪯0\right\}$ is compact and convex, and $W(x^{*})\neq ⌀$ iff ${\bar{W}}_{1}\cap R_{>0}^{n}\neq ⌀$.

**Proof.**  The simplex $\left\{a\geq 0:\sum_{i}a_{i}=1\right\}$ is compact and convex; the matrix inequality defines a closed convex set since its left side is affine in *a*. ${\bar{W}}_{1}$ is their intersection, hence compact and convex. The final equivalence is normalization of $W(x^{*})$ (one direction) and positive rescaling of a strictly positive point of ${\bar{W}}_{1}$ (the other).    □

**Remark** **12.** Theorem 3 supplies both directions of the weight-construction problem raised in Remark 15: $\varepsilon^{*}>0$ returns an admissible weight $a^{*}$, and $\varepsilon^{*}\leq 0$ certifies that none exists, with no gap between the two cases—there is no third outcome. By semidefinite duality, infeasibility (or $\varepsilon^{*}\leq 0$) may additionally be witnessed by an explicit dual separating certificate, whose precise form depends on the SDP convention used and is not needed for the statement above.

*Algorithm (affine Volterra weights). Given $M\in R^{n\times n}$:*

*1.*  *Introduce variables $a_{1},\ldots ,a_{n},\varepsilon$ and solve (4).*
*2.*  If $\varepsilon^{*}>0$, return $a^{*}$ and verify symbolically that $\mathrm{diag}\left(a^{*}\right)M+M^{T}\mathrm{diag}\left(a^{*}\right)⪯0$.
*3.*  If $\varepsilon^{*}\leq 0$, report that no Volterra function with strictly positive weights exists.

For exact rational M, exact arithmetic should be retained whenever the solver permits it; otherwise a numerical optimizer’s candidate weight must be post-processed by rational reconstruction where possible, an exact principal-minor or characteristic-polynomial check, or a rigorous eigenvalue bound if exact reconstruction is unavailable. A numerically small positive $\varepsilon^{*}$ is not, by itself, a proof.

**Corollary** **5** (Symmetric diagonal dominance produces admissible weights (in the spirit of Goh’s diagonal-dominance criterion), a constructive special case of Theorem 3)**.** Suppose f(x)=r+Ax with r>0, A Metzler ($a_{ii}<0$, $a_{ij}\geq 0$ for i≠j), and $x^{*}>0$ is an equilibrium ($r=-Ax^{*}$). If d>0 satisfies the symmetric diagonal-dominance condition

$$2d_{i}a_{ii}+\sum_{j\neq i}\left(d_{i}a_{ij}+d_{j}a_{ji}\right)\leq 0,\;i=1,\ldots ,n,$$

then $d\in W(x^{*})$; if every inequality is strict, ${\dot{L}}_{d}$ is strict away from $x^{*}$.

**Proof.**  Here $f\left(x\right)=A\left(x-x^{*}\right)$, so J=A and, by Theorem 3, $d\in W(x^{*})$ iff $S:=\mathrm{diag}\left(d\right)A+A^{T}\mathrm{diag}\left(d\right)⪯0$. *S* is symmetric with $S_{ii}=2d_{i}a_{ii}$ and, for i≠j, $S_{ij}=d_{i}a_{ij}+d_{j}a_{ji}\geq 0$ (since *A* is Metzler and d>0), so the stated hypothesis reads $S_{ii}+\sum_{j\neq i}\left|S_{ij}\right|\leq 0$ for every *i*: *S* is weakly diagonally dominant with nonpositive diagonal. By the Gershgorin circle theorem, every eigenvalue λ of *S* lies in some disc $|\lambda -S_{ii}|\leq \sum_{j\neq i}\left|S_{ij}\right|$, forcing $\lambda \leq S_{ii}+\sum_{j\neq i}\left|S_{ij}\right|\leq 0$; hence S⪯0. Strict inequality in every row makes every disc strictly inside the open left half-plane, giving S≺0. Section 9.2 verifies this condition directly (and, independently, via eigenvalues) on two instances.    □

**Remark** **13.** *A column-sum condition on A alone, $\sum_{i}d_{i}a_{ij}\leq 0$ for every j, is* not *sufficient for $\mathrm{diag}\left(d\right)A+A^{T}\mathrm{diag}\left(d\right)⪯0$: e.g., $A=(\begin{array}{cc} -10 & 0\\ 9 & -1 \end{array})$ is Metzler with d=(1,1) satisfying both column sums (−1,−1), yet $A+A^{T}=(\begin{array}{cc} -20 & 9\\ 9 & -2 \end{array})$ has determinant 40−81<0 and is indefinite. The row-sum condition on the symmetrized matrix S above, not a column-sum condition on A itself, is what Corollary 5 actually requires.*

**Remark** **14** (Three mechanisms for admissible weights)**.** The examples below instantiate exactly three ways an equilibrium can be shown to admit Volterra weights, in increasing order of difficulty: mechanism U ($1\in W(x^{*})$, verified directly: Section 9.3), mechanism DD ($W(x^{*})$ contains a diagonal-dominance vector, by Corollary 5: Section 9.2), and mechanism P ($W(x^{*})$ contains the canonical Perron/left–right kernel weight of Theorem 4, independently confirmed by the SDP feasibility problem of Theorem 3: Section 9.1). Mechanism U is the special case a=1 of the general SDP feasibility condition (Theorem 3) when $M+M^{T}$ (or, for nonlinear f, $J+J^{T}$ on a neighborhood of $x^{*}$ together with a global check) is already negative semidefinite without weighting.

**Remark** **15** (The weight-construction problem)**.** Theorem 3 turns “find the weights” into a precise computational problem for linear (and, locally via Proposition 4, for general) GLV systems: **given** M (or $J=Df(x^{*})$), decide whether there exists a>0 with $\mathrm{diag}\left(a\right)M+M^{T}\mathrm{diag}\left(a\right)⪯0$, and if so output a witness a. This is an instance of the classical diagonal stability feasibility problem for matrices: it is a semidefinite feasibility problem in the n unknowns $a_{1},\ldots ,a_{n}>0$, solvable in polynomial time by semidefinite programming; the 3×3 instance of Section 9.1 was solved directly via Sylvester’s criterion (three polynomial inequalities in a), which is an equivalent finite test avoiding SDP machinery in low dimension. Theorem 3 below makes this precise and complete: the normalized program (4) decides $W(x^{*})\neq ⌀$ exactly and returns a witness whenever one exists, so given/decide/output above is now a theorem, not a research problem, for every affine GLV system. Whether the optimizer of (4) is unique, whether $W(x^{*})$ can be characterized by a finite set of extreme rays for n≥3 (settled for n=2 by Theorem 5), and how the problem degrades for nonlinear f (where Proposition 4 is necessary but, unlike Theorem 3, not known to be sufficient) remain open.

**Theorem** **4** (Perron–Volterra theorem [Perron-Metzler-Volterra])**.** Let $f\left(x\right)=M\left(x-x^{*}\right)$, $x^{*}\in R_{>0}^{n}$, where M is an irreducible Metzler matrix, and let α:=s(M) be its spectral abscissa. By Perron–Frobenius (applied to M+cI for $c>-\min_{i}M_{ii}$, which is entrywise nonnegative and irreducible), α is a real, simple eigenvalue with strictly positive right and left eigenvectors w,π≫0, Mw=αw, $M^{T}\pi =\alpha \pi$, unique up to positive scaling. Set $a_{i}:=\pi_{i}/w_{i}$, A:=diag(a). Then:

*(i)*  if α≤0, $AM+M^{T}A⪯0$, hence $a\in W(x^{*})$;
*(ii)*  if α<0, $AM+M^{T}A≺0$: the Volterra function $L_{a}$ is strict, and, by Lemma 1, $x^{*}$ is globally asymptotically stable in $R_{>0}^{n}$;
*(iii)*  *if α=0, $\ker\left(AM+M^{T}A\right)=\mathrm{span}\left\{w\right\}$.*

More precisely, with W=diag(w), Π=diag(π), $z:=W^{-1}\left(x-x^{*}\right)$, and $c_{ij}:=\pi_{i}M_{ij}w_{j}+\pi_{j}M_{ji}w_{i}\geq 0$ (i≠j, nonnegative since M is Metzler and w,π≫0),

(5) $${\dot{L}}_{a}\left(x\right)=-\frac{1}{2}\sum_{1\leq i<j\leq n}c_{ij}{\left(z_{i}-z_{j}\right)}^{2}+\alpha \sum_{i=1}^{n}\pi_{i}w_{i}z_{i}^{2}.$$

**Proof.**  Let B:=ΠMW; $B_{ij}\geq 0$ for i≠j since *M* is Metzler. Using Mw=αw, $M^{T}\pi =\alpha \pi$: B1=ΠMw=αΠw, and $B^{T}1=WM^{T}\pi =\alpha W\pi$. Since Πw=Wπ (both equal the vector ${\left(\pi_{i}w_{i}\right)}_{i}$), $S:=B+B^{T}$ has row sums S1=2αΠw, i.e., $S_{ii}+\sum_{j\neq i}S_{ij}=2\alpha \pi_{i}w_{i}$ for every *i*. For any $z\in R^{n}$, expanding $z^{T}Sz=\sum_{i}S_{ii}z_{i}^{2}+\sum_{i\neq j}S_{ij}z_{i}z_{j}$ and using $\sum_{i<j}S_{ij}{\left(z_{i}-z_{j}\right)}^{2}=\sum_{i}z_{i}^{2}\sum_{j\neq i}S_{ij}-\sum_{i\neq j}S_{ij}z_{i}z_{j}$ (standard identity, since *S* is symmetric) gives $z^{T}Sz=\sum_{i}z_{i}^{2}\left(S_{ii}+\sum_{j\neq i}S_{ij}\right)-\sum_{i<j}S_{ij}{\left(z_{i}-z_{j}\right)}^{2}=2\alpha \sum_{i}\pi_{i}w_{i}z_{i}^{2}-\sum_{i<j}\left(B_{ij}+B_{ji}\right){\left(z_{i}-z_{j}\right)}^{2}$; dividing by 2 and writing $c_{ij}=B_{ij}+B_{ji}$ gives the displayed decomposition of $\frac{1}{2}z^{T}Sz$ (verified symbolically and numerically, including an end-to-end check against ${\dot{L}}_{a}$ computed directly). Since WA=Π, $W\left(AM+M^{T}A\right)W=\Pi MW+WM^{T}\Pi =B+B^{T}=S$; as *W* is invertible, this congruence carries the sign properties of *S* to $AM+M^{T}A$ (for p≠0, setting $u=W^{-1}p$, $p^{T}\left(AM+M^{T}A\right)p=u^{T}Su$). With $y=x-x^{*}=Wz$, ${\dot{L}}_{a}\left(x\right)=y^{T}AMy=\frac{1}{2}y^{T}\left(AM+M^{T}A\right)y=\frac{1}{2}z^{T}Sz$, proving (5).

(i) If α≤0, both terms of (5) are ≤0 (the edge term since $c_{ij}\geq 0$; the diagonal term since α≤0 and $\pi_{i}w_{i}>0$), so ${\dot{L}}_{a}\leq 0$, i.e., S⪯0, hence $AM+M^{T}A⪯0$. (ii) If α<0, the diagonal term is <0 for z≠0 (some $\pi_{i}w_{i}z_{i}^{2}>0$) while the edge term is ≤0; the sum is <0 for every z≠0, so S≺0 and $AM+M^{T}A≺0$. Global asymptotic stability follows from Lemma 1 and LaSalle’s invariance principle, exactly as in Corollary 11.

(iii) If α=0, ${\dot{L}}_{a}\left(x\right)=0$ forces $c_{ij}{\left(z_{i}-z_{j}\right)}^{2}=0$ for every pair, i.e., $z_{i}=z_{j}$ whenever $c_{ij}>0$. Since *M* is irreducible, $M_{ij}>0$ or $M_{ji}>0$ for every edge of its (strongly connected) associated graph, so $c_{ij}>0$ there too, making the undirected graph $\left\{\left(i,j\right):c_{ij}>0\right\}$ connected; hence $z_{1}=\ldots =z_{n}$, i.e., z∈span{1}, i.e., y=Wz∈span{w}. Since S⪯0 by (i), $\{y:y^{T}Sy=0\}=\ker S$ (standard fact for negative-semidefinite quadratic forms), giving $\ker\left(AM+M^{T}A\right)=\mathrm{span}\left\{w\right\}$.    □

**Corollary** **6** (Exact Metzler criterion)**.** *Let M be irreducible and Metzler. There exists a diagonal A≫0 with $AM+M^{T}A⪯0$ iff s(M)≤0; there exists diagonal A≫0 with $AM+M^{T}A≺0$ iff s(M)<0.*

**Proof.**  Sufficiency is Theorem 4. Conversely, if $AM+M^{T}A⪯0$ for some diagonal A≫0 and w≫0 is the right Perron vector (Mw=s(M)w), then $w^{T}\left(AM+M^{T}A\right)w=2w^{T}AMw=2s\left(M\right)w^{T}Aw\leq 0$ (using $w^{T}M^{T}Aw=w^{T}AMw$, a scalar identity for any matrix), and since $w^{T}Aw=\sum_{i}a_{i}w_{i}^{2}>0$, s(M)≤0. The strict case is identical with strict inequalities throughout.    □

**Corollary** **7** (The admissible cone is automatically nonempty in the stable Metzler sector)**.** For $f\left(x\right)=M\left(x-x^{*}\right)$ with M irreducible Metzler, $W\left(x^{*}\right)\neq ⌀\Leftrightarrow s\left(M\right)\leq 0$, with canonical witness $a_{i}=\pi_{i}/w_{i}$ for the Perron eigenvectors w,π of M.

**Proposition** **5** (Exact criterion for triangular systems)**.** Let $M\in R^{n\times n}$ be (upper or lower) triangular, with no sign hypothesis on the off-diagonal entries. Then there exists a diagonal A≫0 with $AM+M^{T}A≺0$ if and only if M is Hurwitz, i.e., iff every diagonal entry of M is negative. Equivalently, for $f\left(x\right)=M\left(x-x^{*}\right)$, a strict Volterra Lyapunov function exists if and only if $x^{*}$ is locally asymptotically stable.

**Proof.**  Necessity is immediate: diagonal stability implies Hurwitz stability in general (Proposition 4, or directly since $AM+M^{T}A≺0\Rightarrow M$ has no eigenvalue with nonnegative real part). For sufficiency, assume without loss of generality *M* is lower triangular with $M_{ii}<0$ for every *i* (its eigenvalues, for a triangular matrix, are exactly its diagonal entries), and construct $a=(a_{1},\ldots ,a_{n})$ inductively. For n=1, any $a_{1}>0$ works since $2a_{1}M_{11}<0$. For the inductive step, suppose $a_{1},\ldots ,a_{k-1}>0$ have been chosen so that the leading (k−1)×(k−1) block $S^{\prime }:=\mathrm{diag}\left(a_{1},\ldots ,a_{k-1}\right)M^{\prime }+M^{\prime T}\mathrm{diag}\left(a_{1},\ldots ,a_{k-1}\right)≺0$, where $M^{\prime }$ is *M*’s leading (k−1)×(k−1) block. Write row *k* of *M* as $\left(r^{T},M_{kk}\right)$, $r\in R^{k-1}$. For $a_{k}>0$, the leading k×k block of $\mathrm{diag}\left(a_{1},\ldots ,a_{k}\right)M+M^{T}\mathrm{diag}\left(a_{1},\ldots ,a_{k}\right)$ is the bordered matrix $\left(\begin{array}{cc} S^{\prime } & a_{k}r\\ a_{k}r^{T} & 2a_{k}M_{kk} \end{array}\right)$, negative definite (by the Schur complement criterion, since $S^{\prime }≺0$ already) iff $2a_{k}M_{kk}-a_{k}^{2}r^{T}{\left(S^{\prime }\right)}^{-1}r<0$. Since $S^{\prime }≺0$, ${\left(S^{\prime }\right)}^{-1}≺0$, so $c:=-r^{T}{\left(S^{\prime }\right)}^{-1}r\geq 0$; the condition becomes $a_{k}\left(2M_{kk}+a_{k}c\right)<0$, which holds for c=0 at every $a_{k}>0$, and for c>0 at every $0<a_{k}<-2M_{kk}/c$ (a nonempty interval, since $M_{kk}<0$). Either way a valid $a_{k}>0$ exists, completing the induction (verified exactly by this construction on random 4×4 and 5×5 Hurwitz triangular instances: the resulting $S=\mathrm{diag}\left(a\right)M+M^{T}\mathrm{diag}\left(a\right)$ has all eigenvalues negative).    □

**Remark** **16.** *Proposition 5 is the second complete “if and only if” characterization in this paper, alongside the Metzler case of Corollary 6, and the n=2 case of Theorem 5 (every 2×2 admissible cone is computed exactly, hence its nonemptiness is also an iff). It requires no sign pattern at all on M’s off-diagonal entries—unlike the Metzler case, cooperative and competitive triangular systems are treated uniformly. What remains open, precisely, is the fourth case of Section 9.4’s classification: an iff criterion for general graph-generated, complex-balanced systems, without a triangular or Metzler sign hypothesis on M.*

**Definition** **14** (Perron–Volterra weight [PVweight])**.** *Let M be an irreducible Metzler with s(M)≤0. The vector $a_{i}^{P}:=\pi_{i}/w_{i}$, where w,π≫0 are the right and left Perron vectors of M, is the* Perron–Volterra weight of M. Scaling w,π independently changes $a^{P}$ only by one common positive scalar (since Perron vectors are each unique up to scale), so its ray is canonical—but not the only admissible ray: the cone $W(x^{*})$ may contain others (Remark 38 exhibits two distinct ones for the same matrix).

**Remark** **17** (The conservative and Hurwitz theorems, and the two affine forms)**.** *Theorem 4 subsumes Corollaries 8 and 11, and identifies the exact spectral threshold between them:*

$$\begin{array}{ccc} \mathrm{matrix}\;\mathrm{regime} & \mathrm{canonical}\;\mathrm{vectors} & \mathrm{conclusion}\\ s(M)=0 & Mw=0,\;M^{T}\pi =0 & AM+M^{T}A⪯0\\ s(M)<0 & Mw=s\left(M\right)w,\;M^{T}\pi =s\left(M\right)\pi & AM+M^{T}A≺0\\ \mathrm{general}\;\mathrm{subeigenvectors} & Mw\leq 0,\;M^{T}\pi \leq 0 & \mathrm{admissible},\;\mathrm{possibly}\;\mathrm{noncanonical} \end{array}$$

Corollary 8 is the case α=s(M)=0: $x^{\prime }=\mathrm{diag}\left(x\right)Mx$ and $x^{\prime }=\mathrm{diag}\left(x\right)M\left(x-x^{*}\right)$ coincide exactly when $Mx^{*}=0$, and then $x^{*}\gg 0$ is, by Perron–Frobenius uniqueness of the strictly positive eigenvector, the right Perron vector for eigenvalue 0, so $v=x^{*}$ recovers $a_{i}=\pi_{i}/x_{i}^{*}$ directly (a general left kernel vector ℓ=π, not necessarily the left Perron vector—Corollary 8 only required $\ell^{T}M=0$, which for a simple eigenvalue 0 forces ℓ‖w automatically). Corollary 11 is the case α=s(M)<0 with w,π arbitrary subeigenvectors (Mw≪0, $M^{T}\pi \ll 0$, not necessarily eigenvectors): it gives a larger family of admissible certificates, of which the Perron weight is the canonical member. These are genuinely different vectors in general, not merely different derivations of the same one—e.g., on the cooperative family’s asymmetric instance (Remark 38), the Perron weight and the inverse-formula weight $-M^{-T}1/\left(-M^{-1}1\right)$ of Corollary 12 are close but not proportional. Corollary 11 is therefore retained as a genuinely more general (if less canonical) construction, not merely restated by the Perron theorem. Both affine forms above concern diagonal stability of the same matrix M: whenever $Mx^{*}=0$ (the conservative case), the theorem applies to either representation of f interchangeably; when $Mx^{*}\neq 0$ (the strict affine case, where M is only the linear part Df, not required to annihilate $x^{*}$), only the shifted form $f\left(x\right)=M\left(x-x^{*}\right)$ is meaningful, and $Mx^{*}=0$ must not *be imposed.*

**Corollary** **8** (Canonical weights, conservative Metzler case)**.** *Consider*

(6) $$x^{\prime }=\mathrm{diag}\left(x\right)Mx,\;x\in R_{>0}^{n},$$

where M is an irreducible Metzler matrix (off-diagonal entries ≥0). Suppose there exist $x^{*}\gg 0$, ℓ≫0 with $Mx^{*}=0$ and $\ell^{T}M=0$, and set $a_{i}:=\ell_{i}/x_{i}^{*}$, A:=diag(a). Then $AM+M^{T}A⪯0$, hence $a\in W(x^{*})$ and $L_{a}\left(x\right)=\sum_{i}\ell_{i}G\left(x_{i}/x_{i}^{*}\right)$ is a Volterra Lyapunov function. More precisely, with $z_{i}:=\left(x_{i}-x_{i}^{*}\right)/x_{i}^{*}$ and $b_{ij}:=\ell_{i}M_{ij}x_{j}^{*}$,

$${\dot{L}}_{a}\left(x\right)=-\frac{1}{2}\sum_{1\leq i<j\leq n}\left(b_{ij}+b_{ji}\right){\left(z_{i}-z_{j}\right)}^{2}\leq 0,$$

with equality, by irreducibility of M, exactly when $z_{1}=\cdots =z_{n}$, i.e., $x=cx^{*}$ for some c>0.

**Proof.**  Let $X=\mathrm{diag}(x^{*})$, L=diag(ℓ), B:=LMX. Since *M* is Metzler and $\ell ,x^{*}\gg 0$, $B_{ij}\geq 0$ for i≠j; and $B1=LMx^{*}=0$, $1^{T}B=\ell^{T}MX=0$, so *B* has zero row and column sums. The symmetric matrix $B+B^{T}$ then has nonnegative off-diagonal entries and zero row sums, so for every $z\in R^{n}$ (the standard graph-Laplacian identity, verified by direct expansion)

(7) $$z^{T}\left(B+B^{T}\right)z=-\sum_{1\leq i<j\leq n}\left(B_{ij}+B_{ji}\right){\left(z_{i}-z_{j}\right)}^{2}\leq 0.$$

Since XA=L (X,A diagonal), $X\left(AM+M^{T}A\right)X=LMX+XM^{T}L=B+B^{T}$; as *X* is invertible, this congruence and (7) give $AM+M^{T}A⪯0$ (for any *v*, setting u=Xv, $v^{T}\left(AM+M^{T}A\right)v=u^{T}\left(B+B^{T}\right)u\leq 0$, using $X^{T}=X$). For the dissipation identity: with $y=x-x^{*}$, $z=X^{-1}y$, and $Mx^{*}=0$ so Mx=My, ${\dot{L}}_{a}\left(x\right)=\sum_{i}a_{i}\left(x_{i}-x_{i}^{*}\right)f_{i}\left(x\right)=y^{T}AMy=\frac{1}{2}y^{T}\left(AM+M^{T}A\right)y=\frac{1}{2}{\left(Xz\right)}^{T}\left(AM+M^{T}A\right)\left(Xz\right)=\frac{1}{2}z^{T}\left(B+B^{T}\right)z$, which is the displayed formula by (7). Irreducibility of *M* makes the undirected graph on edges $\left\{\left(i,j\right):B_{ij}+B_{ji}>0\right\}$ connected, so equality forces $z_{i}=z_{j}$ along every edge, hence $z_{1}=\cdots =z_{n}$.    □

**Corollary** **9** (Column-conservative case)**.** Under Corollary 8, if $1^{T}M=0$ (column sums zero), one may take ℓ=1, giving the canonical weight $a_{i}=1/x_{i}^{*}$ and Volterra function $V\left(x\right)=\sum_{i}G\left(x_{i}/x_{i}^{*}\right)$.

**Proof.**  ℓ=1 satisfies $\ell^{T}M=1^{T}M=0$, the left-kernel hypothesis of Corollary 8.    □

**Remark** **18** (The construction extends beyond detailed balance)**.** The construction uses the weaker condition $AM+M^{T}A⪯0$, in place of $AM=M^{T}A$: only the symmetric part $B+B^{T}$ enters the dissipation identity, and the antisymmetric circulation drops out of $z^{T}\left(B+B^{T}\right)z$ entirely, whatever the symmetry of LMX itself. The weights $a_{i}=\ell_{i}/x_{i}^{*}$ are therefore canonical left–right kernel weights; they earn the name “reversibilizing measure” only on the further, separately verified, hypothesis of diagonal symmetrizability.

**Corollary** **10** (Strictness on conserved affine classes)**.** Under Corollary 8, suppose in addition that $1^{T}x$ is conserved along trajectories of (6). Then every affine class $C_{c}=\left\{x\in R_{>0}^{n}:1^{T}x=c\right\}$ contains at most one point of the equilibrium ray $\left\{rx^{*}:r>0\right\}$, and $L_{a}$ is strict on each $C_{c}$ away from its unique equilibrium.

**Proof.**  If $rx^{*},sx^{*}\in C_{c}$, then $r1^{T}x^{*}=c=s1^{T}x^{*}$, forcing r=s; the conclusion follows from the equality case of Corollary 8.    □

**Remark** **19** (Linear conservation is a more restrictive subclass)**.** Conservation of $1^{T}x$ requires $\dot{\left(1^{T}x\right)}=x^{T}Mx\equiv 0$, i.e., M itself skew-symmetric—strictly stronger than the conservative kernel condition $Mx^{*}=0$, $\ell^{T}M=0$ alone. The cyclic triangle instead possesses log-linear conserved quantities: on the tested instance, $x^{T}Mx=-3x_{1}^{2}-4x_{2}^{2}-5x_{3}^{2}+3x_{1}x_{2}+5x_{1}x_{3}+4x_{2}x_{3}$ (verified symbolically) is a genuinely nonzero quadratic form, so $1^{T}x$ varies there, while the triangle’s actual conserved quantities are the compatibility manifolds of Section 8—log-linear rather than linear in x, meeting the equilibrium set in exactly one point, and available once the Euclidean-graph realization of Part II has been fixed. Corollary 10 therefore covers a genuinely different, more restrictive class of conservative Metzler systems, such as ordinary linear conversion networks, which lack the multiplicative GLV factor $x_{i}$ that makes $x^{T}Mx$ a nontrivial quadratic form here.

**Corollary** **11** (Canonical strict weights, Hurwitz Metzler case)**.** Consider $f\left(x\right)=M\left(x-x^{*}\right)$, $x^{*}\in R_{>0}^{n}$, where M is Metzler (off-diagonal entries ≥0) and Hurwitz (every eigenvalue has negative real part). Suppose there exist w,π≫0 with Mw≪0 and $M^{T}\pi \ll 0$ (componentwise strict), and set $a_{i}:=\pi_{i}/w_{i}$, A:=diag(a). Then $AM+M^{T}A≺0$, hence $a\in W(x^{*})$ and $L_{a}\left(x\right)=\sum_{i}a_{i}x_{i}^{*}G\left(x_{i}/x_{i}^{*}\right)$ is a strict Volterra Lyapunov function: there exists δ>0 with ${\dot{L}}_{a}\left(x\right)\leq -\delta {∥x-x^{*}∥}^{2}$ for every $x\in R_{>0}^{n}$. Consequently, by Lemma 1, $x^{*}$ is globally asymptotically stable in $R_{>0}^{n}$.

**Proof.**  Let V=diag(v), W=diag(w), B:=ΠMW; since *M* is Metzler and w,π≫0, $B_{ij}\geq 0$ for i≠j. Since Mw≪0 and π≫0, B1=ΠMw≪0; since $M^{T}\pi \ll 0$ and w≫0, $B^{T}1=WM^{T}\pi \ll 0$. Hence, $S:=B+B^{T}$ is symmetric, has nonnegative off-diagonal entries, and $S1=\Pi Mw+WM^{T}\pi \ll 0$, i.e., for every *i*, $S_{ii}+\sum_{j\neq i}S_{ij}<0$: *S* is symmetric, row-diagonally dominant with strictly negative row sums and nonnegative off-diagonal entries, so (Gershgorin’s theorem: every eigenvalue lies in a disc centered at $S_{ii}$ of radius $\sum_{j\neq i}\left|S_{ij}\right|=\sum_{j\neq i}S_{ij}$, and $S_{ii}+\sum_{j\neq i}S_{ij}<0$ places every disc strictly left of 0) S≺0. As VA=W (V,A diagonal), $W\left(AM+M^{T}A\right)W=\Pi MW+WM^{T}\Pi =B+B^{T}=S≺0$; since *W* is invertible, this congruence gives $AM+M^{T}A≺0$: for p≠0, set $u=V^{-1}p\neq 0$, so $p^{T}\left(AM+M^{T}A\right)p=u^{T}V\left(AM+M^{T}A\right)Vu=u^{T}Su<0$, and this holds for every p≠0 since *V* is bijective on $R^{n}\setminus \left\{0\right\}$. With $y=x-x^{*}$, ${\dot{L}}_{a}\left(x\right)=y^{T}AMy=\frac{1}{2}y^{T}\left(AM+M^{T}A\right)y$ (the affine identity of Theorem 3); negative definiteness of $AM+M^{T}A$ gives $\delta :=-\frac{1}{2}\lambda_{\max}\left(AM+M^{T}A\right)>0$ with ${\dot{L}}_{a}\left(x\right)\leq -\delta {∥y∥}^{2}$. The global conclusion follows from Lemma 1 and LaSalle’s invariance principle.    □

**Corollary** **12** (An explicit inverse formula)**.** *Under Corollary 11, one may take $v=-M^{-1}1$, $w=-M^{-T}1$: since M is Hurwitz and Metzler, −M is a nonsingular M-matrix, so $-M^{-1}\geq 0$ entrywise (and $-M^{-1}\gg 0$ if M is additionally irreducible, a classical Perron–Frobenius fact for irreducible M-matrices), giving w,π≫0 with Mw=−1≪0, $M^{T}\pi =-1\ll 0$ exactly. The resulting weight $a_{i}={\left(-M^{-T}1\right)}_{i}/{\left(-M^{-1}1\right)}_{i}$ is strictly admissible. (If M is reducible, strictly positive w,π satisfying Mw≪0, $M^{T}\pi \ll 0$ can still be found—e.g., by the standard characterization of Hurwitz Metzler matrices—but need not equal $-M^{-1}1$, $-M^{-T}1$ exactly.)*

**Proof.**  Immediate from $M(-M^{-1}1)=-1$, $M^{T}\left(-M^{-T}1\right)=-1$, and Corollary 11.    □

**Definition** **15** (Metzler subeigenvector weight [Met])**.** *Let M be Metzler. A positive vector a is a Metzler subeigenvector weight for M if there exist w,π≫0 with Mw≤0, $M^{T}\pi \leq 0$, and $a_{i}=\pi_{i}/w_{i}$; it is* strict *if both inequalities are strict.*

**Remark** **20** (Critical and strictly stable Metzler cases)**.** *Corollary 8 and Corollary 11 are the critical (Mw=0, $M^{T}\pi =0$) and strict (Mw≪0, $M^{T}\pi \ll 0$) cases of the same mechanism:*

$$\begin{array}{c} a\;\mathrm{Metzler}\;\mathrm{subeigenvector}\;\mathrm{weight}\;(\mathrm{Definition}\;15\;)\;\mathrm{is}\;\mathrm{automatically}\;\mathrm{an}\;\mathrm{admissible}\;\mathrm{Volterra}\;\mathrm{weight}, \end{array}$$

*negative semidefinite in the critical case (equality sets the equilibrium ray) and negative definite in the strict case. Neither case is unique: different choices of w,π can give different Metzler subeigenvector weights for the same M (Remark 38 exhibits two genuinely different ones for the same matrix), and neither construction requires detailed balance ($AM=M^{T}A$)—only the weaker inequality $AM+M^{T}A⪯0$ or ≺0, exactly as in Remark 18.*

**Remark** **21** (Structural solution of the SDP in the Metzler sector)**.** For a Hurwitz Metzler M, the affine semidefinite feasibility problem (4) is automatically feasible, with the explicit feasible point $a_{i}={\left(-M^{-T}1\right)}_{i}/{\left(-M^{-1}1\right)}_{i}$ of Corollary 12 (or, if M is reducible, the more general $\pi_{i}/w_{i}$ of Corollary 11). In the Metzler sector the SDP therefore remains useful for optimization within $W(x^{*})$ or for describing the full admissible cone, but it is not needed merely to decide $W(x^{*})\neq ⌀$.

#### Running the Weight SDP on Two Instances

Theorem 3 is stated as an algorithm, so it can be run. We run it on two instances: the Metzler instance of Section 9.2, where Remark 21 predicts that the semidefinite program has nothing to decide, and a non-Metzler instance where it has everything to decide. Both are exactly reproducible; the code is the five lines listed at the end of this subsection.

**Instance 1: the cooperative matrix of Section 9.2.** Take the asymmetric Goh instance r=(2,1,3),

$$M=\left(\begin{array}{ccc} -4 & 1 & 2\\ 2 & -5 & 1\\ 1 & 3 & -6 \end{array}\right),$$

for which Section 9.2 exhibits the diagonal-dominance weight $d=(1,\frac{4}{9},\frac{11}{27})$ of Corollary 5. Program (4) reads

$$\max_{a\in R^{3},\varepsilon \in R}\;\varepsilon \;s.t.\;a_{1}+a_{2}+a_{3}=1,\;a_{i}\geq \varepsilon ,\;\mathrm{diag}\left(a\right)M+M^{T}\mathrm{diag}\left(a\right)⪯0,$$

and its value is

$$\varepsilon^{*}=\frac{1}{3},\;a^{*}=\left(\frac{1}{3},\frac{1}{3},\frac{1}{3}\right),\;\mathrm{spec}\left(\mathrm{diag}\left(a^{*}\right)M+M^{T}\mathrm{diag}\left(a^{*}\right)\right)\approx \left\{-5.0588,\;-3.8810,\;-1.0602\right\}.$$

This value is exact, and provable without a solver: $\min_{i}a_{i}\leq 1/n$ on the simplex, with equality only at the barycenter, so $\varepsilon^{*}\leq \frac{1}{3}$ always, with equality iff the barycenter is feasible, i.e., iff $M+M^{T}⪯0$. Here $M+M^{T}=\left(\begin{array}{ccc} -8 & 3 & 3\\ 3 & -10 & 4\\ 3 & 4 & -12 \end{array}\right)$ has leading principal minors −8, 71, −562, alternating in sign, so $M+M^{T}≺0$ by Sylvester’s criterion in exact integer arithmetic. The optimizer is therefore the uniform weight, and the certificate returned by the solver is a numerical restatement of an integer determinant computation.

**Figure 1:**
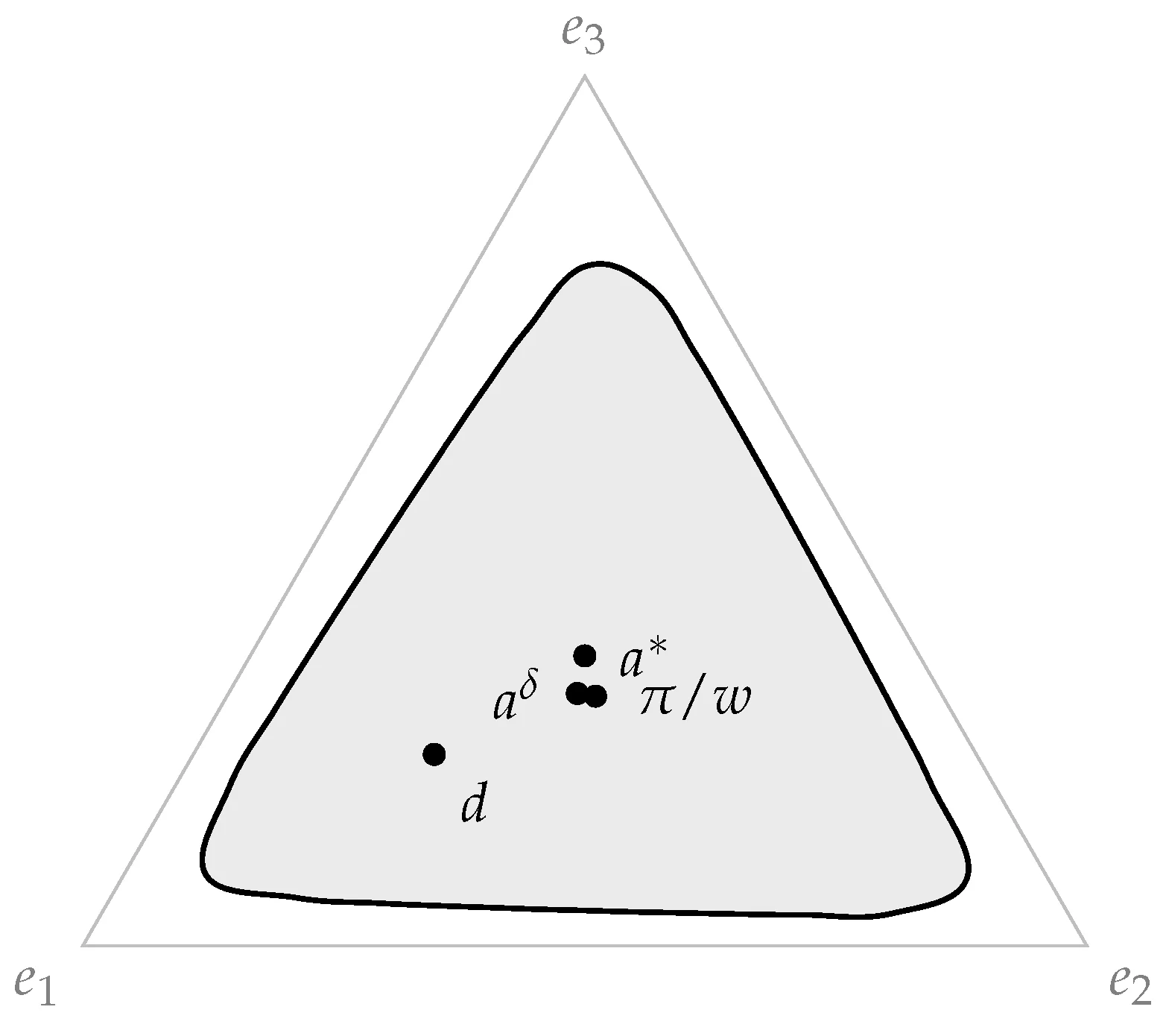
The admissible-weight cone of Instance 1, sliced by $a_{1}+a_{2}+a_{3}=1$ and drawn in barycentric coordinates on the simplex with vertices $e_{1},e_{2},e_{3}$. The shaded region is ${\bar{W}}_{1}$ of Corollary 4; it occupies about 68% of the simplex, its boundary is the cubic $\det(\mathrm{diag}\left(a\right)M+M^{T}\mathrm{diag}\left(a\right))=0$, and it meets no face $a_{i}=0$ (the smallest coordinate anywhere on the boundary is 0.0354), so every admissible weight is strictly positive by a definite margin. Marked: the SDP optimizer $a^{*}$ (the barycenter), Goh’s diagonal-dominance weight *d*, the Perron weight π/w, and the maximal-margin weight $a^{\delta }$.

The biological content of the certificate is that this cooperative community needs no weighting at all: the unweighted Volterra function $L_{1}$ already decreases, so the instance is mechanism U of Remark 14, not merely mechanism DD. Goh’s [5] weight *d*, normalized to (0.54,0.24,0.22), is one admissible point among many ($\lambda_{\max}=-0.8452$ there), as are the Perron weights $\pi_{i}/w_{i}$ normalized to (0.3459,0.3672,0.2869) and the inverse weight of Corollary 12, normalized to (0.3407,0.3651,0.2942): all four lie in the interior of the same solid cone, drawn in Figure 1. What the analytic constructions of Section 9.2 supply is a point; what the conic formulation supplies is the set the point lives in.

The objective matters. Maximizing ε maximizes distance from the boundary of the positive orthant, not the margin in the matrix inequality; the program of Corollary 3, which maximizes δ with $\mathrm{diag}\left(a\right)M+M^{T}\mathrm{diag}\left(a\right)⪯-2\delta I$, returns a different optimizer,

$$a^{\delta }\approx \left(0.3625,\;0.3475,\;0.2900\right),\;\delta^{*}\approx 0.5399,$$

against δ≈0.5301 at the barycenter: the weight giving the fastest guaranteed decay of $L_{a}$ is not the weight furthest inside the orthant. Both are admissible; they answer different questions.

**Instance 2: a cyclic feedback loop.** Now let

$$M\left(K\right)=\left(\begin{array}{ccc} -1 & 1 & 0\\ 0 & -1 & 1\\ -K & 0 & -1 \end{array}\right),\;K>0,$$

a three-species chain in which species 3 inhibits species 1 with strength *K*, closing the loop. M(K) is not Metzler for any K>0, so Theorem 4 and Remark 21 do not apply, and no analytic weight is available. Its characteristic equation is ${\left(1+\lambda \right)}^{3}=-K$, with the complex pair having real part $-1+K^{1/3}/2$, so M(K) is Hurwitz exactly for K<8, losing stability through a Hopf bifurcation at K=8. Solving (4) over K∈{1,2,4,6,7,7.9,7.99}, the program is feasible with $\varepsilon^{*}>0$ for every K<8 tested, and bisection locates the loss of feasibility at K=8 to within $5\times 10^{-8}$: the admissible cone survives the entire stability range and empties exactly at the Hopf point, so for this family Volterra weights are not merely sufficient for stability but coextensive with it. The transition at K=2 visible in the table is exact: q=(1,0,−1) gives $q^{T}\left(M+M^{T}\right)q=2K-4$, so the uniform weight is admissible iff K≤2, with $(M+M^{T})q=0$ at K=2. Past that threshold the optimizer tilts sharply, loading species 1 and starving species 3: the Lyapunov function must charge the species that closes the inhibitory loop as little as possible, and the weight it can afford to give it, $a_{3}^{*}=\varepsilon^{*}$, is precisely the quantity that measures how far the community is from having no Volterra certificate at all.

Note that $\varepsilon^{*}$ does not detect the Hopf point—it flattens near 0.0477 as K↑8—whereas the margin of Corollary 3 does: $\delta^{*}$ falls from 0.0122 at K=6 to 0.00046 at K=7.9, vanishing at K=8. The feasibility program decides existence; the margin program measures robustness, and only the second sees the bifurcation coming.

Both instances are solved by the semidefinite program (4) directly, or equivalently by minimizing $\lambda_{\max}\left(\mathrm{diag}\left(a\right)M+M^{T}\mathrm{diag}\left(a\right)\right)$ over the simplex, a convex program whose minimum is therefore global; the values were cross-checked against the exact statements above wherever one exists.

## 5. The Admissible-Weight Cone and Perron–Volterra Relay Theory

We return to $W(x^{*})$ of Section 4.1, now informed by the explicit weak-competition example of Section 6.1.2, and go beyond convexity and the two rescaling laws already established, addressing shape, dimension, and the gap between the necessary condition of Proposition 4 and genuine admissibility.

### 5.1. The Two-Dimensional Cone Is Always Polyhedral

A convex cone in $R^{2}$ is necessarily an angular sector, generated by at most two rays, hence polyhedral: this is elementary convex geometry, independent of any GLV-specific structure. The determinant condition of Theorem 3 is quadratic in $a=(a_{1},a_{2})$, but it is *homogeneous* of degree two; its zero set, after passing to the ratio $r=a_{2}/a_{1}$, collapses to an interval condition rather than a curved arc. The following theorem makes this exact.

**Theorem** **5** (Exact two-dimensional admissible-weight cone [2d-weight-cone])**.** Let $f\left(x\right)=M\left(x-x^{*}\right)$, $M=\left(\begin{array}{cc} -a_{11} & q_{12}\\ q_{21} & -a_{22} \end{array}\right)$, $a_{11},a_{22}>0$, and $W\left(x^{*}\right)=\left\{a\in R_{>0}^{2}:\mathrm{diag}\left(a\right)M+M^{T}\mathrm{diag}\left(a\right)⪯0\right\}$. Put $r=a_{2}/a_{1}$ and $\Psi \left(r\right)=q_{21}^{2}r^{2}+\left(2q_{12}q_{21}-4a_{11}a_{22}\right)r+q_{12}^{2}$. Then $a\in W(x^{*})\Leftrightarrow r>0$ and Ψ(r)≤0; consequently $W(x^{*})$ is always a polyhedral cone in $R^{2}$. More precisely:

*(i)*  if $q_{12}q_{21}>a_{11}a_{22}$, then $W(x^{*})=⌀$;
*(ii)*  if $q_{21}\neq 0$ and $q_{12}q_{21}\leq a_{11}a_{22}$, with $r_{\pm }=\left(2a_{11}a_{22}-q_{12}q_{21}\pm 2\sqrt{a_{11}a_{22}\left(a_{11}a_{22}-q_{12}q_{21}\right)}\right)/q_{21}^{2}$, then $0\leq r_{-}\leq r_{+}$ and $W\left(x^{*}\right)=\left\{a_{1}\left(1,r\right):a_{1}>0,\;r\in \left[r_{-},r_{+}\right]\cap R_{>0}\right\}$;
*(iii)*  if $q_{21}=0$, $q_{12}\neq 0$, $W\left(x^{*}\right)=\left\{a_{1}\left(1,r\right):a_{1}>0,\;r\geq q_{12}^{2}/\left(4a_{11}a_{22}\right)\right\}$;
*(iv)*  if $q_{12}=0$, $q_{21}\neq 0$, $W\left(x^{*}\right)=\left\{a_{1}\left(1,r\right):a_{1}>0,\;0<r\leq 4a_{11}a_{22}/q_{21}^{2}\right\}$;
*(v)*  if $q_{12}=q_{21}=0$, $W\left(x^{*}\right)=R_{>0}^{2}$.

**Proof.**  For $a\in R_{>0}^{2}$, $\mathrm{diag}\left(a\right)M+M^{T}\mathrm{diag}\left(a\right)=\left(\begin{array}{cc} -2a_{11}a_{1} & q_{12}a_{1}+q_{21}a_{2}\\ q_{12}a_{1}+q_{21}a_{2} & -2a_{22}a_{2} \end{array}\right)$; both diagonal entries are strictly negative, so by Sylvester’s criterion the matrix is negative semidefinite iff its determinant is nonnegative, i.e., $4a_{11}a_{22}a_{1}a_{2}-{\left(q_{12}a_{1}+q_{21}a_{2}\right)}^{2}\geq 0$. Dividing by $a_{1}^{2}>0$ and setting $r=a_{2}/a_{1}$ gives $4a_{11}a_{22}r-{\left(q_{12}+q_{21}r\right)}^{2}\geq 0$, equivalently Ψ(r)≤0 (verified symbolically and against 1000 random instances). If $q_{21}\neq 0$, the discriminant of Ψ is $\Delta_{\Psi }={\left(2q_{12}q_{21}-4a_{11}a_{22}\right)}^{2}-4q_{12}^{2}q_{21}^{2}=16a_{11}a_{22}\left(a_{11}a_{22}-q_{12}q_{21}\right)$, so real roots exist iff $q_{12}q_{21}\leq a_{11}a_{22}$, and then $r_{\pm }$ are as displayed (leading coefficient $q_{21}^{2}>0$, so $\Psi \left(r\right)\leq 0\Leftrightarrow r\in \left[r_{-},r_{+}\right]$); intersecting with r>0 gives (ii). $r_{-}\geq 0$ follows from ${\left(2a_{11}a_{22}-q_{12}q_{21}\right)}^{2}-4a_{11}a_{22}\left(a_{11}a_{22}-q_{12}q_{21}\right)=q_{12}^{2}q_{21}^{2}\geq 0$, so $2a_{11}a_{22}-q_{12}q_{21}\geq 2\sqrt{a_{11}a_{22}\left(a_{11}a_{22}-q_{12}q_{21}\right)}$. If $q_{21}=0$, the condition reduces to $4a_{11}a_{22}r-q_{12}^{2}\geq 0$, giving (iii); (iv) is symmetric with $q_{12}=0$; (v) is immediate.    □

**Corollary** **13** (Extreme rays and dimension)**.** Every nonempty closed admissible cone $\bar{W}\left(x^{*}\right)=\left\{a\in R_{\geq 0}^{2}:\mathrm{diag}\left(a\right)M+M^{T}\mathrm{diag}\left(a\right)⪯0\right\}$ is generated by at most two rays. If $q_{21}\neq 0$ and $q_{12}q_{21}<a_{11}a_{22}$, its two boundary rays are $R_{\geq 0}\left(1,r_{-}\right)$, $R_{\geq 0}\left(1,r_{+}\right)$, and $W(x^{*})$ has nonempty interior in $R^{2}$. If $q_{12}q_{21}=a_{11}a_{22}$, then $r_{-}=r_{+}=q_{12}/q_{21}$ (when $q_{12}q_{21}>0$) and $\bar{W}\left(x^{*}\right)=R_{\geq 0}\left(1,q_{12}/q_{21}\right)$: a one-dimensional admissible cone occurs precisely at this degenerate diagonal-stability threshold.

**Proof.**  Immediate from Theorem 5: when $q_{12}q_{21}<a_{11}a_{22}$, $\Delta_{\Psi }>0$ and the normalized admissible set is a nondegenerate interval $\left[r_{-},r_{+}\right]$; when $q_{12}q_{21}=a_{11}a_{22}$, $\Delta_{\Psi }=0$ and the interval collapses to the single point $r_{-}=r_{+}$.    □

**Corollary** **14** (Strict diagonal stability in dimension two)**.** *There exists a>0 with $\mathrm{diag}\left(a\right)M+M^{T}\mathrm{diag}\left(a\right)≺0$ if and only if $q_{12}q_{21}<a_{11}a_{22}$, i.e., iff $\det\left(-M\right)=a_{11}a_{22}-q_{12}q_{21}>0$: the admissible-weight cone has nonempty interior exactly under this condition.*

**Proof.**  Strict negative definiteness is strict positivity of the determinant, $4a_{11}a_{22}a_{1}a_{2}-{\left(q_{12}a_{1}+q_{21}a_{2}\right)}^{2}>0$; by the proof of Theorem 5, such a ratio *r* exists iff Ψ is strictly negative somewhere, iff $\Delta_{\Psi }>0$, iff $q_{12}q_{21}<a_{11}a_{22}$.    □

This resolves Section 5.2 completely in two dimensions: $W(x^{*})$ is always polyhedral there, is one-dimensional exactly at the threshold $q_{12}q_{21}=a_{11}a_{22}$ (with $q_{12}q_{21}>0$), has nonempty interior exactly when det(−M)>0, and is empty when $q_{12}q_{21}>a_{11}a_{22}$. Note det(−M)=Δ of Section 6.1.2 in the competitive case $q_{12}=-b_{12}$, $q_{21}=-b_{21}$: the weak-competition hypothesis Δ>0 is exactly the condition for $W(E_{12})$ to have nonempty interior, and the specific weights $\left(b_{21},b_{12}\right)$ used there are one interior point of the (now fully explicit) interval, not a boundary or unique choice.

The two extreme rays of the sector have a product that does not depend on the diagonal entries at all, which pins down a canonical weight inside the cone.

**Corollary** **15** (The geometric centre of the two-dimensional cone)**.** *Let $q_{21}\neq 0$ and $q_{12}q_{21}\leq a_{11}a_{22}$, so that Theorem 5(ii) gives the admissible ratio interval $\left[r_{-},r_{+}\right]$ for $r=a_{2}/a_{1}$. Then*

$$r_{-}r_{+}=\frac{q_{12}^{2}}{q_{21}^{2}},\;\mathrm{hence}\;\sqrt{r_{-}r_{+}}=\left|\frac{q_{12}}{q_{21}}\right|,$$

*so the geometric mean of the two extreme rays is determined by the off-diagonal entries alone.*

**Proof.**  $r_{\pm }$ are the roots of $\Psi \left(r\right)=q_{21}^{2}r^{2}+\left(2q_{12}q_{21}-4a_{11}a_{22}\right)r+q_{12}^{2}$, so by Viète their product is the ratio of the constant to the leading coefficient, $q_{12}^{2}/q_{21}^{2}$, independently of $a_{11},a_{22}$; both roots are nonnegative (Theorem 5(ii)), so the square root is their geometric mean.    □

Section 6 identifies this center, for the competitive affine family, with the cross-coefficient weight that certifies exclusion.

### 5.2. Nonpolyhedrality Can Only Occur from Three Species on

**Remark** **22.** Since every convex cone in $R^{2}$ is polyhedral, genuine spectrahedral (non-polyhedral) admissible cones can first occur only for n≥3: the right question is not whether $W(x^{*})$ is polyhedral in general, but for which n≥3 and which matrices M the cone $\left\{a\geq 0:\mathrm{diag}\left(a\right)M+M^{T}\mathrm{diag}\left(a\right)⪯0\right\}$ has a genuinely curved boundary. In the general n-dimensional case, $W(x^{*})$, when nonempty, either has nonempty interior (the generic case, by the same continuity argument as Corollary 14: strict negative definiteness is an open condition) or is confined to a lower-dimensional semidefinite-only locus (a non-generic, codimension-≥1 coincidence, as in Section 9.1’s optimal weight, found with one zero eigenvalue). A full classification for n≥3 is left open.

### 5.3. Conic Structure of the Admissible Cone

Remark 22 asks for which n≥3 and which *M* the cone $W(x^{*})$ has a genuinely curved boundary, and Remark 15 asks whether it can be described by finitely many extreme rays for n≥3. Both are questions about a spectrahedron, so both can be examined computationally on any given instance. We do this for the non-Metzler matrix of Section 4.1, $M=\left(\begin{array}{ccc} -4 & 1 & 2\\ 2 & -5 & 1\\ 1 & 3 & -6 \end{array}\right)$, whose cone is nonempty and solid by the computation there.

**The boundary is an explicit cubic.** With $S\left(a\right)=\mathrm{diag}\left(a\right)M+M^{T}\mathrm{diag}\left(a\right)$, exact expansion gives

(8) $$\det S\left(a\right)=2\left(22a_{1}^{2}a_{2}+12a_{1}^{2}a_{3}+8a_{1}a_{2}^{2}-399a_{1}a_{2}a_{3}+39a_{1}a_{3}^{2}+26a_{2}^{2}a_{3}+11a_{2}a_{3}^{2}\right),$$

a cubic form that is irreducible over Q. It carries no pure cube $a_{i}^{3}$, and cannot: every term of the determinant expansion is a product of three entries meeting all three rows and all three columns, and $S_{ij}$ depends only on $a_{i},a_{j}$, so no monomial can involve a single index three times. Since S(a) has strictly negative diagonal for a≫0, Sylvester’s criterion in the semidefinite form gives

$${\bar{W}}_{1}=\left\{a\geq 0:\;\sum_{i}a_{i}=1,\;\det S\left(a\right)\leq 0,\;\mathrm{all}\;\mathrm{three}\;2\times 2\;\mathrm{principal}\;\mathrm{minors}\;\geq 0\right\},$$

the second-order minors being $-a_{1}^{2}+76a_{1}a_{2}-4a_{2}^{2}$ and its two analogues. Of these constraints only the determinant can bind on the boundary, and this needs no computation: the set {a≫0:S(a)≺0} is open, by continuity of the eigenvalues of S(a) in *a*, and is contained in ${\bar{W}}_{1}$; hence a point of the relative boundary satisfies S(a)⪯0 but not S(a)≺0, so S(a) is singular and

detS(a)=0.

The second-order minors are therefore never the active constraint there, whatever their values. Tracing the boundary radially from the barycenter in 720 directions agrees, putting every boundary point on detS(a)=0 to within $4\times 10^{-14}$.

**The boundary is curved everywhere.** For this *M* the cone is genuinely spectrahedral and not polyhedral, which is the instance-level answer Remark 22 asks for at n=3, the smallest dimension where Theorem 5 no longer forces polyhedrality. This is not a numerical observation but an exact one. Writing a line in the slice as a=p+tq with $\sum_{i}p_{i}=1$, $\sum_{i}q_{i}=0$, q≠0, the line lies on (8) iff all four coefficients of the cubic polynomial t↦detS(p+tq) vanish; normalizing *q* and computing a Gröbner basis of the resulting system in the remaining three unknowns returns {1} in each branch, so the system has no solution over C, let alone over R. The plane cubic (8) contains no line at all; since the boundary lies on that cubic, it contains no segment.

A boundary without segments makes every boundary ray extreme, so $W(x^{*})$ here has a continuum of extreme rays and admits no finite generating set: the finite description available in dimension two (Corollary 13, at most two rays) has no analogue already at n=3. This answers the extreme-ray question of Remark 15 negatively for this instance, and the answer is exact throughout: the boundary lies on detS=0 by the argument above, and that cubic contains no line by the Gröbner computation.

**The cone is bounded away from the faces.** The smallest coordinate attained anywhere on the traced boundary is 0.0354, so ${\bar{W}}_{1}$ meets no face $a_{i}=0$ of the simplex, and $\bar{W}\left(x^{*}\right)\setminus \left\{0\right\}\subset R_{>0}^{3}$: not merely does an admissible weight exist, but no sequence of admissible weights can degenerate by sending one species’ weight to zero. Monte-Carlo integration over the simplex puts the admissible fraction at 0.68.

The same dual side of Theorem 3 settles a question the spectrum cannot. Diagonal stability is strictly stronger than Hurwitz stability: an affine GLV system may have every eigenvalue in the open left half-plane, and every diagonal entry negative, and still admit no Volterra weight at all. The obstruction is exhibited by three positive integers.

**Proposition** **6** (Hurwitz does not imply admissible weights)**.** *Let*

$$M=\frac{1}{10}\left(\begin{array}{ccc} -15 & 24 & 17\\ -16 & -10 & 22\\ -30 & 19 & -10 \end{array}\right),\;\mathrm{so}\;f\left(x\right)=M\left(x-x^{*}\right)\;\mathrm{is}\;\mathrm{affine}\;\mathrm{with}\;Df\equiv M.$$

Then M is Hurwitz, with strictly negative diagonal, and $W(x^{*})=⌀$: no Volterra function $L_{a}$ with a≫0 is nonincreasing along the system, although $x^{*}$ is asymptotically stable. Hence, (DS) is strictly stronger than local asymptotic stability, and no argument deducing (DS) from the stability of a complex-balanced equilibrium alone could have established (G)+(CB)⇒(DS), which Theorem 11 in any case refutes.

**Proof.**  Both halves are exact integer computations. Write N=10M, which has the same admissible cone since the matrix inequality is homogeneous in *M*.

*Hurwitz.* The characteristic polynomial of *M* is $\lambda^{3}+\frac{7}{2}\lambda^{2}+\frac{219}{25}\lambda +\frac{12589}{500}$. Its Routh–Hurwitz conditions hold as exact rationals: $c_{2}=\frac{7}{2}>0$, $c_{0}=\frac{12589}{500}>0$, and $c_{2}c_{1}-c_{0}=\frac{7}{2}\cdot \frac{219}{25}-\frac{12589}{500}=\frac{2741}{500}>0$. (Numerically the eigenvalues are −3.2127 and −0.1436±2.7958i, so the equilibrium is a stable focus, not a marginal case.)

*Empty cone.* Put $u={\left(1,4,3\right)}^{T}$ and $P=uu^{T}⪰0$, a rank-one positive semidefinite matrix. Then $Nu={\left(132,10,16\right)}^{T}$, so

$$u_{1}{\left(Nu\right)}_{1}=132,\;u_{2}{\left(Nu\right)}_{2}=40,\;u_{3}{\left(Nu\right)}_{3}=48,$$

all strictly positive. For any *a* and $Q\left(a\right)=\mathrm{diag}\left(a\right)N+N^{T}\mathrm{diag}\left(a\right)$, cyclicity of the trace gives $\langle P,Q\left(a\right)\rangle =2\sum_{i}a_{i}{\left(NP\right)}_{ii}=2\sum_{i}a_{i}u_{i}{\left(Nu\right)}_{i}$, hence

$$\langle P,Q\left(a\right)\rangle =2\left(132a_{1}+40a_{2}+48a_{3}\right)>0\;\mathrm{for}\;\mathrm{every}\;a\gg 0.$$

But P⪰0 and Q(a)⪯0 would force 〈P,Q(a)〉≤0. So no a≫0 satisfies the matrix inequality, i.e., $W(x^{*})=⌀$ by Theorem 3. Independently, minimizing $\lambda_{\max}\left(Q\left(a\right)\right)$ over the simplex—a convex program, so its minimum is global—returns +0.6306>0, agreeing with the certificate.    □

### 5.4. Closing the Necessary/Sufficient Gap: Exact for Affine Systems, Genuinely Open Beyond It

**Corollary** **16** (Necessary is sufficient for affine vector fields [necsuff-affine])**.** If $f\left(x\right)=M\left(x-x^{*}\right)$, then $a\in W(x^{*})$ if and only if a satisfies the necessary condition of Proposition 4 at $x^{*}$, i.e., $\mathrm{diag}\left(a\right)J+J^{T}\mathrm{diag}\left(a\right)⪯0$ with J=M. There is no gap to close for the affine class: the SDP (Theorem 3) already is *the necessary condition, made sufficient by the absence of any remainder term.*

**Proof.**  Immediate from Theorem 3 and Proposition 4: the inclusion $W\left(x^{*}\right)\subseteq \left\{a:\mathrm{diag}\left(a\right)J+J^{T}\mathrm{diag}\left(a\right)⪯0\right\}$ is Proposition 4, and Theorem 3 proves the reverse inclusion for *f* affine.    □

**Theorem** **6** (Exact averaged-Jacobian characterization and the local-to-global gap [gap-example])**.** *Consider*

$$x^{\prime }=\mathrm{diag}\left(x\right)f\left(x\right),\;x\in R_{>0}^{n},$$

*where*

$$f\in C^{1}\left(R_{>0}^{n},R^{n}\right),\;f\left(x^{*}\right)=0,\;x^{*}\in R_{>0}^{n}.$$

*For a≫0, put*

A=diag(a)

and define the averaged Jacobian from $x^{*}$ to x by

$$\bar{J}\left(x;x^{*}\right):=\int_{0}^{1}Df\;\left(x^{*}+\theta \left(x-x^{*}\right)\right)\;d\theta .$$

*(i)*  *(Exact averaged-Jacobian characterization.) For every $x\in R_{>0}^{n}$,*
  $$f\left(x\right)=\bar{J}\left(x;x^{*}\right)\left(x-x^{*}\right)\;(\mathrm{fundamental}\;\mathrm{theorem}\;\mathrm{of}\;\mathrm{calculus}\;\mathrm{along}\;a\;\mathrm{segment})$$
  *and*
  $$\begin{array}{c} {\dot{L}}_{a}\left(x\right)=\frac{1}{2}{\left(x-x^{*}\right)}^{T}\left[A\bar{J}\left(x;x^{*}\right)+\bar{J}{\left(x;x^{*}\right)}^{T}A\right]\left(x-x^{*}\right), \end{array}$$
  *where*
  $$L_{a}\left(x\right)=\sum_{i}a_{i}x_{i}^{*}G\left(x_{i}/x_{i}^{*}\right),\;G\left(u\right)=u-1-\log u.$$
  *Consequently,*
  $$\begin{array}{c} a\in W\left(x^{*}\right)\Leftrightarrow {\left(x-x^{*}\right)}^{T}\left[A\bar{J}\left(x;x^{*}\right)+\bar{J}{\left(x;x^{*}\right)}^{T}A\right]\left(x-x^{*}\right)\leq 0\;\mathrm{for}\;\mathrm{every}\;x\in R_{>0}^{n}. \end{array}$$
  *Define the averaged-Jacobian cone*
  $$W_{\mathrm{AJ}}\left(x^{*}\right):=\left\{a\gg 0:A\bar{J}\left(x;x^{*}\right)+\bar{J}{\left(x;x^{*}\right)}^{T}A⪯0\;\mathrm{for}\;\mathrm{every}\;x\in R_{>0}^{n}\right\}.$$
  *Then*
  $$W_{\mathrm{AJ}}\left(x^{*}\right)\subseteq W\left(x^{*}\right).$$
  *If, for some δ>0,*
  $$A\bar{J}\left(x;x^{*}\right)+\bar{J}{\left(x;x^{*}\right)}^{T}A⪯-2\delta I\;\mathrm{for}\;\mathrm{every}\;x\in R_{>0}^{n},$$
  *then*
  $${\dot{L}}_{a}\left(x\right)\leq -\delta {∥x-x^{*}∥}^{2},$$
  and $x^{*}$ is globally asymptotically stable.
*(ii)*  *(Pointwise diagonal stability is a sufficient certificate.) If there exists δ>0 such that*
  $$A\;Df\left(x\right)+Df{\left(x\right)}^{T}A⪯-2\delta I\;\mathrm{for}\;\mathrm{every}\;x\in R_{>0}^{n},$$
  *then*
  $$A\bar{J}\left(x;x^{*}\right)+\bar{J}{\left(x;x^{*}\right)}^{T}A⪯-2\delta I\;\mathrm{for}\;\mathrm{every}\;x\in R_{>0}^{n}.$$
  *Hence*
  $$a\in W_{\mathrm{AJ}}\left(x^{*}\right)\subseteq W\left(x^{*}\right),$$
  *and*
  $${\dot{L}}_{a}\left(x\right)\leq -\delta {∥x-x^{*}∥}^{2}.$$
  Consequently, by Lemma 1, $x^{*}$ is globally asymptotically stable in $R_{>0}^{n}$.
*(iii)*  (The local condition is not sufficient.) There exists a nonlinear GLV system with a unique positive equilibrium $x^{*}$ and a weight vector a≫0 such that
  $$A\;Df\left(x^{*}\right)+Df{\left(x^{*}\right)}^{T}A≺0,$$
  *but*
  $$a\notin W(x^{*}).$$
  *Thus, even strict diagonal stability at the equilibrium does not imply global Volterra admissibility for nonlinear GLV systems.*

**Proof.** 

(i) Put
  $$y=x-x^{*}.$$
  Since $R_{>0}^{n}$ is convex, $x^{*}+\theta y\in R_{>0}^{n}$ for 0≤θ≤1. The fundamental theorem of calculus and $f(x^{*})=0$ give
  $$f\left(x\right)=\int_{0}^{1}Df\left(x^{*}+\theta y\right)y\;d\theta =\bar{J}\left(x;x^{*}\right)y.$$
  Moreover,
  $${\dot{L}}_{a}\left(x\right)=\sum_{i}a_{i}\left(x_{i}-x_{i}^{*}\right)f_{i}\left(x\right)=y^{T}Af\left(x\right),$$
  and therefore
  $${\dot{L}}_{a}\left(x\right)=y^{T}A\bar{J}\left(x;x^{*}\right)y.$$
  Since this is a scalar,
  $${\dot{L}}_{a}\left(x\right)=\frac{1}{2}y^{T}\left[A\bar{J}\left(x;x^{*}\right)+\bar{J}{\left(x;x^{*}\right)}^{T}A\right]y,$$
  which proves the displayed identity.
  By Definition 13,
  $$a\in W\left(x^{*}\right)\Leftrightarrow {\dot{L}}_{a}\left(x\right)\leq 0\;\mathrm{for}\;\mathrm{every}\;x\in R_{>0}^{n}.$$
  Substitution of the identity above gives the asserted exact characterization.
  If
  $$A\bar{J}\left(x;x^{*}\right)+\bar{J}{\left(x;x^{*}\right)}^{T}A⪯0$$
  for every *x*, the exact identity immediately gives ${\dot{L}}_{a}\left(x\right)\leq 0$, and hence
  $$W_{\mathrm{AJ}}\left(x^{*}\right)\subseteq W\left(x^{*}\right).$$
  The strict estimate with −2δI similarly yields
  $${\dot{L}}_{a}\left(x\right)\leq -\delta {∥x-x^{*}∥}^{2}.$$
  Lemma 1 gives global asymptotic stability.
(ii) For every *x*,
  $$A\bar{J}\left(x;x^{*}\right)+\bar{J}{\left(x;x^{*}\right)}^{T}A=\int_{0}^{1}\left[A\;Df\left(x^{*}+\theta y\right)+Df{\left(x^{*}+\theta y\right)}^{T}A\right]d\theta .$$
  The hypothesis therefore gives
  $$A\bar{J}\left(x;x^{*}\right)+\bar{J}{\left(x;x^{*}\right)}^{T}A⪯-2\delta I.$$
  Part (i) gives all the stated conclusions.
(iii) Consider
  $$x^{\prime }=x\left(2.450-0.773x+0.195y-2.171xy\right),\;y^{\prime }=y\left(3.942+0.316x-0.507y-3.225xy\right),$$
  where the displayed decimals denote the corresponding exact rational numbers. Eliminating *y* from the positive-equilibrium equations gives
  $$3178961x^{2}+987123x-2010840=0.$$
  This polynomial has exactly one positive root,
  $$x^{*}=\frac{-987123+\sqrt{26543939566089}}{6357922},\;y^{*}=\frac{2450-773x^{*}}{13(167x^{*}-15)}.$$
  Since
  $$5152081^{2}<26543939566089<5152082^{2},$$
  one obtains the certified rational bounds
  $$x^{*}\in \left[\frac{2082479}{3178961},\frac{4164959}{6357922}\right],\;y^{*}\in \left[\frac{2739311}{1729572},\frac{5478623}{3459144}\right].$$
  In particular,
  $$\left(x^{*},y^{*}\right)\approx \left(0.6550817,1.5838087\right).$$
  For
  a=(1,0.5623),A=diag(a),
  direct substitution gives
  $$A\;Df\left(x^{*}\right)+Df{\left(x^{*}\right)}^{T}A≺0.$$
  Thus, even the strict form of the necessary local condition in Proposition 4 holds.
  At
  $$\left(x,y\right)=\left(\frac{1}{10000},\frac{891}{200}\right),$$
  however,
  $${\dot{L}}_{a}\left(x,y\right)=a_{1}\left(x-x^{*}\right)f_{1}\left(x,y\right)+a_{2}\left(y-y^{*}\right)f_{2}\left(x,y\right).$$
  Using the certified rational bounds above gives
  $${\dot{L}}_{a}\left(\frac{1}{10000},\frac{891}{200}\right)\geq \frac{554262388532120166127597186287}{45818682789100000000000000000}>12.09>0.$$
  Hence
  $$a\notin W(x^{*}).$$

   □

**Remark** **23** (gap-example-discussion [gap-example-discussion])**.** *The counterexample in Theorem 6 has a unique positive equilibrium, so the failure is genuinely nonlinear rather than a multistationarity effect. The local matrix*

$$A\;Df\left(x^{*}\right)+Df{\left(x^{*}\right)}^{T}A$$

controls the quadratic approximation of ${\dot{L}}_{a}$ at $x^{*}$, whereas global Volterra admissibility depends on the full secant behavior of f between $x^{*}$ and x.

*Indeed, Theorem 6(iii) introduces the averaged Jacobian*

$$\bar{J}\left(x;x^{*}\right)=\int_{0}^{1}Df\;\left(x^{*}+\theta \left(x-x^{*}\right)\right)\;d\theta$$

*and gives the exact identity*

$${\dot{L}}_{a}\left(x\right)=\frac{1}{2}{\left(x-x^{*}\right)}^{T}\left[A\bar{J}\left(x;x^{*}\right)+\bar{J}{\left(x;x^{*}\right)}^{T}A\right]\left(x-x^{*}\right).$$

Thus, the nonlinear obstruction is precisely the possible loss of dissipativity of the averaged Jacobian away from $x^{*}$.

*For affine systems,*

$$\bar{J}\left(x;x^{*}\right)=M,$$

so the exact affine criterion of Theorem 3 is recovered. For nonlinear systems, replacing M merely by the local Jacobian $Df(x^{*})$ discards the secant information entering the exact global criterion.

**Remark** **24** (Relation with the affine characterization)**.** *If*

$$f\left(x\right)=M\left(x-x^{*}\right),$$

*then*

$$Df\left(x\right)=M\;\mathrm{and}\;\bar{J}\left(x;x^{*}\right)=M$$

*for every x. Hence, Theorem 6(iii) reduces exactly to*

$$a\in W\left(x^{*}\right)\Leftrightarrow {\left(x-x^{*}\right)}^{T}\left(AM+M^{T}A\right)\left(x-x^{*}\right)\leq 0\;\mathrm{for}\;\mathrm{every}\;x>0,$$

*which is equivalent to*

$$AM+M^{T}A⪯0.$$

*Thus, part (iii) recovers the full affine characterization of Theorem 3, while part (ii) gives the convenient strict sufficient condition*

$$ADf\left(x\right)+Df{\left(x\right)}^{T}A⪯-2\delta I$$

*uniformly over the nonlinear Jacobian family.*

*The hierarchy is therefore*

$$\mathrm{local}\;Df\left(x^{*}\right)\;⊊\;\mathrm{exact}\;\mathrm{secant}\;\bar{J}\left(x;x^{*}\right)\;\subseteq \;\mathrm{uniform}\;\mathrm{pointwise}\;\mathrm{control}\;\mathrm{of}\;Df\left(x\right),$$

*where the middle level gives the exact Volterra criterion and the last one is a stronger, readily verifiable sufficient condition.*

**Corollary** **17** (Invariant-region averaged-Jacobian criterion)**.** Let $\Omega \subseteq R_{>0}^{n}$ be convex and forward invariant, with $x^{*}\in \Omega$, and define

$${\bar{J}}_{\Omega }\left(x;x^{*}\right)=\int_{0}^{1}Df\;\left(x^{*}+\theta \left(x-x^{*}\right)\right)\;d\theta ,\;x\in \Omega .$$

*If*

$${\left(x-x^{*}\right)}^{T}\left[A{\bar{J}}_{\Omega }\left(x;x^{*}\right)+{\bar{J}}_{\Omega }{\left(x;x^{*}\right)}^{T}A\right]\left(x-x^{*}\right)\leq 0\;\mathrm{for}\;\mathrm{every}\;x\in \Omega ,$$

*then*

$${\dot{L}}_{a}\left(x\right)\leq 0\;\left(x\in \Omega \right).$$

*If, more strongly, there exists δ>0 such that*

$$A{\bar{J}}_{\Omega }\left(x;x^{*}\right)+{\bar{J}}_{\Omega }{\left(x;x^{*}\right)}^{T}A⪯-2\delta I\;\mathrm{for}\;\mathrm{every}\;x\in \Omega ,$$

*then*

$${\dot{L}}_{a}\left(x\right)\leq -\delta {∥x-x^{*}∥}^{2}\;\left(x\in \Omega \right).$$

Consequently, $x^{*}$ is globally asymptotically stable relative to Ω *whenever trajectories in* Ω *are precompact.*

**Proof.**  Convexity of Ω implies that, for every x∈Ω, the segment

$$x^{*}+\theta \left(x-x^{*}\right),\;0\leq \theta \leq 1,$$

lies in Ω. The exact identity of Theorem 6(iii) therefore gives

$${\dot{L}}_{a}\left(x\right)=\frac{1}{2}{\left(x-x^{*}\right)}^{T}\left[A{\bar{J}}_{\Omega }\left(x;x^{*}\right)+{\bar{J}}_{\Omega }{\left(x;x^{*}\right)}^{T}A\right]\left(x-x^{*}\right).$$

The two conclusions follow immediately from the corresponding hypotheses. In the strict case, LaSalle’s invariance principle gives global asymptotic stability relative to Ω under precompactness.    □

**Corollary** **18** (Pointwise Jacobian criterion on an invariant region)**.** Let $\Omega \subseteq R_{>0}^{n}$ be convex and forward invariant, with $x^{*}\in \Omega$. If, for some diagonal A≫0 and δ>0,

$$A\;Df\left(x\right)+Df{\left(x\right)}^{T}A⪯-2\delta I\;\mathrm{for}\;\mathrm{every}\;x\in \Omega ,$$

*then*

$${\dot{L}}_{a}\left(x\right)\leq -\delta {∥x-x^{*}∥}^{2}\;\mathrm{for}\;\mathrm{every}\;x\in \Omega .$$

Consequently, $x^{*}$ is globally asymptotically stable relative to Ω *whenever trajectories in* Ω *are precompact.*

**Proof.**  For x∈Ω, integrate the assumed matrix inequality along the segment joining $x^{*}$ to *x*. This gives

$$A{\bar{J}}_{\Omega }\left(x;x^{*}\right)+{\bar{J}}_{\Omega }{\left(x;x^{*}\right)}^{T}A⪯-2\delta I.$$

The conclusion follows from Corollary 17.    □

**Corollary** **19** (Perturbative criterion)**.** *Suppose*

$$f\left(x\right)=M\left(x-x^{*}\right)+h\left(x\right),\;h\left(x^{*}\right)=0,$$

*and let A=diag(a)≫0. Assume*

$$AM+M^{T}A⪯-2\gamma I$$

for some γ>0, and, on a convex forward-invariant set $\Omega \subseteq R_{>0}^{n}$ containing $x^{*}$,

$$\underset{x\in \Omega }{\sup}{∥A\;Dh\left(x\right)+Dh{\left(x\right)}^{T}A∥}_{2}<2\gamma .$$

*Then there exists δ>0 such that*

$${\dot{L}}_{a}\left(x\right)\leq -\delta {∥x-x^{*}∥}^{2}\;\left(x\in \Omega \right).$$

*In particular,*

$$a\in W_{\Omega }\left(x^{*}\right),$$

and $L_{a}$ is strictly decreasing on $\Omega \setminus \{x^{*}\}$.

**Proof.**  Put

$$\eta =\frac{1}{2}\underset{x\in \Omega }{\sup}{∥A\;Dh\left(x\right)+Dh{\left(x\right)}^{T}A∥}_{2}<\gamma .$$

Since

Df(x)=M+Dh(x),

one has

$$A\;Df\left(x\right)+Df{\left(x\right)}^{T}A⪯-2\left(\gamma -\eta \right)I\;\left(x\in \Omega \right).$$

Apply Corollary 18 with

δ=γ−η>0.

   □

**Remark** **25** (What remains open)**.** *Theorem 6 gives three distinct levels of information. The local condition*

$$A\;Df\left(x^{*}\right)+Df{\left(x^{*}\right)}^{T}A⪯0$$

*is necessary for Volterra admissibility. The averaged-Jacobian identity*

$${\dot{L}}_{a}\left(x\right)=\frac{1}{2}{\left(x-x^{*}\right)}^{T}\left[A\bar{J}\left(x;x^{*}\right)+\bar{J}{\left(x;x^{*}\right)}^{T}A\right]\left(x-x^{*}\right)$$

*gives the exact global criterion. Uniform diagonal stability of the pointwise Jacobian family is a stronger sufficient condition which implies the averaged inequality by integration.*

*The remaining problem is therefore to obtain finite and computable conditions, preferably from the E-graph or directly from the coefficients of the generalized polynomial system, which certify the averaged-Jacobian inequality*

$${\left(x-x^{*}\right)}^{T}\left[A\bar{J}\left(x;x^{*}\right)+\bar{J}{\left(x;x^{*}\right)}^{T}A\right]\left(x-x^{*}\right)\leq 0$$

*on the relevant invariant region. Such criteria may exploit cancellations along secants and therefore need not imply pointwise diagonal stability of every Df(x).*

### 5.5. A Relay Theorem for Block GLV Models: Siphons, Perron Weights, and a Unified Lyapunov Formula

The relay construction is naturally formulated on siphon faces and specializes in dimension one to scalar GLV systems. The siphon determines the form of the Lyapunov function: resident blocks contribute Volterra entropy terms, while missing blocks contribute positive linear Perron functionals. The scalar GLV formula is the one-dimensional special case.

**Definition** **16** (Relay sink [Rel])**.** *Let σ be a siphon and let*

$$E\in F_{\sigma }^{\circ }$$

*be a resident equilibrium. Let*

$$J_{\sigma }^{⊥}\left(E\right)$$

*be the transversal Jacobian and let*

$$B_{1}\left(E\right),\ldots ,B_{q}\left(E\right)$$

*be its irreducible Frobenius diagonal blocks. We call E a *relay sink *if*

$$s(B_{r}\left(E\right))<0,\;r=1,\ldots ,q.$$

*Equivalently,*

$$s\;\left(J_{\sigma }^{⊥}\left(E\right)\right)<0.$$

*For every block admitting a regular next-generation splitting*

$$B_{r}\left(E\right)=F_{r}\left(E\right)-V_{r}\left(E\right),$$

*the same condition is*

$$R_{r}\left(E\right):=\rho \;\left(F_{r}\left(E\right)V_{r}{\left(E\right)}^{-1}\right)<1.$$

For each block, let $c_{k}\gg 0$ denote the positive Perron functional used in the Perron–Volterra construction. In the rank-one next-generation setting

$$F_{k}=w_{k}\pi_{k}^{T},$$

the canonical normalization is

$$R_{k}=\pi_{k}^{T}V_{k}^{-1}w_{k},\;c_{k}^{T}=\frac{\pi_{k}^{T}V_{k}^{-1}}{R_{k}},\;c_{k}^{T}w_{k}=1.$$

For a resident block k∉σ, write

$$G\left(z_{k},z_{k}^{*}\right)={\left(z_{k,j}^{*}G\;\left(\frac{z_{k,j}}{z_{k,j}^{*}}\right)\right)}_{j=1}^{n_{k}},\;G\left(u\right)=u-1-\log u.$$

The same definition applies to an *environment block y* (possibly vector-valued), with its own positive functional $c_{0}\gg 0$; when *y* is scalar and $c_{0}=1$, $c_{0}^{T}G\left(y,y^{*}\right)$ reduces to the familiar $y^{*}G\left(y/y^{*}\right)$.

**Theorem** **7** (Perron–Volterra relay theorem [relay-theorem])**.** *Let σ be a siphon and let*

$$E=\left(y_{E},{\left(z_{k}^{E}\right)}_{k\notin \sigma },0\right)\in F_{\sigma }^{\circ }$$

*be a resident equilibrium in $F_{\sigma }^{\circ }$, where y is the environment block introduced above. The pair (σ,E) determines the transversal Jacobian*

$$J_{\sigma }^{⊥}\left(E\right).$$

*Fix its Frobenius decomposition, with irreducible Metzler diagonal blocks*

$$B_{1}\left(E\right),\ldots ,B_{q}\left(E\right),$$

*and let $Z_{r}$ denote the transversal variables corresponding to $B_{r}\left(E\right)$. For each transversal block choose a positive left Perron functional*

$$c_{r}^{T},\;r=1,\ldots ,q.$$

*Let $H_{\sigma ,E}$ be a Perron–Volterra entropy for the resident subsystem, of the form*

$$H_{\sigma ,E}=c_{0}^{T}G\left(y,y_{E}\right)+\sum_{k\notin \sigma }c_{k}^{T}G\left(z_{k},z_{k}^{E}\right),\;G\left(u\right)=u-1-\log u,$$

*with*

$$G\left(z_{k},z_{k}^{E}\right)={\left(z_{k,j}^{E}G\;\left(\frac{z_{k,j}}{z_{k,j}^{E}}\right)\right)}_{j=1}^{n_{k}}.$$

*Define*

(9) $$\begin{array}{c} L_{\sigma ,E}=H_{\sigma ,E}+\sum_{r=1}^{q}c_{r}^{T}Z_{r}. \end{array}$$

*Assume that on the positively invariant persistence class under consideration the following conditions hold.*

*1.*  *Perron–Volterra decomposition and resident closing. Along solutions,*
  (10) $${\dot{L}}_{\sigma ,E}=D_{\sigma ,E}+\sum_{r=1}^{q}\eta_{r}\left(x;E\right)c_{r}^{T}Z_{r},$$
  *where*
  $$D_{\sigma ,E}\leq 0.$$
  *Moreover, the largest invariant subset of*
  $$\left\{x:D_{\sigma ,E}\left(x\right)=0,\;Z_{1}=\cdots =Z_{q}=0\right\}$$
  *is {E}.*
*2.*  *Transversal closing. For every r,*
  $$\eta_{r}\left(x;E\right)<0\;\mathrm{whenever}\;Z_{r}\neq 0.$$
*3.*  *Compactness. The sublevel sets of $L_{\sigma ,E}$ in the persistence class are compact.*
  *Then*

$${\dot{L}}_{\sigma ,E}\leq 0,$$

*and the largest invariant subset of*

$$\left\{{\dot{L}}_{\sigma ,E}=0\right\}$$

*is {E}. Consequently, E is globally asymptotically stable on the persistence class.* 

*Suppose, moreover, that the model-specific Perron–Volterra calculation gives, whenever $Z_{r}\neq 0$,*

(11) $$\mathrm{sign}\eta_{r}\left(x;E\right)=\mathrm{sign}s\left(B_{r}\left(E\right)\right),\;r=1,\ldots ,q.$$

*Then the transversal closing condition is equivalent to*

$$s(B_{r}\left(E\right))<0,\;r=1,\ldots ,q.$$

*Hence, every relay-sink equilibrium satisfying the resident closing and compactness conditions is globally asymptotically stable.*

*For every block admitting a regular next-generation splitting*

$$B_{r}\left(E\right)=F_{r}\left(E\right)-V_{r}\left(E\right),\;F_{r}\left(E\right)\geq 0,$$

*with $V_{r}\left(E\right)$ a nonsingular M-matrix, put*

$$R_{r}\left(E\right)=\rho \;\left(F_{r}\left(E\right)V_{r}{\left(E\right)}^{-1}\right).$$

*Then*

$$s\left(B_{r}\left(E\right)\right)<0\Leftrightarrow R_{r}\left(E\right)<1.$$

*Thus, the pair (σ,E) determines the transversal problem through $J_{\sigma }^{⊥}\left(E\right)$, while its irreducible Frobenius blocks determine the individual Perron or next-generation invasion thresholds.*

**Proof.**  The siphon σ determines the invariant face and the equilibrium $E\in F_{\sigma }^{\circ }$ determines the point at which transversal stability is evaluated. The corresponding transversal matrix is

$$J_{\sigma }^{⊥}\left(E\right).$$

Its Frobenius decomposition separates the missing subsystem into the irreducible Metzler blocks $B_{r}\left(E\right)$, and Perron–Frobenius theory provides positive left functionals $c_{r}^{T}$.

By (10),

$${\dot{L}}_{\sigma ,E}=D_{\sigma ,E}+\sum_{r=1}^{q}\eta_{r}\left(x;E\right)c_{r}^{T}Z_{r}.$$

For every *r*,

$$c_{r}\gg 0,\;Z_{r}\geq 0,$$

and hence

$$c_{r}^{T}Z_{r}\geq 0,$$

with

$$c_{r}^{T}Z_{r}=0\Leftrightarrow Z_{r}=0.$$

The resident term satisfies

$$D_{\sigma ,E}\leq 0.$$

If $Z_{r}\neq 0$, the transversal closing condition gives

$$\eta_{r}\left(x;E\right)c_{r}^{T}Z_{r}<0,$$

while for $Z_{r}=0$ the corresponding term vanishes. Therefore

$${\dot{L}}_{\sigma ,E}\leq 0.$$

Now let a trajectory lie in

$$\{{\dot{L}}_{\sigma ,E}=0\}.$$

Every term in (10) is nonpositive. Hence, for each *r*,

$$\eta_{r}\left(x;E\right)c_{r}^{T}Z_{r}=0.$$

The strict transversal closing condition whenever $Z_{r}\neq 0$ implies

$$Z_{r}=0,\;r=1,\ldots ,q.$$

The derivative identity then reduces to

$${\dot{L}}_{\sigma ,E}=D_{\sigma ,E},$$

so along such a trajectory

$$D_{\sigma ,E}=0.$$

Thus, every invariant subset of $\left\{{\dot{L}}_{\sigma ,E}=0\right\}$ is contained in

$$\left\{x:D_{\sigma ,E}\left(x\right)=0,\;Z_{1}=\cdots =Z_{q}=0\right\}.$$

By the resident closing hypothesis, the largest invariant subset of this set is {E}. Hence, the largest invariant subset of $\left\{{\dot{L}}_{\sigma ,E}=0\right\}$ is also {E}.

The compactness condition supplies compact positively invariant sublevel sets of $L_{\sigma ,E}$. LaSalle’s invariance principle therefore gives convergence of every trajectory in the persistence class to *E*, proving global asymptotic stability.

Under (11), for every $Z_{r}\neq 0$,

$$s\left(B_{r}\left(E\right)\right)<0\Leftrightarrow \eta_{r}\left(x;E\right)<0.$$

Thus, the relay-sink inequalities

$$s(B_{r}\left(E\right))<0,\;r=1,\ldots ,q,$$

are precisely the transversal closing conditions required above.

Finally, for a regular splitting

$$B_{r}\left(E\right)=F_{r}\left(E\right)-V_{r}\left(E\right),$$

the Metzler next-generation threshold theorem gives

$$s\left(B_{r}\left(E\right)\right)<0\Leftrightarrow \rho \;\left(F_{r}\left(E\right)V_{r}{\left(E\right)}^{-1}\right)<1,$$

which is the asserted $R_{r}\left(E\right)<1$ formulation.    □

The environment block *y* of Theorem 7 is left general there; the explicit rank-one realization below specializes it to a single common scalar environmental variable *s*, shared by every resident and transversal block.

**Corollary** **20** (Rank-one relay theorem on a multi-block resident face)**.** *Consider*

$$\left\{\begin{array}{c} s^{\prime }=\Lambda -\mu s-s\sum_{k=1}^{m}\phi_{k},\\ z_{k}^{\prime }=\left(sw_{k}\pi_{k}^{T}-V_{k}\right)z_{k},\;\phi_{k}=\pi_{k}^{T}z_{k}, \end{array}\right.$$

*with*

$$R_{k}=\pi_{k}^{T}V_{k}^{-1}w_{k}.$$

*Let I⊆{1,…,m} be a nonempty resident support and let*

$$E_{I}=\left(s_{I},{\left(z_{k}^{I}\right)}_{k\in I},0\right)$$

*be an equilibrium in the relative interior of the corresponding face. Then necessarily*

$$R_{k}=R_{I}\;\left(k\in I\right),\;s_{I}=\frac{1}{R_{I}}.$$

*For every missing block k∉I put*

$$c_{k}^{T}=\frac{\pi_{k}^{T}V_{k}^{-1}}{R_{k}}.$$

*Assume that the resident subsystem on the support I admits a Perron–Volterra entropy $H_{I}$ satisfying*

$${\dot{H}}_{I}\leq -\mu \frac{{\left(s-s_{I}\right)}^{2}}{s}-\left(s-s_{I}\right)\sum_{k\notin I}\phi_{k},$$

*and that the largest invariant subset of its zero-dissipation set, after all missing blocks are set equal to zero, is the resident equilibrium set selected on the face.*

*Then*

$$L_{I}=H_{I}+\sum_{k\notin I}c_{k}^{T}z_{k}$$

*satisfies*

$${\dot{L}}_{I}\leq -\mu \frac{{\left(s-s_{I}\right)}^{2}}{s}+\sum_{k\notin I}\left(\frac{1}{R_{I}}-\frac{1}{R_{k}}\right)\phi_{k}.$$

*Equivalently,*

$${\dot{L}}_{I}\leq -\mu \frac{{\left(s-s_{I}\right)}^{2}}{s}+\sum_{k\notin I}\frac{R_{k}\left(E_{I}\right)-1}{R_{k}}\phi_{k},\;R_{k}\left(E_{I}\right)=\frac{R_{k}}{R_{I}}.$$

*Hence*

$$R_{k}\left(E_{I}\right)<1,\;k\notin I,$$

*verifies the transversal closing condition of Theorem 7 at $E_{I}$.*

*If, in addition, the sublevel sets of $L_{I}$ in the persistence class are compact, then every trajectory in the persistence class converges to the largest invariant subset of the resident zero-dissipation set assumed above. In particular, if that set consists of $E_{I}$ alone, then $E_{I}$ is globally asymptotically stable on the persistence class.*

**Proof.**  *Necessity of $R_{k}=R_{I}$.* Fix k∈I. Since $z_{k}^{I}\neq 0$ is stationary at $s=s_{I}$,

$$0={\left(z_{k}^{I}\right)}^{\prime }=\left(s_{I}w_{k}\pi_{k}^{T}-V_{k}\right)z_{k}^{I},\;i.e.,\;V_{k}z_{k}^{I}=s_{I}\phi_{k}^{I}w_{k},\;\phi_{k}^{I}:=\pi_{k}^{T}z_{k}^{I}.$$

Hence, $z_{k}^{I}=s_{I}\phi_{k}^{I}V_{k}^{-1}w_{k}$; applying $\pi_{k}^{T}$ to both sides gives

$$\phi_{k}^{I}=s_{I}\phi_{k}^{I}\;\pi_{k}^{T}V_{k}^{-1}w_{k}=s_{I}\phi_{k}^{I}R_{k}.$$

Since $E_{I}$ lies in the relative interior of the face, $z_{k}^{I}\geq 0$, $z_{k}^{I}\neq 0$, and $\pi_{k}\gg 0$ give $\phi_{k}^{I}>0$, so this may be divided by $\phi_{k}^{I}$ to give $s_{I}R_{k}=1$ for every k∈I. Thus, all resident ratios $R_{k}$, k∈I, coincide with the common value $R_{I}:=1/s_{I}$, proving the stated necessary condition.

*Missing-block coupling.* For k∉I, exactly as in the single-block case, $c_{k}^{T}w_{k}=\pi_{k}^{T}V_{k}^{-1}w_{k}/R_{k}=1$ and $c_{k}^{T}V_{k}=\pi_{k}^{T}/R_{k}$, so

$$c_{k}^{T}z_{k}^{\prime }=c_{k}^{T}\left(sw_{k}\pi_{k}^{T}-V_{k}\right)z_{k}=s\left(c_{k}^{T}w_{k}\right)\phi_{k}-c_{k}^{T}V_{k}z_{k}=\left(s-\frac{1}{R_{k}}\right)\phi_{k}.$$

*Combining with the resident closing hypothesis.* Adding this over k∉I and using the assumed bound on ${\dot{H}}_{I}$,

$${\dot{L}}_{I}={\dot{H}}_{I}+\sum_{k\notin I}c_{k}^{T}z_{k}^{\prime }\leq -\mu \frac{{\left(s-s_{I}\right)}^{2}}{s}+\sum_{k\notin I}\left[\left(s-\frac{1}{R_{k}}\right)-\left(s-s_{I}\right)\right]\phi_{k}=-\mu \frac{{\left(s-s_{I}\right)}^{2}}{s}+\sum_{k\notin I}\left(s_{I}-\frac{1}{R_{k}}\right)\phi_{k},$$

which is the displayed bound, since $s_{I}=1/R_{I}$. With $R_{k}\left(E_{I}\right)=R_{k}/R_{I}$,

$$\frac{1}{R_{I}}-\frac{1}{R_{k}}=\frac{R_{k}\left(E_{I}\right)-1}{R_{k}},$$

giving the equivalent form.

*Identification with the transversal closing condition.* For $z_{k}\neq 0$ define

$$\eta_{k}\left(x;E_{I}\right)=\left(\frac{1}{R_{I}}-\frac{1}{R_{k}}\right)\frac{\phi_{k}}{c_{k}^{T}z_{k}},$$

extended to $z_{k}=0$ by any value of the same sign; since $\pi_{k}\gg 0$ and $c_{k}\gg 0$, both $\phi_{k}$ and $c_{k}^{T}z_{k}$ are strictly positive whenever $z_{k}\neq 0$, so

$$\left(\frac{1}{R_{I}}-\frac{1}{R_{k}}\right)\phi_{k}=\eta_{k}\left(x;E_{I}\right)\;c_{k}^{T}z_{k},\;\mathrm{sign}\eta_{k}\left(x;E_{I}\right)=\mathrm{sign}\left(R_{k}\left(E_{I}\right)-1\right),$$

which is exactly the transversal term of (10) at $E_{I}$. The transversal Jacobian of the missing block *k* at $E_{I}$ is $J_{k}^{⊥}\left(E_{I}\right)=s_{I}w_{k}\pi_{k}^{T}-V_{k}$, with rank-one next-generation matrix $s_{I}w_{k}\pi_{k}^{T}V_{k}^{-1}$ of spectral radius $s_{I}\pi_{k}^{T}V_{k}^{-1}w_{k}=s_{I}R_{k}=R_{k}\left(E_{I}\right)$, so the Metzler next-generation threshold theorem gives

$$\mathrm{sign}s\left(J_{k}^{⊥}\left(E_{I}\right)\right)=\mathrm{sign}\left(R_{k}\left(E_{I}\right)-1\right)=\mathrm{sign}\eta_{k}\left(x;E_{I}\right).$$

Hence, $R_{k}\left(E_{I}\right)<1$ for every k∉I gives $\eta_{k}\left(x;E_{I}\right)<0$ whenever $z_{k}\neq 0$, which is precisely the transversal closing condition of Theorem 7 at $E_{I}$.

Together with the resident closing hypothesis, ${\dot{L}}_{I}\leq 0$ throughout the persistence class, with equality only where $z_{k}=0$ for every k∉I and the resident dissipation term vanishes; by hypothesis the largest invariant subset of this set is the resident equilibrium set selected on the face. If moreover the sublevel sets of $L_{I}$ in the persistence class are compact, LaSalle’s invariance principle gives convergence of every trajectory in the persistence class to that invariant set; when it reduces to $\left\{E_{I}\right\}$, this is global asymptotic stability of $E_{I}$ on the persistence class.    □

**Remark** **26** (Siphon calculus yields a Lyapunov Ansatz)**.** *Theorem 7 gives a common Lyapunov Ansatz indexed by a siphon σ and a resident equilibrium E. The resident blocks receive Perron–Volterra entropy terms and the transversal Frobenius blocks receive positive linear Perron functionals. The theorem reduces global convergence to the resident and transversal closing conditions. For the rank-one multi-strain models of [21], these conditions are obtained from the canonical normalization*

$$c_{k}^{T}=\frac{\pi_{k}^{T}V_{k}^{-1}}{R_{k}},$$

*which cancels the resident–invader coupling terms. Thus, that class provides a genuine block realization of the Ansatz.*

**Corollary** **21** (Affine scalar GLV specialization of the relay theorem)**.** *Consider the affine GLV system*

$$x^{\prime }=\mathrm{Diag}\left(x\right)\left(r+Mx\right),\;x\in R_{\geq 0}^{n},$$

*and let*

I⊆{1,…,n}

*be a resident support with equilibrium*

$$E_{I}\in F_{I}^{\circ }.$$

*Equivalently, the corresponding siphon is*

$$\sigma =I^{c}.$$

*Assume that there exists a≫0 such that*

$$Q:=\mathrm{Diag}\left(a\right)M+M^{T}\mathrm{Diag}\left(a\right)≺0.$$

*Then the Perron–Volterra function of Theorem 7 reduces to*

(12) $$\begin{array}{c} L_{I}\left(x\right)=\sum_{i\in I}a_{i}x_{i}^{*,I}G\;\left(\frac{x_{i}}{x_{i}^{*,I}}\right)+\sum_{j\notin I}a_{j}x_{j}. \end{array}$$

*Indeed, the singleton siphons of a scalar GLV system are one-dimensional transversal blocks, so their left Perron directions are scalar and their positive normalizations may be taken to be the coefficients $a_{i}$.*

*Put*

$$z_{i}=x_{i}-x_{i}^{*,I},\;i\in I,\;z_{j}=x_{j},\;j\notin I.$$

*Then*

(13) $$\begin{array}{c} {\dot{L}}_{I}=\frac{1}{2}z^{T}Qz+\sum_{j\notin I}a_{j}\lambda_{j|I}z_{j}, \end{array}$$

*where*

$$\lambda_{j|I}=f_{j}\left(E_{I}\right)$$

*is the transversal invasion exponent of the missing species j.*

*Consequently, if I is a relay sink,*

$$\lambda_{j|I}<0,\;j\notin I,$$

*then $L_{I}$ is a strict Lyapunov function away from $E_{I}$, and $E_{I}$ is globally asymptotically stable on*

$$\Omega_{I}=\left\{x\geq 0:\;x_{i}>0,\;i\in I\right\}.$$

*Thus, for affine scalar GLV systems, one diagonal-stability certificate a generates through the siphon formula of Theorem 7 the Lyapunov functions of all relay-sink faces simultaneously.*

**Proof.**  For scalar GLV systems, the minimal siphons are the singleton coordinates, and Proposition 2 gives

$$J_{\sigma }^{⊥}\left(E_{I}\right)=\mathrm{Diag}\left(\lambda_{j|I}:j\notin I\right).$$

Hence, every transversal Frobenius block is one-dimensional. Its left Perron direction is therefore the unique positive scalar direction, and choosing its positive normalization to be $a_{j}$ gives precisely (12). Thus, the entropy terms correspond to the inhabited singleton siphons i∈I, while the missing singleton siphons j∉I contribute the linear terms $a_{j}x_{j}$.

For i∈I, the resident equilibrium relation is

$$0=f_{i}\left(E_{I}\right)=r_{i}+\sum_{k\in I}M_{ik}x_{k}^{*,I}.$$

Therefore

$$f_{i}\left(x\right)=\sum_{k}M_{ik}z_{k}.$$

For a missing species j∉I,

$$\lambda_{j|I}=f_{j}\left(E_{I}\right)=r_{j}+\sum_{k\in I}M_{jk}x_{k}^{*,I},$$

and hence

$$f_{j}\left(x\right)=\lambda_{j|I}+\sum_{k}M_{jk}z_{k}.$$

Differentiating (12) gives

$${\dot{L}}_{I}=\sum_{i\in I}a_{i}z_{i}f_{i}\left(x\right)+\sum_{j\notin I}a_{j}z_{j}f_{j}\left(x\right).$$

Substitution of the preceding identities yields

$${\dot{L}}_{I}=\sum_{i,k}a_{i}M_{ik}z_{i}z_{k}+\sum_{j\notin I}a_{j}\lambda_{j|I}z_{j}=z^{T}\mathrm{Diag}\left(a\right)Mz+\sum_{j\notin I}a_{j}\lambda_{j|I}z_{j}.$$

Since the first term is a scalar,

$$z^{T}\mathrm{Diag}\left(a\right)Mz=\frac{1}{2}z^{T}\left(\mathrm{Diag}\left(a\right)M+M^{T}\mathrm{Diag}\left(a\right)\right)z=\frac{1}{2}z^{T}Qz,$$

which proves (13).

This also directly verifies the hypotheses of Theorem 7: the quadratic term

$$D_{I}\left(z\right)=\frac{1}{2}z^{T}Qz$$

is strictly negative for z≠0, while the coefficient of the linear term associated with a missing singleton siphon is exactly its transversal invasion exponent

$$\eta_{j|I}=\lambda_{j|I}.$$

If *I* is a relay sink, then

$$\lambda_{j|I}<0\;\left(j\notin I\right),$$

so

$${\dot{L}}_{I}<0\;\left(x\neq E_{I}\right).$$

Finally, the entropy terms in (12) diverge when any resident coordinate tends to 0 or to +∞, while the positive linear terms diverge when any missing coordinate tends to +∞. Thus, its sublevel sets in $\Omega_{I}$ are compact. The conclusion now follows either directly from LaSalle’s invariance principle or from Theorem 7.    □

**Remark** **27** (Related work on invasion graphs [RelayLit])**.** *The relay graph is, in substance, the invasion graph of Hofbauer and Schreiber [23], whose permanence criteria are formulated through invasion rates at boundary attractors, and whose structural stability for Lotka–Volterra systems, together with a description of the global attractor, is studied by Almaraz, Kalita, Langa and Soler–Toscano [24]. Those results establish which supports are reachable and when the system is permanent. In the affine scalar specialization, Corollary 21 adds a certificate: one weight vector a, valid simultaneously on every face, that converts each noninvadable support into a globally attracting equilibrium on its own persistence class, through the exact facewise identity ${\dot{L}}_{I}=\frac{1}{2}z^{T}Qz+\sum_{j\notin I}a_{j}\lambda_{j|I}z_{j}$. No comparison against the primary statements of those papers has been carried out here, so what is claimed is a difference of formulation, not of priority.*

**Remark** **28.** *Corollary 21, as the affine scalar specialization of Theorem 7, answers Section 6’s question precisely:* every relay sink—not just the interior equilibrium—is globally attracting on its own persistence class whenever M admits a single diagonally-stabilizing weight vector a, and the relay graph’s structure (which supports are sinks) together with that one certificate a determines the global dynamics completely. This subsumes Theorem 8 as the n=2 case (with the specific choice $a=(b_{21},b_{12})$, which is one interior point of the explicit interval of valid diagonal stabilizers described by Theorem 5). Two restrictions are inherent, not incidental: existence of a uniform a working for all faces simultaneously is itself the diagonal-stability property of the full matrix M, which can fail even when each face individually admits its own (different) weight, since a shared a must lie in the intersection of the (two-dimensional) admissible intervals of every pairwise face at once, and that intersection can be empty even when no single interval is; and the theorem is silent about supports that are not relay sinks (some missing species invades), where $L_{I}$ is not claimed to be, and by Remark 30 generally is not, a valid certificate. Whether the theorem extends to nonlinear f—with the relay graph still determining the attractor whenever local diagonal stability holds at every relay sink—is open, and Theorem 6 shows the naive extension (replacing M by $J=Df(x^{*})$ face by face) is not automatic even locally.

### 5.6. Restriction of Admissible Weights to Invariant Faces

The remark above already uses, informally, the fact that a shared weight must be admissible on every face at once. The following theorem makes this precise, and is the exact sense in which admissible weights *restrict* from the full system to any resident subsystem—answering, for the affine case, how the boundary geometry of Section 3 and the Volterra weights of Section 4 interact.

**Proposition** **7** (Admissible weights restrict to resident subsystems)**.** Let $f\left(x\right)=M\left(x-x^{*}\right)$, I⊆{1,…,n}, and let $M_{II}$ be the principal submatrix of M on I. If $E_{I}\in F_{I}^{\circ }$ is a positive equilibrium of the resident subsystem on I (necessarily governed by $x_{I}^{\prime }=\mathrm{diag}\left(x_{I}\right)M_{II}\left(x_{I}-E_{I}\right)$, regardless of $E_{I}$’s value), then for every $a\in W(x^{*})$ the restriction $a_{I}:={\left(a_{i}\right)}_{i\in I}$ satisfies $a_{I}\in W\left(E_{I}\right)$; if a additionally satisfies $\mathrm{diag}\left(a\right)M+M^{T}\mathrm{diag}\left(a\right)≺0$, then $\mathrm{diag}\left(a_{I}\right)M_{II}+M_{II}^{T}\mathrm{diag}\left(a_{I}\right)≺0$ as well.

**Proof.**  By Theorem 3, $a\in W(x^{*})$ means exactly $S:=\mathrm{diag}\left(a\right)M+M^{T}\mathrm{diag}\left(a\right)⪯0$; this condition depends only on *M*, not on $x^{*}$, so it is equally the exact admissibility condition for any other positive equilibrium of the same linear system. For i∈I, the resident dynamics is $x_{i}^{\prime }=x_{i}f_{i}{\left(x\right)|}_{x_{j}=0,\;j\notin I}=x_{i}\left(\sum_{j\in I}M_{ij}\left(x_{j}-x_{j}^{*}\right)-\sum_{j\notin I}M_{ij}x_{j}^{*}\right)$, affine in $x_{I}$ with linear part exactly $M_{II}$ (the constant term only shifts the equilibrium, from $x_{I}^{*}$ to whatever $E_{I}$ solves the resident equation); by Theorem 3 again, $a_{I}\in W\left(E_{I}\right)\Leftrightarrow \mathrm{diag}\left(a_{I}\right)M_{II}+M_{II}^{T}\mathrm{diag}\left(a_{I}\right)⪯0$. Now $S_{II}$, the principal submatrix of *S* on *I*, equals $\mathrm{diag}\left(a_{I}\right)M_{II}+M_{II}^{T}\mathrm{diag}\left(a_{I}\right)$ exactly, since restricting diag(a) and *M* to I×I commutes with the matrix operations defining *S* (verified directly, and numerically on the cooperative family’s asymmetric instance: the Perron weight’s restriction to {1,2} reproduces the {1,2} principal block of the full *S* exactly, with both negative eigenvalues {−13.00,−6.07}). Since a principal submatrix of a negative semidefinite (resp. negative definite) matrix is itself negative semidefinite (resp. negative definite)—$y^{T}Sy\leq 0$ for all *y* implies $y_{I}^{T}S_{II}y_{I}\leq 0$ for all $y_{I}$, by taking *y* supported on *I*—$S⪯0\Rightarrow S_{II}⪯0$, and likewise for ≺0.    □

**Remark** **29** (The converse fails, and the affine scalar corollary is the paper’s positive answer to it)**.** Proposition 7 is a one-way restriction, not a gluing theorem: individually admissible resident weights $a_{I},a_{J}$ on two faces I,J need not combine into a single $a\in W(x^{*})$, exactly because Remark 28 already observed that a shared certificate must lie in the intersection of every face’s admissible set, which can be empty even when no single face’s set is. Corollary 21 is the paper’s positive answer to the gluing question in the affine scalar case, by the opposite (top-down) route: rather than gluing separately-found resident weights, it starts from one a, valid for the full matrix M, and Proposition 7 shows that this single a automatically restricts correctly to every face at once—which is exactly why Corollary 21 can certify every relay sink simultaneously from one hypothesis. Whether a genuine bottom-up gluing criterion exists—conditions on a family of individually-admissible resident weights $\left\{a_{I}\right\}$ guaranteeing they extend to a common a—is left open.

### 5.7. What Remains Open

The following questions raised alongside this material are not resolved here, and we record them precisely rather than gesture at them.

- *Canonical weights beyond sign-stable Metzler matrices.* For every irreducible Metzler *M* with s(M)≤0, Theorem 4 gives the canonical Perron weight $a_{i}=\pi_{i}/w_{i}$ (Definition 14) and proves s(M)≤0 is *exactly* equivalent to diagonal semistability (Corollary 6)—recovering $a_{i}=1/x_{i}^{*}$ on the cyclic triangle at s(M)=0 (Corollary 24). This does *not* subsume Goh’s diagonal-dominance vector as a special case of the same formula: on the cooperative family’s asymmetric instance, the Perron weight, the inverse-formula weight of Corollary 12, and Goh’s *d* are verified to be three genuinely different, not merely rescaled, points of the same admissible cone (Remark 38). What remains open is which mixed-sign interaction matrices admit an analogous canonical construction. Candidate classes include matrices that are diagonally similar to Metzler matrices, sign-symmetric, eventually positive, dominated by a Hurwitz Metzler comparison matrix, or associated with a balanced signed graph; for each, whether a transformed Perron ratio produces an admissible Volterra weight is open. Also open, within the Metzler class itself: whether the growth functions can be made nonlinear while retaining a closed-form canonical weight (as opposed to only the local necessary condition of Proposition 4).
- *Intrinsic (graph/toric) characterization.* Whether admissibility of *a* can be read off the E-graph of (G,κ) directly—without first computing $J=Df(x^{*})$—analogous to how complex balance is read off weak reversibility and deficiency zero (Section 8), is open; for n=2, Theorem 5 already gives such a characterization in closed form (an explicit rational inequality in the matrix entries), but whether an analogous closed-form, graph-readable criterion exists for n≥3—where, by Remark 22, the admissible region may genuinely be a curved spectrahedron rather than a polyhedron—is open.
- *Complex balance and the weights.* An open problem is to identify a single algebraic quantity encoded uniformly by the weights across reversible, cooperative, cyclic and higher-order systems: Mechanism DD’s weights are literally Goh’s diagonal-dominance certificate and the raw SDP’s (Theorem 3) are a diagonal-stability certificate for the interaction matrix, two different classical objects rather than two instances of one.
- *Multistationarity and the relay graph.* Whether a change in the number of positive equilibria (Theorem 9) always accompanies a qualitative change in the relay graph remains open; the counterexample instance of Theorem 9 has a relay graph ($E_{1}$ invaded, $E_{2}$ a sink) that leaves the second interior sink’s existence undetermined. A natural problem is to augment the relay graph by a minimal scalar datum—e.g., the discriminant of the reduced equilibrium polynomial underlying Theorem 9—capable of recovering the full equilibrium partition.
- *CRNT equivalences and algorithms.* Formulating exact equivalence theorems between Volterra admissibility and persistence, ACR, or general embedded-graph realizability, and designing an algorithm that decides $W(x^{*})\neq ⌀$ beyond the affine class (where Corollary 16 already reduces it to a semidefinite feasibility problem), are left for future work.
- *Exact weights for the cyclic triangle.* Solving the semidefinite feasibility problem of Corollary 16 symbolically for the specific parameters of Section 9.1 would give the full normalized admissible region for that example, and settle whether its boundary has a rational or algebraic canonical point, rather than only the single interior weight already exhibited there.
- *Finite certification of differential diagonal stability.* For polynomial or generalized-polynomial GLV systems, find finite coefficient, graph-theoretic, sum-of-squares, or vertex conditions implying $A\;Df\left(x\right)+Df{\left(x\right)}^{T}A≺0$ on a prescribed convex invariant region Ω, so that Theorem 6 and its corollaries (Section 5.4) can be applied without an exhaustive search over Ω; determine classes of systems for which uniform diagonal stability on Ω can be decided by finitely many matrix inequalities, and whether Ω can always be shrunk from the whole orthant to a smaller invariant region containing the relevant trajectories, per Corollary 18.
- *Uniform face weights.* Characterizing, for a general interaction matrix *M*, when a single a>0 satisfies $\mathrm{diag}\left(a\right)M+M^{T}\mathrm{diag}\left(a\right)≺0$ would feed directly into Theorem 7, certifying every relay sink at once; graph-theoretic sufficient conditions for sign-symmetric, triangular, or competitive *M* are a natural target, generalizing Goh’s diagonal-dominance criterion (Corollary 5) beyond the cooperative case.

The cyclic-feedback instance of Section 4.1 motivates a further question: there, the strict margin $\delta^{*}$ of Corollary 3 vanishes exactly at the parameter value where M(K) loses Hurwitz stability through a Hopf bifurcation, but nothing in the argument for that specific family shows this coincidence is more than a numerical fact about it.

**Problem** **1** (Volterra margin versus the Hurwitz–Hopf boundary [delta-hurwitz-hopf])**.** *Determine structural classes of affine GLV families*

$$f_{\theta }\left(x\right)=M\left(\theta \right)\left(x-x^{*}\left(\theta \right)\right)$$

for which strict Volterra admissibility, in the sense of the margin $\delta^{*}$ of Corollary 3, is equivalent to Hurwitz stability:

$$\delta^{*}\left(\theta \right)>0\Leftrightarrow M\left(\theta \right)\;\mathrm{is}\;\mathrm{Hurwitz}.$$

*For such classes, the boundary*

$$\delta^{*}\left(\theta \right)=0$$

*is exactly the Hurwitz stability boundary. Determine, moreover, conditions under which a regular portion of this common boundary is a Hopf locus, i.e., the loss of stability occurs through a simple pair ±iω, ω>0, while all remaining eigenvalues stay in the open left half-plane and the usual transversality/nondegeneracy hypotheses hold. In particular, characterize the matrix structures for which the coincidence observed in the cyclic-feedback example above is forced rather than accidental.*

## 6. Boundary Equilibria and Competitive Exclusion in Two Examples

This section illustrates the intrinsic theory of Section 3, Section 4 and Section 5 on two representative families; no Euclidean-graph realization is used anywhere in it. The first is an affine competition model, where the admissible-weight theory yields a complete global exclusion/coexistence partition. The second adds higher-order interactions, and shows how nonlinear terms create multistationarity while leaving the boundary relay analysis unchanged.

The question both are chosen to answer is whether the relay graph, together with one admissible weight, determines the global attractor. In the parameter sectors originally considered by Rojas La Luz, Yu and Craciun, the question is empty: boundary equilibria exist, but every missing species invades ($\lambda_{2|1},\lambda_{1|2}>0$), so no nontrivial exclusion chamber lies inside those sign assumptions and the relay graph sends both boundary residents to coexistence. A genuine exclusion partition appears only once negative cross-interactions are allowed, which is what the two families below do.

### 6.1. The Affine Two-Species Family

Consider the signed affine family

(14) $$\begin{array}{cc} x^{\prime } & =x(r_{1}-a_{11}x+q_{12}y),\\ y^{\prime } & =y(r_{2}+q_{21}x-a_{22}y), \end{array}\;a_{11},a_{22}>0,$$

with the interaction coefficients $q_{12},q_{21}\in R$ free to have either sign. This single family contains several standard regimes:

$$\begin{array}{cc} \left(q_{12},q_{21}\right) & \mathrm{interaction}\;\mathrm{type}\\ q_{12},q_{21}>0 & \mathrm{cooperative}\;\mathrm{GLV}\\ q_{12},q_{21}<0 & \mathrm{competitive}\;\mathrm{GLV}\\ q_{12}q_{21}<0 & \mathrm{predator}-\mathrm{prey}/\mathrm{mixed}\;\mathrm{interaction} \end{array}$$

The cooperative family of Section 9.2 is the two-dimensional specialization with $q_{12},q_{21}\geq 0$; its symmetric numerical instance is recovered at $r_{1}=r_{2}=1$, $a_{11}=a_{22}=3$, $q_{12}=q_{21}=1$. The present extension is therefore not a different model: it is the same affine GLV family with the signs of the interactions left free. We work out the equilibria and invasion exponents for arbitrary $q_{12},q_{21}$ before specializing.

**Definition** **17** (Equilibria and invasion data of (14) [Equ])**.** *$E_{0}=\left(0,0\right)$; $E_{1}=\left(r_{1}/a_{11},0\right)$, positive iff $r_{1}>0$; $E_{2}=\left(0,r_{2}/a_{22}\right)$, positive iff $r_{2}>0$; and, with $\Delta_{q}:=a_{11}a_{22}-q_{12}q_{21}$ and $\Delta_{q}\neq 0$,*

$$E_{12}=\left(x^{*},y^{*}\right),\;x^{*}=\frac{a_{22}r_{1}+q_{12}r_{2}}{\Delta_{q}},\;y^{*}=\frac{a_{11}r_{2}+q_{21}r_{1}}{\Delta_{q}}.$$

*The invasion exponents are*

$$\lambda_{1|0}=r_{1},\;\lambda_{2|0}=r_{2},\;\lambda_{2|1}=r_{2}+\frac{q_{21}r_{1}}{a_{11}},\;\lambda_{1|2}=r_{1}+\frac{q_{12}r_{2}}{a_{22}}.$$

**Lemma** **3** (Positivity via the invasion exponents)**.** $x^{*}=a_{22}\lambda_{1|2}/\Delta_{q}$ and $y^{*}=a_{11}\lambda_{2|1}/\Delta_{q}$. In particular, if $\Delta_{q}>0$, then $E_{12}\in R_{>0}^{2}$ if and only if $\lambda_{2|1}>0$ and $\lambda_{1|2}>0$.

**Proof.**  Direct substitution: $a_{22}\lambda_{1|2}=a_{22}r_{1}+q_{12}r_{2}=\Delta_{q}x^{*}$, and symmetrically for $y^{*}$. Since $a_{11},a_{22}>0$, when $\Delta_{q}>0$ the sign of $x^{*}$ (resp. $y^{*}$) equals the sign of $\lambda_{1|2}$ (resp. $\lambda_{2|1}$).    □

#### 6.1.1. The Cooperative Sector Is Degenerate

Assume $r_{1},r_{2}>0$, $q_{12},q_{21}\geq 0$—the hypotheses of Section 9.2. Then $\lambda_{2|1}=r_{2}+q_{21}r_{1}/a_{11}>0$ and $\lambda_{1|2}=r_{1}+q_{12}r_{2}/a_{22}>0$ unconditionally: every term is nonnegative and $r_{1},r_{2}>0$ makes each sum strictly positive. Both axial equilibria, whenever they exist ($r_{1}>0$ and $r_{2}>0$, given), are therefore transversally unstable, and the relay graph is

$$E_{0}\to E_{1},\;E_{0}\to E_{2},\;E_{1}\to E_{12},\;E_{2}\to E_{12}.$$

Under the diagonal-dominance hypothesis of Corollary 5 (∃d>0 with $d_{1}\left(-a_{11}\right)+d_{2}q_{21}\leq 0$ and $d_{1}q_{12}+d_{2}\left(-a_{22}\right)\leq 0$), multiplying these two inequalities (both sides nonnegative, since $d_{2}q_{21}\leq d_{1}a_{11}$ and $d_{1}q_{12}\leq d_{2}a_{22}$ with all quantities ≥0) gives $q_{12}q_{21}\leq a_{11}a_{22}$, i.e., $\Delta_{q}\geq 0$; since the numerators $a_{22}r_{1}+q_{12}r_{2}$ and $a_{11}r_{2}+q_{21}r_{1}$ of $x^{*},y^{*}$ are already strictly positive, $\Delta_{q}>0$ makes $E_{12}$ the unique positive equilibrium (Lemma 3 confirms this from the invasion side too, since both exponents are positive):

$$\begin{array}{c} \mathrm{for}\;\mathrm{the}\;\mathrm{cooperative}\;\mathrm{parameter}\;\sec\mathrm{tor},\;\mathrm{the}\;\mathrm{interior}\;\mathrm{equilibrium}\;\mathrm{is}\;\mathrm{the}\;\mathrm{only}\;\mathrm{possible}\;\mathrm{attractor}\;\mathrm{from}\;R_{>0}^{2}. \end{array}$$

The boundary equilibria exist and are listed above, but do not compete with $E_{12}$: every missing species invades. This is why the interior complex-balance analysis of Section 9.2 already settles the positive dynamics in this sector, even without listing the boundary equilibria.

**Remark** **30** (No globally decreasing boundary function [coop-boundary-lyap])**.** Let I∈{{1},{2}} and j∉I with $\lambda_{j|I}>0$. No face-adapted function $L_{I}\left(x\right)=a_{i}x_{i}^{*}G\left(x_{i}/x_{i}^{*}\right)+c_{j}x_{j}$ ($a_{i},c_{j}>0$) can satisfy ${\dot{L}}_{I}\leq 0$ arbitrarily close to $E_{I}$ with $x_{j}>0$. This is the identity of Theorem 7 read at $z_{i}=0$, $z_{j}=\varepsilon$, where it reduces to

$${\dot{L}}_{I}=\frac{1}{2}Q_{jj}\varepsilon^{2}+c_{j}\lambda_{j|I}\varepsilon ,$$

positive for all small ε>0 whenever $\lambda_{j|I}>0$—and note that no definiteness of Q is used, only the identity itself. The same computation covers the higher-order family of Section 6.2, since there too $f_{j}$ is exactly affine in $x_{j}$ at fixed $x_{i}=x_{i}^{*}$, so the expansion in ε terminates and carries no remainder.

This is the Lyapunov counterpart of transversal instability: in the cooperative sector, Remark 30 applies with $\lambda_{2|1},\lambda_{1|2}>0$, so *no* face-adapted function centered at $E_{1}$ or $E_{2}$ can serve as a global Lyapunov function—consistent with $E_{12}$ being the only attractor.

#### 6.1.2. The Competitive Sector: Weak Competition Gives a Complete Partition

Now specialize to $q_{12}=-b_{12}$, $q_{21}=-b_{21}$, $b_{12},b_{21}>0$, $\Delta :=a_{11}a_{22}-b_{12}b_{21}$, and

$$\lambda_{2|1}=r_{2}-\frac{b_{21}r_{1}}{a_{11}},\;\lambda_{1|2}=r_{1}-\frac{b_{12}r_{2}}{a_{22}}.$$

The central question this sub-family answers is stronger than the one-equilibrium question of Section 9:

$$\begin{array}{c} \mathrm{Can}\;\mathrm{one}\;\mathrm{family}\;\mathrm{of}\;\mathrm{face}-\mathrm{adapted}\;\mathrm{Volterra}\;\mathrm{functions}\;\mathrm{prove}\;\mathrm{the}\;\mathrm{complete}\;\mathrm{boundary}/\mathrm{interior}\;\mathrm{exclusion}\;\mathrm{partition}? \end{array}$$

**Theorem** **8** (Weak competition: the exclusion/coexistence partition [weak-competition])**.** *Suppose Δ>0. Then $\lambda_{2|1}<0$ and $\lambda_{1|2}<0$ cannot hold simultaneously, and:*

*(a)*  *if $\lambda_{2|1}<0$ (so $\lambda_{1|2}>0$ necessarily), $E_{1}$ is globally asymptotically stable on $\Omega_{1}^{+}=\left\{\left(x,y\right)\in R_{\geq 0}^{2}:x>0\right\}$;*
*(b)*  *symmetrically, if $\lambda_{1|2}<0$, $E_{2}$ is globally asymptotically stable on $\Omega_{2}^{+}=\left\{\left(x,y\right)\in R_{\geq 0}^{2}:y>0\right\}$;*
*(c)*  *if $\lambda_{2|1}>0$ and $\lambda_{1|2}>0$, then $E_{12}\in R_{>0}^{2}$ (Lemma 3) and is globally asymptotically stable on $R_{>0}^{2}$.*

*Every case is certified by the* same *weighted matrix $Q=(\begin{array}{cc} b_{21}a_{11} & b_{12}b_{21}\\ b_{12}b_{21} & b_{12}a_{22} \end{array})$, positive definite exactly when Δ>0.*

**Proof.**  Take $L_{12}\left(x,y\right)=b_{21}x^{*}G\left(x/x^{*}\right)+b_{12}y^{*}G\left(y/y^{*}\right)$ for (c), and $L_{1}\left(x,y\right)=b_{21}x_{1}^{*}G\left(x/x_{1}^{*}\right)+b_{12}y$, $L_{2}\left(x,y\right)=b_{21}x+b_{12}y_{2}^{*}G\left(y/y_{2}^{*}\right)$ for (a), (b)—note the weights are the *cross* interaction coefficients, $b_{21}$ on the *x*-term and $b_{12}$ on the *y*-term, not a free pair to be solved for. Exact computation (verified symbolically) gives, with $u=x-x^{*}$, $v=y-y^{*}$ (resp. $u=x-x_{1}^{*}$; $v=y-y_{2}^{*}$),

$${\dot{L}}_{12}=-\left(\begin{array}{cc} u & v \end{array}\right)Q\left(\begin{array}{c} u\\ v \end{array}\right),\;{\dot{L}}_{1}=-\left(\begin{array}{cc} u & y \end{array}\right)Q\left(\begin{array}{c} u\\ y \end{array}\right)+b_{12}\lambda_{2|1}y,\;{\dot{L}}_{2}=-\left(\begin{array}{cc} x & v \end{array}\right)Q\left(\begin{array}{c} x\\ v \end{array}\right)+b_{21}\lambda_{1|2}x,$$

with no remainder in any case, since *f* is affine. $Q_{11}=b_{21}a_{11}>0$ and $\det Q=b_{12}b_{21}a_{11}a_{22}-{\left(b_{12}b_{21}\right)}^{2}=b_{12}b_{21}\Delta$, so Q≻0 exactly when Δ>0 (Sylvester’s criterion). Hence, the quadratic form is <0 away from the origin in each case: for (c), ${\dot{L}}_{12}<0$ for (u,v)≠(0,0), i.e., away from $E_{12}$, on all of $R_{>0}^{2}$ (no sign restriction on u,v was used). For (a), ${\dot{L}}_{1}\leq -\left(\begin{array}{cc} u & y \end{array}\right)Q\left(\begin{array}{c} u\\ y \end{array}\right)\leq 0$ once $\lambda_{2|1}\leq 0$ and y≥0, with equality forcing both the (positive-definite) quadratic term and $b_{12}\lambda_{2|1}y$ to vanish, i.e., u=y=0: equality only at $E_{1}$. (b) is symmetric. Simultaneity of $\lambda_{2|1}<0,\lambda_{1|2}<0$ under Δ>0 is excluded exactly as claimed: $\lambda_{2|1}<0\Rightarrow b_{21}r_{1}>a_{11}r_{2}$ and $\lambda_{1|2}<0\Rightarrow b_{12}r_{2}>a_{22}r_{1}$ would multiply to $b_{12}b_{21}r_{1}r_{2}>a_{11}a_{22}r_{1}r_{2}$, i.e., $b_{12}b_{21}>a_{11}a_{22}$, contradicting Δ>0.

It remains to upgrade $\dot{L}\leq 0$ to global *asymptotic* stability. Boundedness: $x^{\prime }\leq x\left(r_{1}-a_{11}x\right)$ (dropping the nonpositive competition term $-b_{12}xy\leq 0$), so ${lim sup}_{t}x\left(t\right)\leq r_{1}/a_{11}$ by the standard logistic comparison, and likewise for *y*; every forward trajectory therefore eventually enters a compact positively invariant box. On that box, ${\dot{L}}_{I}\leq 0$ with $\left\{{\dot{L}}_{I}=0\right\}=\left\{E_{I}\right\}$, so LaSalle’s invariance principle gives convergence of every trajectory in the stated domain to $E_{I}$, i.e., genuine global asymptotic stability, not merely a nonincreasing Lyapunov value.    □

**Remark** **31.** *The weight choice here is not unique—Theorem 3 characterizes the full cone $W(E_{12})$, and the discriminant- optimized weights used in an earlier draft of this section also work—but the cross-weights $\left(b_{21},b_{12}\right)$ are canonical: they require no optimization step; they are the same triple $\left(b_{21},b_{12}\right)$ for all three candidates $L_{1},L_{2},L_{12}$; and the* same *matrix Q controls every derivative, with the only difference between cases being which linear invasion term, $b_{12}\lambda_{2|1}y$ or $b_{21}\lambda_{1|2}x$, is present. This is the SDP of Theorem 3 solved in closed form: the family of Volterra functions does not merely prove GAS of one interior equilibrium, it converts the scalar relay/invasion data of Section 6 into a complete global exclusion/coexistence partition; the three cases are collected in Table 1.*

#### 6.1.3. Strong Competition: Bistability, Not Exclusion

If Δ<0 one recovers the classical bistable competitive Lotka–Volterra picture [25]: $\lambda_{2|1}$ and $\lambda_{1|2}$ can be simultaneously negative (as at $a_{11}=a_{22}=1$, $b_{12}=b_{21}=3$, $r_{1}=r_{2}=1$, where Δ=−8 and $\lambda_{2|1}=\lambda_{1|2}=-2$), both axial equilibria are then locally stable, and the interior equilibrium is a saddle whose stable manifold separates their basins. This is a basin partition, not a parameter partition: which axial equilibrium is reached depends on the initial condition. The cross-weight functions of Theorem 8 do not extend to it—$\det Q=b_{12}b_{21}\Delta <0$, so *Q* is indefinite—and no global Lyapunov function of any form can exist, since two attractors do.

#### 6.1.4. Conic Realization for the Planar Competitive Family

Theorem 8 certifies all three regimes with one matrix *Q* built from the *cross* coefficients: the weight is $a=(b_{21},b_{12})$, “not a free pair to be solved for”. Theorem 5 computes, for the same family, the entire set of weights that would have worked. Comparing the two answers the question that the first leaves open—why *that* pair—and the answer is sharper than admissibility.

In the competitive sector $q_{12}=-b_{12}$, $q_{21}=-b_{21}$ with $b_{12},b_{21}>0$, and weak competition is $\Delta =a_{11}a_{22}-b_{12}b_{21}>0$. Theorem 5(ii) applies, and the admissible cone is the angular sector $r=a_{2}/a_{1}\in \left[r_{-},r_{+}\right]$ with

$$r_{\pm }=\frac{2a_{11}a_{22}-b_{12}b_{21}\pm 2\sqrt{a_{11}a_{22}\Delta }}{b_{21}^{2}}.$$

By Corollary 15, the geometric mean of the two extreme rays is $|q_{12}/q_{21}|$, which here is $b_{12}/b_{21}$: the cross-coefficient weight $a=(b_{21},b_{12})$ of Theorem 8 is therefore not merely an interior point of $W(E_{12})$ but the center of the cone, in the multiplicative sense in which a cone of ratios has a center.

This is a statement the Lyapunov computation alone cannot make. Solving ${\dot{L}}_{a}\leq 0$ produces *a* weight; the conic description produces the set of all of them, and only against that set can a particular choice be recognized as canonical. Evaluating $r_{\pm }$ exactly on the instances $\left(a_{11},a_{22},b_{12},b_{21}\right)=\left(1,1,0.5,0.5\right)$, (1,2,0.4,1.1) and (2,3,1.5,0.8) gives admissible ratio intervals [0.0718,13.9282], [0.0226,5.8617] and [0.1045,33.6455]. The intervals are wide—in the first the admissible ratios span a factor of 194—which is the quantitative form of the statement that Theorem 8’s weight is one choice among many. It is nonetheless the midpoint of that span on a logarithmic scale, in every instance, by Corollary 15.

**Strong competition.** When Δ<0 the same computation returns the opposite verdict, and returns it as a certificate rather than as a failure to find weights. For $a_{11}=a_{22}=1$, $b_{12}=1.2$, $b_{21}=1.1$ (Δ=−0.32) the discriminant of Ψ is negative, so Ψ>0 everywhere and Theorem 5(i) gives $W(E_{12})=⌀$; minimizing $\lambda_{\max}$ over the simplex confirms it numerically, with minimum +0.1486>0, and for $b_{12}=2$, $b_{21}=3$ (Δ=−5) the minimum is +1.2361. No Volterra function of the form $L_{a}$ exists in the bistable sector—consistent with Section 6.1.3, where the conclusion is bistability rather than exclusion, so no global Lyapunov function of any form can exist. The conic formulation detects the obstruction directly from *M*, without having to analyze the phase portrait.

Numerically the cone is located by minimizing $\lambda_{\max}$ over the simplex, whose optimal value is ≤0 exactly when W≠⌀, and which is global since $\lambda_{\max}$ is convex in *a*.

### 6.2. The Higher-Order-Interaction Family

Recall from Section 9.3 the higher-order-interaction pair

(15) $$\begin{array}{cc} x^{\prime } & =x(r_{1}-a_{11}x+a_{12}y-b_{1}xy),\\ y^{\prime } & =y(r_{2}+a_{21}x-a_{22}y-b_{2}xy). \end{array}$$

Since the $b_{i}xy$ terms vanish whenever x=0 or y=0, the axial data is formally identical to the affine case: $E_{1}=\left(r_{1}/a_{11},0\right)$, $E_{2}=\left(0,r_{2}/a_{22}\right)$, $\lambda_{2|1}=r_{2}+a_{21}r_{1}/a_{11}$, $\lambda_{1|2}=r_{1}+a_{12}r_{2}/a_{22}$.

#### 6.2.1. The Original Sector Is Degenerate, Exactly as in the Affine Case

Under $r_{1},r_{2}>0$, $a_{12},a_{21}\geq 0$ (the hypotheses of Section 9.3), $\lambda_{2|1}>0$ and $\lambda_{1|2}>0$ unconditionally, by the identical argument of Section 6.1.1. Hence, $E_{1}\to E_{12}$, $E_{2}\to E_{12}$ whenever $E_{12}$ exists, and Remark 30 (which used only the linear-in-$x_{j}$ leading term, valid for any GLV system regardless of higher-order terms) applies verbatim: no face-adapted function centered at $E_{1}$ or $E_{2}$ can be globally decreasing. The original parameter sector of Rojas La Luz, Yu and Craciun [4] yields no nontrivial exclusion partition.

#### 6.2.2. Signed Extension: Existence and Multistationarity

Introduce signed cross-interactions,

(16) $$\begin{array}{cc} x^{\prime } & =x(r_{1}-a_{11}x+q_{12}y-b_{1}xy),\\ y^{\prime } & =y(r_{2}+q_{21}x-a_{22}y-b_{2}xy), \end{array}\;q_{12},q_{21}\in R,$$

recovering (15) at $q_{12}=a_{12}\geq 0$, $q_{21}=a_{21}\geq 0$. Axial equilibria and invasion exponents keep the same formulas as (14) (the $b_{i}$ terms still vanish on the axes).

**Theorem** **9** (Higher-order interactions [hoi-elim]).

*(i)*  *Eliminating y from $r_{1}-a_{11}x+q_{12}y-b_{1}xy=0$ via $y=\left(a_{11}x-r_{1}\right)/\left(q_{12}-b_{1}x\right)$ and substituting into the second equilibrium equation gives, after clearing denominators,*
  $$Ax^{2}+Bx+C=0,\;A=a_{11}b_{2}+b_{1}q_{21},\;B=\Delta_{q}+b_{1}r_{2}-b_{2}r_{1},\;C=-\left(a_{22}r_{1}+q_{12}r_{2}\right),$$
  *where $\Delta_{q}=a_{11}a_{22}-q_{12}q_{21}$ as before. At $b_{1}=b_{2}=0$ (A=0) this reduces to the affine case’s linear equation $\Delta_{q}x=a_{22}r_{1}+q_{12}r_{2}$.*
*(ii)*  *If $r_{1},r_{2}>0$ and $q_{12},q_{21}\geq 0$ (original sector), then C<0; if in addition A>0, that quadratic has a unique positive root, and it is automatically real (a quadratic with real coefficients and negative root-product cannot have complex roots).*
*(iii)*  *If $q_{12},q_{21}$ are allowed negative, C can become positive, and two strictly positive roots—both with positive corresponding y, hence two genuine positive equilibria of (16)—can occur: on $a_{11}=a_{22}=1$, $b_{1}=0.144$, $b_{2}=1.701$, $r_{1}=4.84$, $r_{2}=4.94$, $q_{12}=-3.359$, $q_{21}=-0.733$, the system has interior equilibria (2.067,0.758) (a saddle, eigenvalues {−6.31,+0.59}) and (3.563,0.330) (a sink, eigenvalues {−5.56,−0.50}), together with $E_{1}=\left(4.84,0\right)$ (a saddle, $\lambda_{2|1}=1.39>0$) and $E_{2}=\left(0,4.94\right)$ (a* second *sink, eigenvalues {−11.75,−4.94}, consistent with $\lambda_{1|2}=-11.75<0$).*

**Proof.**  (i) Direct symbolic elimination: the resultant of the two equilibrium equations with respect to *y* is exactly this quadratic. (ii) $C=-(a_{22}r_{1}+q_{12}r_{2})<0$ when $q_{12}\geq 0,r_{1},r_{2}>0$; for a real quadratic, product of roots C/A<0 forces one positive and one non-positive real root (complex conjugate roots would have nonnegative product), giving uniqueness of the positive root. The multistationarity instance of (iii) was found by randomized search over the signed sector and verified directly: both roots of $Ax^{2}+Bx+C=0$ are real, positive, give positive *y* via the elimination formula, satisfy the original nonlinear system exactly, and their Jacobian eigenvalues were computed directly.    □

This is a genuinely new phenomenon absent from the affine family: two stable attractors (here $E_{2}$ and one interior equilibrium) can coexist even though the parameter values are perfectly ordinary, separated by two saddles ($E_{1}$ and the other interior equilibrium)—a “backward”-type boundary relay, in the sense that which attractor is reached depends on more than the signs of $\lambda_{2|1},\lambda_{1|2}$ alone (both signs here are compatible with $E_{2}$ attracting, but do not rule out the coexisting interior sink).

#### 6.2.3. Lyapunov Analysis: What Survives the Nonlinearity

For a face-adapted candidate $L_{I}\left(x\right)=\sum_{i\in I}a_{i}x_{i}^{*}G\left(x_{i}/x_{i}^{*}\right)+\sum_{j\notin I}c_{j}x_{j}$ on (16), the general decomposition of Section 4.1 still gives, for I={1},

$${\dot{L}}_{1}=-a_{1}a_{11}u^{2}-c_{2}a_{22}y^{2}+\left(a_{1}q_{12}+c_{2}q_{21}\right)uy+c_{2}\lambda_{2|1}y-a_{1}b_{1}u\cdot xy-c_{2}b_{2}y\cdot xy,$$

where the last two terms (absent in Section 6.1) are the genuine higher-order remainder, cubic in (u,y) jointly. Unlike the affine case, this remainder does not have a fixed sign under the complex-balance sign condition of the original example: it is positive for some (u,y) in the positive quadrant and negative for others (e.g., at fixed small y>0, the sign of $-a_{1}b_{1}u\cdot xy$ flips with the sign of $u=x-x_{1}^{*}$). Consequently, the exact global argument of Theorem 8 does *not* extend verbatim: the necessary condition of Proposition 4 (diagonal stability of the Jacobian at each equilibrium) remains available and was exactly what certified $\dot{V}\leq 0$ numerically in Section 9.3’s original, single-equilibrium instance, but a general sufficient theorem covering the signed, possibly-multistationary sector is not established here. Whether the unit-weight Volterra function of Section 9.3 extends to an open signed parameter region, and whether face-adapted functions at $E_{1},E_{2}$ separately certify exclusion whenever the system has a unique interior equilibrium, are left as the concrete open problems this sub-family poses; Table 2 summarises this sector.

## 7. Euclidean-Graph Realizations and Kirchhoff Matrices

Everything up to this point depends only on the GLV vector field, through its coordinate-face/siphon geometry, transversal invasion data and the Volterra admissibility problem. From here on, an additional structure is imposed on the same ODE: an E-graph realization of *f*, which the vector field does not itself determine (Remark 32). This realization introduces the vertex and Kirchhoff matrices and the stoichiometric subspace, which produce the compatibility manifolds and permit comparison with complex balance.

**Definition** **18** (Euclidean-graph realization [Erealiz])**.** *An* E-graph realization *of f is a finite directed graph G=(V,E) with vertex set*

$$V=\left\{y_{1},\ldots ,y_{m}\right\}\subset R^{n}$$

*and positive edge weights $\kappa_{ij}>0$, (i,j)∈E, such that*

$$f\left(x\right)=\sum_{(i,j)\in E}\kappa_{ij}\;x^{y_{i}}\;\left(y_{j}-y_{i}\right).$$

*Writing $Y=(y_{1}\;\cdots \;y_{m})$ for the n×m vertex matrix, $x^{Y}$ for the vector with entries ${\left(x^{Y}\right)}_{i}=x^{y_{i}}$, and $A_{\kappa }$ for the m×m* column Kirchhoff matrix

$${\left(A_{\kappa }\right)}_{ji}=\kappa_{ij}\;\;\left(i\neq j\right),\;{\left(A_{\kappa }\right)}_{ii}=-\sum_{j\neq i}\kappa_{ij},$$

*the GLV system generated by (G,κ) is*

(17) $$x^{\prime }=\mathrm{diag}\left(x\right)\;YA_{\kappa }x^{Y}.$$

*Setting*

$$S\left(G\right)=\mathrm{span}\{y_{j}-y_{i}:\left(i,j\right)\in E\},$$

*the columns of $A_{\kappa }$ sum to zero, $YA_{\kappa }x^{Y}$ expands term by term to the displayed edge sum, and Im(YAκ)⊆S(G): consequently every trajectory of (17) satisfies $\frac{d}{dt}\log x\in S\left(G\right)$, the source of the first integrals and compatibility manifolds of Section 8.*

**Remark** **32** (Nonuniqueness of the realization [RealNU])**.** *An E-graph realization is not determined by f: the same vector field admits different choices of vertices, edges, and weights, so S(G), weak reversibility, deficiency, and complex balance are attributes of the pair (G,κ), not of the vector field, unless independently proved invariant. Throughout Part II one E-graph realization is fixed, and S=S(G) refers to it.*

## 8. Compatibility Manifolds in the Original Variables

Fix throughout an E-graph realization (G,κ) of the GLV vector field, as in Definition 18,

(18) $$x^{\prime }=\mathrm{diag}\left(x\right)\;YA_{\kappa }x^{Y},\;x\in R_{>0}^{n}.$$

Its stoichiometric subspace is

(19) $$S=S\left(G\right)=\mathrm{span}\{y_{j}-y_{i}:\left(i,j\right)\in E\}.$$

Notice that *S* depends on the chosen E-graph *G*, although not on the edge weights κ. Since a GLV vector field may admit several E-graph realizations, the compatibility manifolds below belong to the chosen realization, not to the vector field alone.

Rojas La Luz, Yu and Craciun [4] introduce these invariant sets through the logarithmic variables ξ=logx, where they are affine translates of *S*. We record first their equivalent description directly in the original positive variables.

### 8.1. First Integrals and Compatibility Manifolds

**Proposition** **8** (First integrals and compatibility manifolds of a fixed realization)**.** *For every $w\in S^{⊥}$,*

$$H_{w}\left(x\right)=w^{T}\log x$$

*is a first integral of (18). Consequently, for every $x^{0}\in R_{>0}^{n}$,*

(20) $$C\left(x^{0}\right):=\left\{x>0:H_{w}\left(x\right)=H_{w}\left(x^{0}\right)\;\forall \;w\in S^{⊥}\right\}$$

*is positively invariant and admits the equivalent descriptions*

(21) $$\begin{array}{c} C\left(x^{0}\right)=\left\{x>0:\log x-\log x^{0}\in S\right\}=\exp\left(\log x^{0}+S\right). \end{array}$$

*In particular, $C(x^{0})$ is a smooth embedded manifold of dimension dimS.*

**Proof.**  Since

$$\frac{d}{dt}\log x=\mathrm{diag}{\left(x\right)}^{-1}x^{\prime }=YA_{\kappa }x^{Y},$$

we have

$$\frac{d}{dt}H_{w}\left(x\right)=w^{T}YA_{\kappa }x^{Y}.$$

Every column of $YA_{\kappa }$ belongs to *S*. Indeed, with the column Kirchhoff convention, the column corresponding to the vertex $y_{i}$ is

$$\sum_{j:(i,j)\in E}\kappa_{ij}\left(y_{j}-y_{i}\right),$$

and is therefore a linear combination of edge vectors. Hence

Im(YAκ)⊆S.

For $w\in S^{⊥}$ this gives

$$w^{T}YA_{\kappa }=0,$$

and therefore ${\dot{H}}_{w}=0$.

Now *x* belongs to $C(x^{0})$ precisely when

$$w^{T}\left(\log x-\log x^{0}\right)=0\;\forall \;w\in S^{⊥}.$$

Since ${\left(S^{⊥}\right)}^{⊥}=S$, this is equivalent to

$$\log x-\log x^{0}\in S,$$

which proves (21). Finally, the componentwise exponential map is a diffeomorphism from $R^{n}$ onto $R_{>0}^{n}$, so the image of the affine space $\log x^{0}+S$ is a smooth embedded manifold of dimension dimS.    □

The level equation

$$H_{w}\left(x\right)=c$$

can equivalently be written

$$\prod_{i=1}^{n}x_{i}^{w_{i}}=e^{c}.$$

Thus, the compatibility manifolds are intersections of generalized monomial level sets in the original positive variables. The map x↦logx merely sends these manifolds to the affine spaces

$$\log C\left(x^{0}\right)=\log x^{0}+S.$$

### 8.2. Relation with Siphon Geometry

For a boundary-regular GLV system, two different geometric structures are therefore present.

The siphon structure gives the boundary stratification by invariant coordinate faces,

$$F_{\Sigma }=\left\{x\geq 0:x_{i}=0\Leftrightarrow i\in \Sigma \right\},$$

and the transversal invasion exponents determine the relay graph between inhabited faces. This geometry is intrinsic to the multiplicative positive vector field and does not require an E-graph realization.

After an E-graph realization is fixed, Proposition 8 provides in addition the interior invariant foliation

$$R_{>0}^{n}=⋃C\left(x^{0}\right),\;C\left(x^{0}\right)=\exp\left(\log x^{0}+S\right).$$

Thus, the relay graph organizes the boundary supports, whereas the compatibility manifolds organize the dynamics inside the positive orthant.

### 8.3. Complex Balance and Global Convergence

We next give the complex-balanced stability result of Rojas La Luz, Yu and Craciun directly in the original variables. The proof below is the logarithmic-coordinate argument written without changing the state variables.

**Theorem** **10** (Complex-balanced global stability in the original variables [CB-intrinsic])**.** *Consider (18) and suppose that the fixed realization (G,κ) admits a complex-balanced equilibrium*

$$x^{*}\in R_{>0}^{n}.$$

*Then:*

*1.*  *the set of positive equilibria is*
  (22) $$E_{\mathrm{cb}}=\left\{x>0:\log x-\log x^{*}\in S^{⊥}\right\}=\exp\left(\log x^{*}+S^{⊥}\right);$$
*2.*  *the function*
  (23) $$V_{x^{*}}\left(x\right)=\sum_{i=1}^{n}{\left(\log\frac{x_{i}}{x_{i}^{*}}\right)}^{2}$$
  *is proper on $R_{>0}^{n}$ and satisfies*
  $${\dot{V}}_{x^{*}}\left(x\right)\leq 0,$$
  *with equality if and only if*
  $$x\in E_{\mathrm{cb}};$$
*3.*  *every compatibility manifold $C(x^{0})$ contains exactly one positive equilibrium, and this equilibrium is globally asymptotically stable relative to $C(x^{0})$.*

**Proof.**  Put

$$u=\log x-\log x^{*},\;a_{i}=y_{i}^{T}u,\;q_{i}={\left(x^{*}\right)}^{y_{i}}.$$

Since

$$x^{y_{i}}=q_{i}e^{a_{i}},$$

the logarithmic velocity may be written in edge form as

$$\frac{d}{dt}\log x=\sum_{(i,j)\in E}\kappa_{ij}q_{i}e^{a_{i}}\left(y_{j}-y_{i}\right).$$

Therefore

$$\frac{1}{2}{\dot{V}}_{x^{*}}=\sum_{(i,j)\in E}\kappa_{ij}q_{i}e^{a_{i}}\left(a_{j}-a_{i}\right).$$

Using

$$e^{a}\left(b-a\right)\leq e^{b}-e^{a},\;a,b\in R,$$

with equality if and only if a=b, gives

$$\frac{1}{2}{\dot{V}}_{x^{*}}\leq \sum_{(i,j)\in E}\kappa_{ij}q_{i}\left(e^{a_{j}}-e^{a_{i}}\right).$$

Regrouping this sum vertex by vertex yields

$$\begin{array}{c} \frac{1}{2}{\dot{V}}_{x^{*}}\leq \sum_{j}e^{a_{j}}\left(\sum_{i:(i,j)\in E}\kappa_{ij}q_{i}-q_{j}\sum_{\ell :(j,\ell )\in E}\kappa_{j\ell }\right). \end{array}$$

The expression in parentheses vanishes for every vertex *j* by complex balance at $x^{*}$. Hence

$${\dot{V}}_{x^{*}}\leq 0.$$

Equality holds exactly when

$$a_{j}=a_{i}\;\mathrm{for}\;\mathrm{every}\;\left(i,j\right)\in E,$$

that is,

$${\left(y_{j}-y_{i}\right)}^{T}u=0\;\mathrm{for}\;\mathrm{every}\;\mathrm{edge}\;\left(i,j\right),$$

or equivalently

$$u\in S^{⊥}.$$

Thus

$${\dot{V}}_{x^{*}}=0\Leftrightarrow \log x-\log x^{*}\in S^{⊥}.$$

Such a point is in fact an equilibrium. Indeed, $u\in S^{⊥}$ implies that $a_{i}$ is constant on every linkage class. Thus, on each linkage class, the vector $x^{Y}$ is a positive scalar multiple of ${\left(x^{*}\right)}^{Y}$. Since complex balance gives

$$A_{\kappa }{\left(x^{*}\right)}^{Y}=0$$

and $A_{\kappa }$ does not couple distinct linkage classes,

$$A_{\kappa }x^{Y}=0.$$

This proves (22).

The function $V_{x^{*}}$ is proper on $R_{>0}^{n}$, because a bounded sublevel set bounds every

$$\left|\log(x_{i}/x_{i}^{*})\right|$$

and therefore bounds every $x_{i}$ both away from 0 and away from +∞.

Finally,

$$\log C\left(x^{0}\right)=\log x^{0}+S,\;\log E_{\mathrm{cb}}=\log x^{*}+S^{⊥}.$$

Since

$$R^{n}=S⊕S^{⊥},$$

these two affine spaces have exactly one intersection point. Hence each compatibility manifold contains exactly one equilibrium. Properness of $V_{x^{*}}$ and LaSalle’s invariance principle then yield global asymptotic stability relative to that compatibility manifold.    □

**Remark** **33.** *The theorem separates two facts which are sometimes conflated. The compatibility manifold through $x^{0}$ is determined by the stoichiometric subspace of the chosen realization and exists whether or not the system is complex balanced. Complex balance supplies the second transverse foliation*

$$E_{\mathrm{cb}}=\exp\left(\log x^{*}+S^{⊥}\right)$$

*and the Lyapunov function (23). Their transversality in logarithmic space,*

$$S⊕S^{⊥}=R^{n},$$

*is precisely what gives one equilibrium per compatibility manifold.*

**Corollary** **22** (Deficiency zero and the full-dimensional case)**.** *Let (G,κ) be a fixed E-graph realization of (18).*

*1.*  *If G is weakly reversible and has deficiency zero, then for every κ>0 the realization is complex balanced. Consequently, every compatibility manifold contains exactly one positive equilibrium, globally asymptotically stable relative to that manifold.*
*2.*  *If, in addition,*
  $$S=R^{n},$$
  *then the positive orthant itself is the unique compatibility manifold, the positive equilibrium is unique, and it is globally asymptotically stable in $R_{>0}^{n}$.*

**Proof.**  The first assertion is the deficiency-zero theorem for polyexponential/E-graph systems of Rojas La Luz, Yu and Craciun [4]; Theorem 10 then applies.

If $S=R^{n}$, then $S^{⊥}=\left\{0\right\}$ and

$$C\left(x^{0}\right)=\exp\left(\log x^{0}+R^{n}\right)=R_{>0}^{n}.$$

The second assertion follows.    □

**Corollary** **23** (Positive diagonal rescaling)**.** *Let*

$$D=\mathrm{Diag}\left(d_{1},\ldots ,d_{n}\right),\;d_{i}>0,$$

*and consider the diagonally rescaled system*

(24) $$x^{\prime }=D\;\mathrm{diag}\left(x\right)YA_{\kappa }x^{Y}.$$

Suppose that the unscaled realization admits a complex-balanced positive equilibrium $x^{*}$.

*Then:*

*1.*  *the invariant compatibility manifolds of (24) are*
$$\begin{array}{c} C_{D}\left(x^{0}\right)=\exp\left(\log x^{0}+DS\right); \end{array}$$
*2.* 
$$V_{D}\left(x\right)=\sum_{i=1}^{n}\frac{1}{d_{i}}{\left(\log\frac{x_{i}}{x_{i}^{*}}\right)}^{2}$$
  *is a proper Lyapunov function and*
$${\dot{V}}_{D}\leq 0,$$
  *with equality precisely on the same equilibrium manifold*
$$E_{\mathrm{cb}}=\exp\left(\log x^{*}+S^{⊥}\right);$$
*3.*  *each $C_{D}\left(x^{0}\right)$ contains exactly one positive equilibrium, which is globally asymptotically stable relative to $C_{D}\left(x^{0}\right)$.*

**Proof.**  For

$$w\in {\left(DS\right)}^{⊥}$$

we have

$$Dw\in S^{⊥},$$

because

$$w^{T}Ds={\left(Dw\right)}^{T}s=0\;\forall \;s\in S.$$

Hence

$$\frac{d}{dt}w^{T}\log x=w^{T}DYA_{\kappa }x^{Y}={\left(Dw\right)}^{T}YA_{\kappa }x^{Y}=0,$$

which proves

$$C_{D}\left(x^{0}\right)=\exp\left(\log x^{0}+DS\right).$$

Furthermore,

$$\begin{array}{cc} {\dot{V}}_{D} & =2{\left(\log x-\log x^{*}\right)}^{T}D^{-1}\frac{d}{dt}\log x\\ & =2{\left(\log x-\log x^{*}\right)}^{T}YA_{\kappa }x^{Y}. \end{array}$$

This is exactly the derivative appearing in Theorem 10; therefore

$${\dot{V}}_{D}\leq 0,$$

with equality exactly when

$$\log x-\log x^{*}\in S^{⊥}.$$

The equilibrium set is unchanged because *D* is invertible.

It remains only to verify uniqueness of the intersection. We claim

$$\begin{array}{c} R^{n}=DS⊕S^{⊥}. \end{array}$$

The dimensions add to *n*, so it suffices to prove that the intersection is trivial. If

$$Ds\in S^{⊥}\;\left(s\in S\right),$$

then

$$0=s^{T}Ds.$$

Since D≻0, this forces s=0. Hence

$$DS\cap S^{⊥}=\left\{0\right\},$$

proving the direct-sum decomposition.

Thus, the affine spaces

$$\log x^{0}+DS\;\mathrm{and}\;\log x^{*}+S^{⊥}$$

meet in exactly one point. Properness of $V_{D}$ and LaSalle’s invariance principle complete the proof.    □

### 8.4. Complex Balance Does Not Imply Global Volterra Admissibility

The examples of Section 9.1, Section 9.2 and Section 9.3 are all consistent with the guess that complex balance forces $W(x^{*})\neq ⌀$. The next example shows that this guess is false already in dimension two.

**Theorem** **11** (Complex balance does not imply global Volterra admissibility [CBnotVolt])**.** There exists a reversible deficiency-zero graph-generated GLV system with a complex-balanced equilibrium $x^{*}$ for which

$$W(x^{*})=⌀.$$

**Proof.**  Consider the reversible embedded graph

$$y_{1}=\left(2,0\right)⇄y_{2}=\left(0,1\right)$$

with both rate constants equal to 1. Then

$$A_{\kappa }=\left(\begin{array}{cc} -1 & 1\\ 1 & -1 \end{array}\right),\;Y=\left(\begin{array}{cc} 2 & 0\\ 0 & 1 \end{array}\right),\;x^{Y}=\left(\begin{array}{c} x_{1}^{2}\\ x_{2} \end{array}\right).$$

There are two complexes, one linkage class, and

S=span{(−2,1)},dimS=1,

so the deficiency is

2−1−1=0.

The graph-generated system

$$x^{\prime }=\mathrm{Diag}\left(x\right)\;YA_{\kappa }x^{Y}$$

is

$$x_{1}^{\prime }=-2x_{1}\left(x_{1}^{2}-x_{2}\right),\;x_{2}^{\prime }=x_{2}\left(x_{1}^{2}-x_{2}\right).$$

Moreover $x^{*}=\left(1,1\right)$ is complex balanced, since

$${\left(x^{*}\right)}^{Y}={\left(1,1\right)}^{T},\;A_{\kappa }{\left(x^{*}\right)}^{Y}=0.$$

Let $a=(a_{1},a_{2})\gg 0$ and

$$L_{a}\left(x\right)=a_{1}G\left(x_{1}\right)+a_{2}G\left(x_{2}\right),\;G\left(u\right)=u-1-\log u.$$

Since $G^{\prime }\left(u\right)=\left(u-1\right)/u$, differentiation gives

(25) $${\dot{L}}_{a}\left(x\right)=\left(x_{1}^{2}-x_{2}\right)\left[a_{2}\left(x_{2}-1\right)-2a_{1}\left(x_{1}-1\right)\right].$$

Suppose that ${\dot{L}}_{a}\leq 0$ on all of $R_{>0}^{2}$. Fix t>0 and consider the point $\left(t,t^{2}\right)$. The first factor in (25) vanishes on the curve

$$x_{2}=x_{1}^{2}$$

and changes sign transversally across it. Therefore the second factor must vanish at every point of this curve; otherwise continuity would force ${\dot{L}}_{a}$ to take both signs in every sufficiently small neighbourhood of that point. Hence

$$a_{2}\left(t^{2}-1\right)-2a_{1}\left(t-1\right)=0\;\mathrm{for}\;\mathrm{every}\;t>0.$$

For t≠1 this is

$$a_{2}\left(t+1\right)=2a_{1}.$$

Taking, for example, t=2 and t=3 gives

$$3a_{2}=2a_{1},\;4a_{2}=2a_{1},$$

and therefore $a_{2}=0$, hence also $a_{1}=0$. This contradicts a≫0. Thus, no positive weight is globally admissible:

$$W(x^{*})=⌀.$$

   □

**Problem** **2** (Volterra admissibility on the compatibility manifold [volterra-compat])**.** Let $x^{*}$ be a complex-balanced equilibrium of a graph-generated GLV system, and let C be the compatibility manifold through $x^{*}$. Does there always exist a≫0 such that

$${\dot{L}}_{a}\leq 0\;\mathrm{on}\;C?$$

*Theorem 11 shows that global admissibility on the whole positive orthant is too strong in general. In the example of that theorem, however, the compatibility-restricted inequality holds for every a≫0.*

**Remark** **34** (Why complex balance is not enough)**.** The obstruction in Theorem 11 is not local. At $x^{*}=\left(1,1\right)$,

$$Df\left(x^{*}\right)=\left(\begin{array}{cc} -4 & 2\\ 2 & -1 \end{array}\right),$$

*whose eigenvalues are 0 and −5. Thus*

$$Df(x^{*})⪯0,$$

*and the necessary local diagonal-stability condition already holds with unit weights. What fails is the passage from this local quadratic condition to a Volterra inequality valid on the whole positive orthant.*

*Nor is the failure a contradiction with the complex-balance Lyapunov theorem. Complex balance supplies the log-quadratic function*

$$V_{x^{*}}\left(x\right)=\sum_{i}{\left(\log\frac{x_{i}}{x_{i}^{*}}\right)}^{2},$$

*which decreases along the graph-generated dynamics, whereas Definition 13 asks for a function from the different Volterra family*

$$L_{a}\left(x\right)=\sum_{i}a_{i}x_{i}^{*}G\left(x_{i}/x_{i}^{*}\right).$$

*Theorem 11 shows that the first certificate may exist while no member of the second family decreases on the whole positive orthant.*

*For the present graph,*

S=span{(−2,1)},

*so Proposition 8 gives*

$$\log C(x^{*})=S,$$

*and therefore*

$$C\left(x^{*}\right)=\left\{(e^{-2t},e^{t}):t\in R\right\}=\left\{x>0:x_{1}x_{2}^{2}=1\right\}.$$

*Writing*

$$q=e^{t}>0,\;x_{1}=q^{-2},\;x_{2}=q,$$

*Equation (25) gives*

$$\begin{array}{cc} {\dot{L}}_{a} & =\left(q^{-4}-q\right)\left[a_{2}\left(q-1\right)-2a_{1}\left(q^{-2}-1\right)\right]\\ & =-\frac{{\left(q-1\right)}^{2}\left(1+q+q^{2}+q^{3}+q^{4}\right)}{q^{4}}\left[a_{2}+\frac{2a_{1}\left(q+1\right)}{q^{2}}\right]\leq 0. \end{array}$$

*Thus, although*

$$W(x^{*})=⌀,$$

every a≫0 yields Volterra decrease on the compatibility manifold through $x^{*}$. Whether this compatibility-restricted statement holds for every complex-balanced graph-generated GLV system is precisely Problem 2.

## 9. The Examples of Rojas La Luz, Yu and Craciun [4], Revisited

Rojas La Luz, Yu and Craciun illustrate their theory on four worked systems: a cyclic three-species conversion network used throughout their paper’s early sections; a general cooperative quadratic GLV family recovering a classical global stability theorem (Goh [5]); a two-species system with higher-order (quadratic) interaction terms; and a fully explicit two-species system with non-polynomial right-hand side. Each system is complex balanced for the E-graph they exhibit, so their global stability theorem for complex-balanced GLV systems (the log-quadratic Lyapunov function $V\left(x\right)=\sum_{i}{\left(\log x_{i}-\log x_{i}^{*}\right)}^{2}$) applies to all four. This section answers questions (Q2)–(Q3) of the Introduction: does the Volterra Lyapunov function also certify global stability on each example, and, when it does, what do its weights encode?

Every system below is analyzed at its *interior* positive equilibrium, so Σ=⌀ throughout and the face-adapted Lyapunov function reduces to the classical Volterra function $V\left(x\right)=\sum_{k}a_{k}x_{k}^{*}G\left(x_{k}/x_{k}^{*}\right)$, G(u)=u−1−logu, with no linear penalty terms. The boundary/siphon machinery is not exercised here, since Rojas La Luz, Yu and Craciun’s theorems concern global stability on the whole positive orthant, not boundary invasion. Every sign verdict was checked by symbolic differentiation followed by constrained global optimization, or, when *f* is linear so that $\dot{V}$ is a pure quadratic form, by the sign of its Hessian eigenvalues—never asserted from the shape of the formula alone. Each example is presented through the same checklist, so that the four can be compared at a glance.

### 9.1. The Cyclic Triangle

The E-graph ${\hat{e}}_{1}⇌{\hat{e}}_{2}⇌{\hat{e}}_{3}⇌{\hat{e}}_{1}$ generates

$$\begin{array}{cc} x_{1}^{\prime } & =x_{1}\left(-\left(\kappa_{12}+\kappa_{13}\right)x_{1}+\kappa_{21}x_{2}+\kappa_{31}x_{3}\right),\\ x_{2}^{\prime } & =x_{2}\left(\kappa_{12}x_{1}-\left(\kappa_{21}+\kappa_{23}\right)x_{2}+\kappa_{32}x_{3}\right),\\ x_{3}^{\prime } & =x_{3}\left(\kappa_{13}x_{1}+\kappa_{23}x_{2}-\left(\kappa_{31}+\kappa_{32}\right)x_{3}\right). \end{array}$$

Weakly reversible/deficiencyyes (3-cycle)/0Complex balancedyes, for *every* κ>0 (Rojas La Luz, Yu and Craciun’s running example)Siphonsthe three singletons {1},{2},{3}Relay graphempty from any positive start; the stoichiometric subspace is two-dimensional, $S=\mathrm{span}\{e_{1}-e_{2},e_{2}-e_{3}\}$, and it foliates $R_{>0}^{3}$ into compatibility manifolds, each containing exactly one complex-balanced equilibriumHJ Lyapunovworks: Rojas La Luz, Yu and Craciun’s theorem that a complex-balanced steady state makes $V\left(x\right)=\sum_{i}{\left(\log x_{i}-\log x_{i}^{*}\right)}^{2}$ a global proper Lyapunov function, restated intrinsically in Section 3, Section 4, Section 5, Section 6, Section 7 and Section 8 aboveVolterra Lyapunovworks, but *not* with naive weightsWeights$a=\left(1,\frac{17}{11},1.7\right)=\left(1/x_{1}^{*},1/x_{2}^{*},1/x_{3}^{*}\right)$ on the tested instance; *not* a=1How obtainedcanonical left–right kernel formula $a_{i}=1/x_{i}^{*}$ (Corollary 24), not a feasibility solveAutomatic?yes, by Corollary 8—see Remarks 35 and 36

We tested the asymmetric instance $\kappa_{12}=2,\kappa_{21}=1,\kappa_{23}=3,\kappa_{32}=1,\kappa_{13}=1,\kappa_{31}=4$, with complex-balanced equilibrium $x^{*}=\left(1,\frac{11}{17},\frac{10}{17}\right)$. Since *f* is linear here, $\dot{V}$ is a pure quadratic form in $x-x^{*}$ and its sign is exactly the definiteness of a constant Hessian.

**Remark** **35.** Neither “obvious” weight works globally: with a=(1,1,1) or with $a_{k}=x_{k}^{*}$ the Hessian of $\dot{V}$ has eigenvalues of both signs (for our instance, respectively {−14.04,−10.15,+0.18} and {−10.53,−7.10,+0.57}), and indeed $\dot{V}\to +\infty$ along the ray $x=x^{*}+t\left(1,1,1\right)$. A valid weight exists nonetheless: solving the Sylvester conditions directly gives $a=(1,\frac{17}{11},1.7)$, with Hessian eigenvalues {−22.12,−13.25,0}, hence $\dot{V}\leq 0$ everywhere, with equality exactly on the eigenvector of the zero eigenvalue. The network is complex balanced but not reversible in the diagonal-symmetrizing sense—no diagonal a makes diag(a)M symmetric—so the “detailed balance” shortcut does not apply. A different structural shortcut does: Corollary 24 returns this exact weight with no feasibility solve at all.

**Corollary** **24** (Exact canonical weights for the cyclic triangle)**.** For the cyclic-triangle matrix M, $1^{T}M=0$ and $Mx^{*}=0$ with $x^{*}=\left(1,\frac{11}{17},\frac{10}{17}\right)$ (both verified directly: M’s three columns sum to zero, since the network only converts mass between species; and $Mx^{*}=0$ is the complex-balanced equilibrium condition). Corollary 9 therefore gives the exact admissible weight $a=\left(1/x_{1}^{*},1/x_{2}^{*},1/x_{3}^{*}\right)=\left(1,\frac{17}{11},1.7\right)$ directly—precisely the weight previously obtained from the Sylvester/SDP calculation, now explained rather than merely found.

**Proof.**  Immediate from Corollary 9 applied with ℓ=1, given $1^{T}M=0$ and $Mx^{*}=0$.    □

This is also a direct instance of Theorem 4: *M* is irreducible (the E-graph is a 3-cycle with all κ’s positive) with s(M)=0 (verified: the eigenvalues of *M* are $-6\pm \sqrt{2}\;i$ and 0), right Perron vector $w=x^{*}$ and left Perron vector π=1 (both eigenvalue-0 eigenvectors, unique up to scale by Perron–Frobenius), so $a^{P}=\pi /w=1/x^{*}$ recovers exactly the weight above; the exact edge-dissipation identity (5) at α=0, applied with these w,π, is precisely the edge-dissipation identity underlying Corollary 8’s proof, specialized to this instance.

**Remark** **36** (The SDP independently confirms the canonical weight)**.** *Corollary 24 already gives the exact weight in closed form; Theorem 3’s normalized program (4) for $M=\left(\begin{array}{ccc} -3 & 1 & 4\\ 2 & -4 & 1\\ 1 & 3 & -5 \end{array}\right)$ (the linear part of f on this instance) provides an independent check, and additionally certifies optimality and uniqueness in the $\min_{i}a_{i}$ sense:*

$$\varepsilon^{*}=\max_{a,\varepsilon }\varepsilon \;s.t.\;a_{1}+a_{2}+a_{3}=1,\;\;a_{i}\geq \varepsilon ,\;\;\mathrm{diag}\left(a\right)M+M^{T}\mathrm{diag}\left(a\right)⪯0.$$

*Solving this exactly (the three principal 2×2 minors and the 3×3 determinant of $-(\mathrm{diag}\left(a\right)M+M^{T}\mathrm{diag}\left(a\right))$ give four polynomial inequalities in a, in addition to the simplex constraints) gives*

$$\varepsilon^{*}=\frac{110}{467},\;a^{*}=\left(\frac{110}{467},\frac{170}{467},\frac{187}{467}\right),$$

exactly proportional to the weight $a=(1,\frac{17}{11},1.7)$ already found by hand: $a^{*}=\frac{110}{467}\cdot \left(1,\frac{17}{11},1.7\right)$ (verified exactly, e.g., $110\cdot \frac{17}{11}=170$, 110·1.7=187). At $a^{*}$ the 3×3 determinant vanishes exactly (the zero eigenvalue already reported), so $a^{*}$ lies on the boundary of the semidefinite region, not its interior—consistent with $\delta^{*}\leq 0$ in Corollary 3, i.e., no weight makes $\dot{V}$ strictly negative away from $x^{*}$ here (only semidefinite). The optimizer is unique: fixing $a_{1}=\varepsilon^{*}$ and eliminating $a_{3}=1-a_{1}-a_{2}$, the determinant condition becomes a cubic in $a_{2}$ alone that factors as ${\left(170-467a_{2}\right)}^{2}\left(934a_{2}-2849\right)$—a double root at $a_{2}=170/467$ and a second root at $a_{2}=2849/934\approx 3.05$, far outside the feasible range $0<a_{2}<357/467$; the double root is a tangency, not a crossing, so $a^{*}$ is an isolated point of the feasible boundary and the unique maximizer of $\min_{i}a_{i}$ on it. The required weight-finding routine for this example is therefore not a stub to be completed at all: the exact SDP recovers the canonical left–right kernel weight of Corollary 8, and that theorem supplies the structural shortcut specific to conservative irreducible Metzler systems, bypassing the SDP entirely.

Conclusion for this example: the Volterra function *does* certify global stability on each compatibility manifold, exactly as predicted by Lf.wl’s framework. The weight is not an isolated numerical finding but a canonical left–right kernel weight, given directly by Corollary 24 and confirmed as the exact SDP optimizer in Remark 36; what remains is a software task, not a mathematical gap: wiring lfWeights to solve (4) instead of falling back to $b_{j}=1$. This example is the clearest witness that the naive fallback is a genuine counterexample to global stability of *V*, even though the correct weight is now given in closed form.

### 9.2. The Cooperative Family (Goh’s Theorem)

Rojas La Luz, Yu and Craciun’s cooperative-GLV theorem recovers the classical global stability theorem for cooperative quadratic GLV systems $x^{\prime }=\mathrm{diag}\left(x\right)\left(r+Ax\right)$, r>0, *A* Metzler ($a_{ij}\geq 0$ for i≠j, $a_{ii}<0$), under the symmetric diagonal-dominance hypothesis of Corollary 5, for some d>0: $2d_{i}a_{ii}+\sum_{j\neq i}\left(d_{i}a_{ij}+d_{j}a_{ji}\right)\leq 0$ for every *i*. Their proof embeds the system into an E-graph on n+1 vertices (species plus the origin) and applies the deficiency-zero theorem.
Weakly reversible/deficiencyyes (star graph through the origin)/0Complex balancedyes, once a positive steady state $x^{*}$ exists and *d* satisfies the diagonal-dominance hypothesisSiphonsthe *n* singletonsRelay graphempty for the tested positive-equilibrium instancesCompatibility manifoldstrivial here: the E-graph’s stoichiometric subspace is all of $R^{n}$, so $x^{*}$ is the unique positive equilibrium (one compatibility manifold, the whole orthant)HJ Lyapunovworks: Rojas La Luz, Yu and Craciun’s cooperative-GLV theorem, recovering Goh’s 1977 criterion [5]Volterra Lyapunovworks, with the theorem’s *own* weightsWeightsa=d, the diagonal-dominance vector already required by the hypothesisHow obtainedclassical diagonal-dominance construction (Goh [5])—not solved anew, simply reusedAutomatic?yes, in the sense that *d* is already given; see Remark 37 and Proposition 3 below for why this is not a coincidence

The vector *d* in the hypothesis *is* a Volterra weight vector: it is exactly the classical diagonal-dominance/Volterra–Lyapunov-stability certificate for cooperative Lotka–Volterra systems, predating the complex-balance viewpoint by decades (Goh 1977 [5]). We verified this directly: for the symmetric instance r=(1,1,1), $A=(\begin{array}{ccc} -3 & 1 & 1\\ 1 & -3 & 1\\ 1 & 1 & -3 \end{array})$ with d=(1,1,1), and for the asymmetric instance r=(2,1,3), $A=(\begin{array}{ccc} -4 & 1 & 2\\ 2 & -5 & 1\\ 1 & 3 & -6 \end{array})$ with $d=(1,\frac{4}{9},\frac{11}{27})$ found by solving the diagonal-dominance inequalities directly, the Hessian of $\dot{V}$ at a=d is negative definite in both cases (eigenvalues {−8,−8,−2} and {−9.49,−6.28,−1.57} respectively): $\dot{V}<0$ strictly away from $x^{*}$, on all of $R_{>0}^{n}$.

**Remark** **37.** *Here the two theories do not merely agree in conclusion; they share the same certificate. The log-quadratic proof of the cooperative-GLV theorem uses d only through the E-graph construction; the Volterra proof uses d directly as the Lyapunov weight. This is the case where completing lfWeights would essentially reproduce, in the original variables, a decades-old classical argument—confirming the two mechanisms identified above (geometric organization via siphons; analytic global convergence via a Lyapunov function) are not competing explanations of the same fact, but the fact has independent proofs in both languages.*

**Remark** **38** (Comparison with the Hurwitz–Metzler kernel weight)**.** On the asymmetric instance above (A Metzler, Hurwitz with eigenvalues {−6.70±0.81i,−1.60}), three constructions give three genuinely distinct points of the admissible cone $W(x^{*})$, not the same certificate rediscovered: Goh’s diagonal-dominance vector $d=\left(1,\frac{4}{9},\frac{11}{27}\right)\approx \left(1,0.444,0.407\right)$ (Corollary 5); the Hurwitz–Metzler kernel weight $a^{\mathrm{kernel}}=v/w\approx \left(1.02,1.093,0.881\right)$, $v=-A^{-1}1$, $w=-A^{-T}1$ (Corollary 12, via Corollary 11); and the Perron weight $a^{P}\approx \left(1.024,1.088,0.850\right)$ (Definition 14, spectral abscissa s(A)≈−1.604). None is a scalar multiple of another (checked exactly for d against $a^{\mathrm{kernel}}$, numerically for $a^{P}$ against both), yet each independently makes $\mathrm{diag}\left(a\right)A+A^{T}\mathrm{diag}\left(a\right)$ negative definite.

### 9.3. The Higher-Order-Interaction Pair

Rojas La Luz, Yu and Craciun’s higher-order-interaction example considers

$$x_{1}^{\prime }=x_{1}\left(r_{1}-a_{11}x_{1}+a_{12}x_{2}-b_{1}x_{1}x_{2}\right),\;x_{2}^{\prime }=x_{2}\left(r_{2}+a_{21}x_{1}-a_{22}x_{2}-b_{2}x_{1}x_{2}\right),$$

$r_{i}>0$, $a_{ij},b_{i}\geq 0$, under the sufficient condition $\mathrm{sign}\left(r_{1}-a_{11}x_{1}^{*}\right)=\mathrm{sign}\left(a_{22}x_{2}^{*}-r_{2}\right)$ for a positive equilibrium $x^{*}$ to be complex balanced (via a rescaling by $d_{1},d_{2}>0$ and a 4-vertex square E-graph).
Weakly reversible/deficiencyyes (square E-graph)/0Complex balancedyes, on the tested instance (sign condition verified below)Siphonsthe two singletons {1},{2}Relay graphempty on the tested instanceCompatibility manifoldsthe E-graph’s stoichiometric subspace is $R^{2}$ after the $d_{1},d_{2}$ rescaling, so $x^{*}$ is the unique positive equilibriumHJ Lyapunovworks, via Rojas La Luz, Yu and Craciun’s diagonal-rescaling theoremVolterra Lyapunovworks, with the *plain* weightsWeightsa=(1,1)How obtainedno solving neededAutomatic?yes

Because *f* now has genuine quadratic terms, $\dot{V}$ is cubic in *x*, not a quadratic form, so no Hessian shortcut applies; we used constrained global optimization directly. We tested $r_{1}=3$, $a_{11}=2$, $a_{12}=1$, $b_{1}=1$, $r_{2}=2$, $a_{21}=1$, $a_{22}=3$, $b_{2}=1$, giving $x^{*}=\left(\frac{-2+\sqrt{37}}{3},\frac{-2+\sqrt{37}}{4}-\frac{1}{4}\right)\approx \left(1.361,0.771\right)$, which satisfies the sign condition ($r_{1}-a_{11}x_{1}^{*}\approx 0.278>0$, $a_{22}x_{2}^{*}-r_{2}\approx 0.312>0$). With a=(1,1)—no solving required—Maximize over ${\left[10^{-4},5000\right]}^{2}$ returns $\sup\dot{V}\approx 0$, attained exactly at $x^{*}$; along every ray to infinity we checked, $\dot{V}\to -\infty$. The Volterra function works here with the naive fallback, unlike Section 9.1.

The diagonal rescalings recurring across Section 9.1, Section 9.2 and Section 9.3—Goh’s vector *d*, the rescaling of Section 9.3, and Rojas La Luz, Yu and Craciun’s own diagonal-rescaling theorem—are all instances of Proposition 3. In particular, using the weight a=d on the original cooperative system is the same computation as using unit weights on the *D*-rescaled system, D=diag(d): Goh’s diagonal-dominance vector and the log-quadratic theory’s diagonal rescaling are the *same* object, seen respectively as a Volterra weight and as a vector-field rescaling.

### 9.4. Summary, and the Complex-Balance Question

In every example, the Volterra function certifies exactly the same global stability conclusion as Rojas La Luz, Yu and Craciun’s log-quadratic function—but, as Table 3 shows, this is not automatic. On the higher-order-interaction pair it costs nothing (unit weights); on the cooperative family it reduces to the classical diagonal-dominance certificate already required by the cooperative theorem’s own hypothesis; and on the triangle it is the canonical left–right kernel weight $a_{i}=1/x_{i}^{*}$ of Corollary 8 (Corollary 24), read off the equilibrium directly and independently confirmed as the exact optimizer of (4) in Remark 36.

The pattern across the table is a trade-off rather than a ranking: the logarithmic approach supplies its Lyapunov function automatically, while the Volterra approach exposes structure that the automatic construction leaves implicit. On every example, the Volterra weights expose a diagonal-dominance certificate, a canonical left–right kernel weight (Remark 18), or an exact transformation law relating the two (Proposition 3).

One loose end from Section 4.1 is worth closing first: whether the diagonal rescalings of Proposition 3 could enlarge the class of systems admitting Volterra weights.

**Corollary** **25** (Diagonal rescaling cannot create Volterra weights)**.** Let D≫0 be diagonal. Under either rescaling of Proposition 3, $W_{D}\left(x^{*}\right)\neq ⌀\Leftrightarrow W\left(x^{*}\right)\neq ⌀$: emptiness or nonemptiness of the admissible-weight cone is invariant under positive diagonal rescaling.

**Proof.**  For vector-field rescaling, $W_{D}\left(x^{*}\right)=D^{-1}W\left(x^{*}\right)$; for state rescaling, $W\left(Dx^{*}\right)=D^{-1}W\left(x^{*}\right)$ as well (Proposition 3(ii)). Either way $D^{-1}$ is a bijection of $R_{>0}^{n}$, so it maps ⌀ to ⌀ and a nonempty set to a nonempty set. □

Consequently, the question below needs no rescaling hedge: by Corollary 25, rescaling can transform a witness *a* but can never turn emptiness into nonemptiness or vice versa.

#### A Classification of Scalar GLV Systems by Lyapunov Type

For a GLV system with positive equilibrium $x^{*}$, say that HJ holds at $x^{*}$ if $x^{*}$ is a complex-balanced equilibrium of some graph-generated realization of the vector field, so that Theorem 10 supplies the logarithmic quadratic Lyapunov function on the corresponding compatibility manifold. Say that Volterra holds at $x^{*}$ if

$$W(x^{*})\neq ⌀.$$

HJ is a property of a graph realization, while Volterra admissibility is a property of the GLV vector field and the equilibrium $x^{*}$.

This gives four logically possible classes:

(*A*) HJ and Volterra;
(*B*) Volterra only;
(*C*) HJ only;
(*D*) neither HJ nor Volterra.

**Proposition** **9** (Jacobian obstruction to complex-balanced realizability)**.** *Let*

$$x^{\prime }=\mathrm{Diag}\left(x\right)f\left(x\right)$$

*be a graph-generated GLV system with a positive equilibrium*

$$x^{*}=1.$$

*If 1 is complex balanced for some Euclidean-graph realization*

$$f\left(x\right)=YA_{\kappa }x^{Y},$$

*then*

$$\begin{array}{c} Df\left(1\right)+Df{\left(1\right)}^{T}⪯0. \end{array}$$

*Consequently, if*

$$Df\left(1\right)+Df{\left(1\right)}^{T}$$

*has a positive eigenvalue, then 1 admits no complex-balanced Euclidean-graph realization of the vector field.*

**Proof.**  For a realization

$$f\left(x\right)=YA_{\kappa }x^{Y},$$

differentiation at 1 gives

$$Df\left(1\right)=YA_{\kappa }Y^{T},$$

because

$$D\left(x^{Y}\right){|}_{x=1}=Y^{T}.$$

Hence,

$$Df\left(1\right)+Df{\left(1\right)}^{T}=Y\left(A_{\kappa }+A_{\kappa }^{T}\right)Y^{T}.$$

Since $A_{\kappa }$ is a column Kirchhoff matrix,

$$1^{T}A_{\kappa }=0.$$

Complex balance at 1 gives

$$A_{\kappa }1=0.$$

Therefore

$$Q:=A_{\kappa }+A_{\kappa }^{T}$$

is symmetric, has nonnegative off-diagonal entries, and satisfies

Q1=0.

Thus, −Q is a symmetric graph Laplacian. Explicitly,

$$u^{T}Qu=-\sum_{i<j}\left({\left(A_{\kappa }\right)}_{ij}+{\left(A_{\kappa }\right)}_{ji}\right){\left(u_{i}-u_{j}\right)}^{2}\leq 0$$

for every *u*. Hence

Q⪯0.

It follows that

$$v^{T}\left[Df\left(1\right)+Df{\left(1\right)}^{T}\right]v={\left(Y^{T}v\right)}^{T}Q\left(Y^{T}v\right)\leq 0$$

for every *v*, proving

$$Df\left(1\right)+Df{\left(1\right)}^{T}⪯0.$$

   □

**Theorem** **12** (All four Lyapunov classes occur [classification-ABCD])**.** *Classes (A), (B), (C) and (D) all occur among GLV systems. Moreover, Classes (A), (B) and (D) already occur among affine GLV systems, while the present witness for Class (C) is nonlinear.*

**Proof.** 

(A) The affine cyclic-triangle and cooperative examples above admit complex-balanced graph realizations, so HJ holds. They also admit Volterra weights, by Corollary 24 and the Goh diagonal-dominance construction, respectively. Thus, Class (A) occurs.
(B) Let
  $$x^{*}=1$$
  and consider the affine GLV system
  $$x^{\prime }=\mathrm{Diag}\left(x\right)M\left(x-1\right),\;M=\left(\begin{array}{cc} -1 & 4\\ 1/8 & -1 \end{array}\right).$$
  The matrix *M* is irreducible, Metzler and Hurwitz, since
  $$\mathrm{tr}M=-2<0,\;\det M=\frac{1}{2}>0.$$
  More explicitly, with
  A=Diag(1,32),
  one obtains
  $$AM+M^{T}A=\left(\begin{array}{cc} -2 & 8\\ 8 & -64 \end{array}\right).$$
  Its leading diagonal entry is negative, and its determinant is
  128−64=64>0,
  so
  $$AM+M^{T}A≺0.$$
  By Theorem 3,
  (1,32)∈W(1),
  and Volterra holds.
  On the other hand,
  $$M+M^{T}=\left(\begin{array}{cc} -2 & 33/8\\ 33/8 & -2 \end{array}\right),$$
  whose eigenvalues are
  $$-2\pm \frac{33}{8}.$$
  In particular,
  $$-2+\frac{33}{8}=\frac{17}{8}>0.$$
  Therefore
  $$M+M^{T}0.$$
  Proposition 9 implies that no Euclidean-graph realization of this vector field can make $x^{*}=1$ complex balanced. Thus, HJ fails while Volterra holds, and the system belongs to Class (B).
(C) Theorem 11 gives a reversible deficiency-zero graph-generated GLV system with a complex-balanced equilibrium $x^{*}$ and
  $$W(x^{*})=⌀.$$
  Thus, HJ holds while Volterra fails, so Class (C) occurs.
(D) Let again $x^{*}=1$ and take
  $$M=I_{2},\;x^{\prime }=\mathrm{Diag}\left(x\right)\left(x-1\right).$$
  For every A=Diag(a)≫0,
  $$AM+M^{T}A=2A≻0.$$
  Hence, by Theorem 3,
  W(1)=⌀,
  so Volterra fails.
  Moreover,
  $$M+M^{T}=2I_{2}≻0.$$
  Proposition 9 shows that 1 cannot be complex balanced for any Euclidean-graph realization of the vector field. Hence, HJ also fails, and this system belongs to Class (D).

   □

**Remark** **39.** *Class (C) gives the rigorous separation established in this paper: complex balance may hold while global Volterra admissibility fails.*

Problem 2 asks for the finer relation on the compatibility manifold. If $x^{*}$ is complex balanced, one asks whether there exists a≫0 such that

$${\dot{L}}_{a}\leq 0$$

on the compatibility manifold through $x^{*}$. The example of Theorem 11 satisfies this compatibility-restricted property for every a≫0.

For affine GLV systems, Volterra admissibility is determined by the single semidefinite condition

$$\mathrm{Diag}\left(a\right)M+M^{T}\mathrm{Diag}\left(a\right)⪯0,\;a\gg 0.$$

The examples above exhibit three structural ways of producing feasible weights.

1. *Unit weights.* In Section 9.3,
  a=1
  satisfies the matrix inequality directly.
2. *Diagonal-dominance weights.* For the cooperative family, the classical Goh construction supplies a positive diagonal weight from the sign and dominance structure of the interaction matrix.
3. *Canonical Perron weights.* If *M* is irreducible Metzler with
  s(M)≤0,
  Theorem 4 gives
  $$a_{i}=\frac{\pi_{i}}{w_{i}},$$
  where
  $$Mw=s\left(M\right)w,\;\pi^{T}M=s\left(M\right)\pi^{T}.$$
  In the conservative case s(M)=0, this is the left–right kernel weight. For the cyclic triangle,
  $$w=x^{*},\;\pi =1,$$
  and therefore
  $$a_{i}=\frac{1}{x_{i}^{*}},$$
  as in Corollary 24.
  These three constructions produce feasible points of the same admissible-weight cone

$$W\left(x^{*}\right)=\left\{a\gg 0:\mathrm{Diag}\left(a\right)M+M^{T}\mathrm{Diag}\left(a\right)⪯0\right\}.$$

The SDP of Theorem 3 gives the general decision procedure. For the triangle, the normalized SDP also recovers the canonical Perron weight as the distinguished boundary point described in Remark 36.

For nonlinear generalized-polynomial GLV systems, global Volterra admissibility is governed by

$$\sum_{i}a_{i}\left(x_{i}-x_{i}^{*}\right)f_{i}\left(x\right)\leq 0,\;x\in R_{>0}^{n}.$$

Theorem 6 shows that the local Jacobian condition at $x^{*}$ gives only local information, while dissipativity of the averaged Jacobian $\bar{J}\left(x;x^{*}\right)$ is the exact global condition; a common diagonal certificate for the pointwise Jacobian family on the relevant convex invariant region (Corollary 18) is only a sufficient shortcut. Finite criteria for dissipativity of the averaged Jacobian form one of the main open directions of the nonlinear theory.

**Remark** **40.** *The two Lyapunov constructions encode different structures. A complex-balanced realization generates the logarithmic quadratic Lyapunov function from its graph and stoichiometric geometry. A Volterra certificate is attached directly to the GLV vector field and may encode diagonal dominance, Perron structure, or other properties of its interaction matrix.*

*Theorem 11 establishes one strict logical direction in this comparison: HJ can hold while global Volterra admissibility fails. Classes (B) and (D) require corresponding realization obstructions, supplied by Proposition 9.*

## 10. Conclusions

This paper develops a reaction-network and Lyapunov framework for generalized Lotka–Volterra systems by separating three complementary structures: boundary geometry, compatibility geometry, and Volterra–Lyapunov geometry.

For the boundary geometry, the multiplicative GLV form makes coordinate faces invariant and identifies the minimal siphons with the singleton coordinates in the boundary-regular scalar case. At a resident equilibrium $E_{\Sigma }$, the transversal Jacobian is diagonal and its entries are the invasion exponents

$$\lambda_{i}\left(E_{\Sigma }\right)=f_{i}\left(E_{\Sigma }\right),\;i\in \Sigma .$$

This gives the scalar form of the siphon and relay calculus and connects directly with the Perron spectral thresholds arising for transversal blocks in compartmental and block GLV systems.

For Volterra Lyapunov functions, the central object is the admissible-weight cone

$$W\left(x^{*}\right)=\left\{a\gg 0:\sum_{i}a_{i}\left(x_{i}-x_{i}^{*}\right)f_{i}\left(x\right)\leq 0\;\mathrm{for}\;\mathrm{all}\;x\in R_{>0}^{n}\right\}.$$

For affine GLV systems $f\left(x\right)=M\left(x-x^{*}\right)$, Theorem 3 gives the exact characterization

$$a\in W\left(x^{*}\right)\Leftrightarrow \mathrm{diag}\left(a\right)M+M^{T}\mathrm{diag}\left(a\right)⪯0,$$

and therefore reduces Volterra admissibility to a semidefinite feasibility problem. Theorem 4 gives a canonical solution in the irreducible Metzler case: if π and *w* are positive left and right Perron vectors, then

$$a_{i}=\frac{\pi_{i}}{w_{i}}$$

provides the Perron–Volterra weight whenever the Perron spectral bound is nonpositive. The corresponding proof gives an explicit graph-Laplacian dissipation identity. Goh’s diagonal-dominance construction appears as another explicit mechanism for producing points of the same admissible-weight cone.

The geometry of this cone provides additional information. In dimension two it is described explicitly by Theorem 5. In higher dimensions it may have a genuinely spectrahedral boundary, so the semidefinite formulation captures geometric features beyond polyhedral weight selection.

For nonlinear GLV systems, Theorem 6 identifies the local-to-global issue precisely: Volterra admissibility is exactly equivalent to dissipativity of the averaged Jacobian $\bar{J}\left(x;x^{*}\right)$ along the segment to $x^{*}$, while diagonal stability of the pointwise Jacobian family Df(x) on a convex invariant region (Corollary 18) is only a sufficient shortcut. This leads naturally to finite coefficient, graph-theoretic and sum-of-squares criteria for dissipativity of the averaged Jacobian.

Theorem 7 extends the Volterra construction to siphon faces of block GLV systems. A resident equilibrium *E* on $F_{\sigma }^{\circ }$ determines the transversal Jacobian

$$J_{\sigma }^{⊥}\left(E\right),$$

whose irreducible Frobenius blocks provide the corresponding Perron data. Under resident and transversal Perron–Volterra closing conditions, resident blocks contribute Perron-weighted entropy terms and missing blocks contribute positive linear Perron functionals; when the transversal closing coefficients have the signs of the corresponding invasion exponents, relay-sink stability together with compactness of the relevant sublevel sets yields convergence to the invariant set selected by the resident closing condition, and global convergence to the resident equilibrium when that set is a singleton. The scalar GLV construction is the one-dimensional specialization of this formula.

The Euclidean-graph realization supplies a complementary compatibility geometry. Its stoichiometric subspace determines first integrals and compatibility manifolds, while complex balance supplies the log-quadratic Lyapunov function of Rojas La Luz, Yu and Craciun. Theorem 11 separates this mechanism from global Volterra admissibility by exhibiting a reversible, deficiency-zero, complex-balanced GLV system with

$$W(x^{*})=⌀.$$

On the compatibility manifold of that example, Volterra decrease is recovered for every positive weight. This motivates Problem 2: characterize complex-balanced GLV systems admitting a compatibility-restricted Volterra function.

Two further directions emerge from the affine theory. First, Problem 1 asks for structural classes in which the optimal strict Volterra margin $\delta^{*}$ detects the Hurwitz boundary, and, in particular, the Hopf portion of that boundary. Second, the explicit affine SDP suggests computational implementations for constructing admissible weights, describing their cone, and testing the same weights across resident faces.

The resulting picture places siphons, transversal invasion, Perron theory, semidefinite diagonal stability, compatibility manifolds and Lyapunov functions in a common framework. Boundary geometry organizes the resident and invading components, Perron theory supplies canonical block directions, the admissible-weight cone organizes Volterra certificates, and complex balance supplies a complementary compatibility-based stability mechanism. These structures provide the basis for extending the scalar GLV theory developed here to the block GLV, gradostat, epidemic and immuno-virology systems considered in [26].

## Figures and Tables

**Table 1:** All three Lyapunov derivatives are governed by the same matrix $Q=\left(\begin{array}{cc} b_{21}a_{11} & b_{12}b_{21}\\ b_{12}b_{21} & b_{12}a_{22} \end{array}\right)$, positive definite iff Δ>0 (Theorem 8). Both singleton siphons are inhabited (Definition 12) in every chamber, by $E_{2}$ and $E_{1}$ respectively, since $r_{1},r_{2}>0$ throughout this sub-family; what varies across chambers is which resident equilibrium attracts, not which siphons are inhabited.

Parameter Chamber	Inhabited Siphons	Attractor	Lyapunov Function	Relay Interpretation
$\lambda_{2|1}<0$	{1},{2}	$E_{1}$	$L_{1}=b_{21}x_{1}^{*}G\left(x/x_{1}^{*}\right)+b_{12}y$	2 cannot invade 1
$\lambda_{1|2}<0$	{1},{2}	$E_{2}$	$L_{2}=b_{21}x+b_{12}y_{2}^{*}G\left(y/y_{2}^{*}\right)$	1 cannot invade 2
$\lambda_{2|1}>0,\;\lambda_{1|2}>0$	{1},{2}	$E_{12}$	$L_{12}=b_{21}x^{*}G\left(x/x^{*}\right)+b_{12}y^{*}G\left(y/y^{*}\right)$	mutual invasion

**Table 2:** Inhabited siphons (Definition 12): both singletons {1},{2} remain inhabited by the axial equilibria $E_{2}=\left(0,r_{2}/a_{22}\right)$, $E_{1}=\left(r_{1}/a_{11},0\right)$ throughout, since $r_{1},r_{2}>0$ is unchanged across Section 6.2 (verified for the counterexample instance: $E_{1}=\left(4.84,0\right)$, $E_{2}=\left(0,4.94\right)$); the higher-order terms $b_{i}xy$ vanish identically on both axes, so they affect only the interior equilibrium count, never siphon habitation.

Parameter Sector	Inhabited Siphons	Positive Equilibria	$\lambda_{2|1}$	$\lambda_{1|2}$	Volterra Conclusion
$q_{12},q_{21}\geq 0$ (original)	{1},{2}	unique interior $E_{12}$ (Theorem 9)	+	+	no boundary function decreasing (Section 6.2.1); interior certified in Section 9.3
$q_{12},q_{21}<0$, generic	{1},{2}	unique interior, or two (±multiplicity, Theorem 9)	sector-dependent	sector-dependent	general sufficient theorem open (Section 6.2.3)
counterexample instance	{1},{2}	two interior + $E_{2}$ (three stable/saddle pairs)	+	−	multistationarity: no single global Volterra function possible

**Table 3:** For each example of Rojas La Luz, Yu and Craciun reproduced here, which minimal siphons are inhabited (Definition 12: the species-*i*-extinct face carries a positive equilibrium of the remaining species), whether the Horn–Jackson (HJ) and Volterra Lyapunov functions certify global stability, which Volterra weights were used, and whether finding them was automatic, what mechanism (Theorem 4, Corollary 5, or direct/unit weighting) they instantiate, and what remains open. On the triangle, all three singleton faces support only the origin (verified symbolically, Section 9.1), consistent with its empty relay graph.

Example	Inhabited Siphons	HJ	Volterra	Weights	Automatic?	Interpretation	Open Issue
Triangle (Section 9.1)	none	√	√	$a_{i}=1/x_{i}^{*}$	√	left–right kernel weight	–
Cooperative (Section 9.2)	{1},{2},{3} (all)	√	√	Goh’s *d*	√	diagonal dominance	–
HOI pair (Section 9.3)	{1},{2} (both)	√	√	$a_{i}=1$	√	none	–

## Data Availability

The original contributions presented in this study are included in the article. Further inquiries can be directed to the corresponding author.
